# New Force-Field for Organosilicon Molecules in the Liquid Phase

**DOI:** 10.1021/acsphyschemau.1c00014

**Published:** 2021-08-27

**Authors:** Miguel Jorge, Andrew W. Milne, Maria Cecilia Barrera, José R. B. Gomes

**Affiliations:** † Department of Chemical and Process Engineering, University of Strathclyde, 75 Montrose Street, Glasgow G1 1XJ, United Kingdom; ‡ CICECO − Aveiro Institute of Materials, Department of Chemistry, University of Aveiro, Campus Universitário de Santiago, 3810-193 Aveiro, Portugal

**Keywords:** Organosilicates, Silica, Molecular model, Molecular Dynamics, Polarization

## Abstract

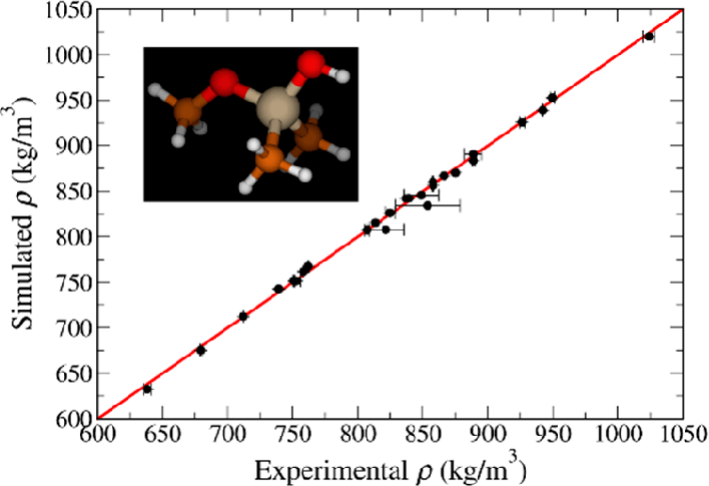

In this paper, we present a new molecular model that can accurately predict thermodynamic liquid state and phase-change properties for organosilicon molecules including several functional groups (alkylsilane, alkoxysilane, siloxane, and silanol). These molecules are of great importance in geological processes, biological systems, and material science, yet no force field currently exists that is widely applicable to organosilicates. The model is parametrized according to the recent Polarization-Consistent Approach (PolCA), which allows for polarization effects to be incorporated into a nonpolarizable model through *post facto* correction terms and is therefore consistent with previous parametrizations of the PolCA force field. Alkyl groups are described by the United-Atom approach, bond and angle parameters were taken from previous literature studies, dihedral parameters were fitted to new quantum chemical energy profiles, point charges were calculated from quantum chemical optimizations in a continuum solvent, and Lennard-Jones dispersion/repulsion parameters were fitted to match the density and enthalpy of vaporization of a small number of selected compounds. Extensive validation efforts were carried out, after careful collection and curation of experimental data for organosilicates. Overall, the model performed quite well for the density, enthalpy of vaporization, dielectric constant, and self-diffusion coefficient, but it slightly overestimated the magnitude of self-solvation free energies. The modular and transferable nature of the PolCA force field allows for further extensions to other types of silicon-containing compounds.

## 1 Introduction

Silicon is the second most abundant element in the Earth’s crust, behind oxygen, accounting for 28.8% by mass.^1^ It is therefore no surprise that compounds involving silicon play a major role in geological, biological, and industrial processes. For example, silicon eroded from rocks and minerals is dissolved in water under the form of orthosilicic acid, i.e., Si(OH)_4_, reaching concentrations of a few parts per million in oceans and rivers. Some of that silicon is then harvested by organisms like diatoms and sponges to yield intricately beautiful hierarchical structures in a process called biosilicification.^2,3^ Silicon is also at the heart of the computer revolution, as the core material in the manufacture of semiconductor chips.^4^ Organosilicon molecules (e.g., dichlorodimethylsilane) are used as precursors in the synthesis of silicone rubbers and polymers, including the widely used polydimethylsiloxane.^5^ As a final example, of more direct relevance to our research, organosilicon compounds like tetraethoxysilane are key precursor species in the synthesis of porous materials like zeolites,^6,7^ periodic mesoporous silicas,^8,9^ organosilicas,^10,11^ and bioinspired silica.^3,12^

Understanding, controlling, and optimizing many of the above processes and applications relies on obtaining molecular-level insight on how silicon-containing molecules interact with each other and/or with other compounds in a liquid or solution environment—for example, biosilicification, porous silica synthesis, and silicone rubber polymerization all take place in solution. Molecular modeling techniques are ideally suited to shed light on such processes,^7,9,11,12^ but they require accurate, robust, and versatile classical interaction potential models (also called force-fields). It is therefore somewhat surprising that, to the best of our knowledge, no force-field that is generally applicable to organosilicon compounds currently exists. There have been attempts in the past to develop generally applicable models to describe silica-based solids, e.g., to model adsorption in zeolites and other porous silica materials^13−15^ or to describe crystalline and amorphous silica surfaces.^16,17^ However, it is not straightforward to assume that these models can be directly transferred to silicon-containing molecules in the liquid state, since both the intramolecular (e.g., conformational flexibility) and intermolecular (e.g., polarization) environments will be quite different from those in the solid state.

The absence of a general force-field does not preclude the existence of molecular simulation studies focusing on individual molecules or subsets of the large organosilicon “family”. In particular, there have been several attempts to develop atomistic or united-atom (UA) models of polydimethylsiloxane (PDMS) chains (see, e.g., refs (18 and 19) and references therein). Again, however, it is not clear to which extent these models developed specifically for polymers are directly transferable to smaller organosilicate molecules, and only very few efforts in this direction have been reported. Striolo and co-workers carried out simulations of polyhedral oligomeric silsesquioxanes (e.g., octamer cages with alkyl substituents) dissolved in alkanes^20,21^ or in PDMS^22^ to characterize their structural and dynamic properties. Their model was based on a combination of the TraPPE force-field for alkanes^23^ and a previous model for PDMS,^18^ which led to a rather awkward combination of 12-6 and 9-6 functional forms for the repulsion/dispersion interactions. Polyakov et al.^24^ later tested the model of Striolo et al. in simulations of tetraethylsilane and ditertbutylsilane, and found that it yielded a density for the former molecule that was about 5% too high and an enthalpy of vaporization that was about 15% too low. They therefore refined the Lennard-Jones parameters of the Si sites to obtain a more reasonable match with experimental data.

Some previous work has focused on modeling silicate or organosilicate molecules that act as precursors in porous material synthesis. As early as 2001, Pereira et al.^25^ developed a model for tetramethoxysilane (TMOS) and tetraethoxysilane (TEOS) in the pure liquid state, by combining dispersion/repulsion parameters from a zeolite model of Hill and Sauer^26^ with empirically adjusted point charges. They obtained reasonable agreement with experimental densities, but the enthalpy of vaporization of both compounds was significantly overestimated, by about 18 kJ/mol.^25^ Later, the same authors applied their model to simulate precursor solutions in silica sol–gel synthesis, which included orthosilicic acid monomers (i.e., Si(OH)_4_) and dimers (i.e., Si_2_O(OH)_6_), showing that those species tended to aggregate in water/alcohol solutions.^27^

Jorge and co-workers^28,29^ later adapted the model of Pereira et al.^25,27^ to fit with more commonly used functional forms (e.g., harmonic rather than quartic bond and angle potentials; Lennard-Jones 12-6 instead of 9-6 function), and adjusted the point charges to match quantum mechanical calculations on a wide range of silica oligomers,^30^ including anionic species. The atomistic model for silicic acid and for higher silica oligomers (both neutral and deprotonated) was combined with a model for cationic ammonium surfactants to describe the initial stages in the synthesis of mesoporous silica materials,^28,29^ and with a model for amine surfactants to study the synthesis of bioinspired silica.^31^ The same group subsequently extended their approach to model organosilicate precursors in the synthesis of mesoporous organosilica materials.^32,33^ The results from all these atomistic simulations formed the basis for the development of a coarse-grained model of precursor solutions in the synthesis of several classes of nanoporous silica materials.^9,11,12,34−36^

Azenha and co-workers^37^ used a similar approach to Jorge et al.,^28,29^ i.e., adapting and extending the model of Pereira et al.^25,27^ to be used with more generally applicable functional forms, in this case the OPLS-AA framework,^38^ and applied their model to describe the sol–gel synthesis of imprinted xerogels. Their simulations included cyclic silicate trimers in the neutral form, as well as an organosilicate derivative of the cyclic trimer. As a validation test, they compared the density of two alkoxysilanes (TMOS and (3-propylaminophenyl)-trimethoxysilane) against experimental data, obtaining reasonable agreement (deviations of ∼7% and ∼2%, respectively). The same approach was later applied to a more complex imprinting solution containing an imidazolium-based organosilicate.^39^

The studies described above evidence the need for a widely applicable, versatile, and robustly validated molecular model for organosilicon compounds in the field of materials science, and this is the main aim of the present paper. Because reliable experimental data for organosilicon molecules is rather scarce, at least compared to more commonly studied organic molecules, a significant effort of data collection, analysis, and curation was undertaken, as described in detail in section 2.1. A further motivation for this work is the realization that “standard” nonpolarizable force-fields suffer from systematic shortcomings in predictions of phase-change properties (e.g., enthalpy of vaporization, solvation free energy) and electronic properties (e.g., dielectric constant, dipole moment) due to the neglect of explicit polarization.^40−47^ Inspired by theoretical developments in approximate treatments of polarization effects, we have begun to parametrize a new class of nonpolarizable models for liquids and solutions, which we call PolCA—standing for Polarization-Consistent Approach.^48,49^ The force-field developed here for organosilicates is entirely consistent with the new PolCA paradigm, and similarly to our recent work, it is based on a United-Atom description of alkane groups.^23,50^

Because parametrizing a force field for all possible organosilicate molecules is a formidable task, in the present paper, which is meant as the first step toward this ultimate goal, we focus on a subset of this class of molecules. Namely, we limit ourselves to tetrahedrally substituted organosilicates with alkyl (≡Si–C_*x*_H_*y*_), alkoxy (≡Si–O–C_*x*_H_*y*_), and/or silanol (≡Si–OH) substituent groups. This excludes several molecules of practical relevance, such as silane (SiH_4_), all molecules with −SiH_3_, −SiH_2_, and −SiH groups, molecules with aryl substituents, as well as chlorinated organosilanes. Our choice is motivated by the key role played by alkylsilanes, alkoxysilanes, and silanols in the sol–gel chemistry of silica,^51,52^ as well as by parametrization convenience—i.e., maintaining compatibility with the PolCA UA force field for alkanes^48^ and alcohols^49^ while keeping the number of parameters that need to be determined down to a manageable level. The paper is organized as follows. Section 2 contains a brief description of the methodology, starting with the collection and curation of experimental data, followed by computational details, and ending with the force field parametrization strategy. More comprehensive details of each of these methodological aspects are presented in the Supporting Information. In section 3, we compare the results obtained with the new PolCA force field for organosilicates against experimental data for each class of compounds considered. We finish the paper with conclusions and recommendations for future work.

## 2 Methodology

### 2.1 Experimental Data

A key requirement for the adequate parametrization and validation of a molecular model is the availability of accurate experimental data for a wide variety of physical properties and chemical compounds. While such data for organic compounds is widely available and easy to find, the same is not true for organosilicates. Therefore, a significant effort was devoted to the collection, analysis, and curation of experimental data for organosilicates. In line with the PolCA approach, we used the bulk density (ρ) and the enthalpy of vaporization (Δ*H*_Vap_) of selected liquids to fit the required parameters for each class of compounds, as described in detail in section 2.4. The models were then validated by predicting the density and enthalpy of vaporization of other compounds not used in the parametrization step, as well as the dielectric constant (ε) and the self-solvation free energy (Δ*G*_Solv_), when available. The self-diffusion coefficient (*D*) of tetramethylsilane was also used for validation, but no diffusion data for other relevant compounds was found in the literature. Apart from the dielectric constant, other electronic properties were needed to estimate the polarization corrections, as described in section 2.2. This requires each molecule’s gas-phase dipole moment (μ), polarizability (α), static dielectric constant, and refractive index (*n*_D_).

The full details regarding the collection and analysis of experimental data are provided in section S1 of the Supporting Information. This section also presents a full list of chemical compounds considered in this work, including their full name, chemical formula, and abbreviated nomenclature. Here, we describe only the most important points. Data was collected from several property compilation databases, including those of Yaws,^53,54^ Bažant et al.,^55^ Rochow,^56^ Eaborn,^57^ Voronkov et al.,^58^ Chickos and Acree,^59^ Stull,^60^ Maryott and Smith,^61^ as well as the National Institute of Standards and Technology webbook.^62^ However, whenever possible, the original source of the data was sought and reanalyzed if necessary. Therefore, data was also collected from a number of publications focusing on individual molecules or small “families” of organosilicates.^24,63−92^ When multiple data points for each property/compound pair were available, an average and standard deviation were calculated. In those cases, the uncertainty is reported as twice the standard error of the mean (i.e., with error bars corresponding to ∼95% confidence interval). For several property/compound pairs, however, only one data point could be found, in which case no uncertainty is reported.

**Figure 1:**
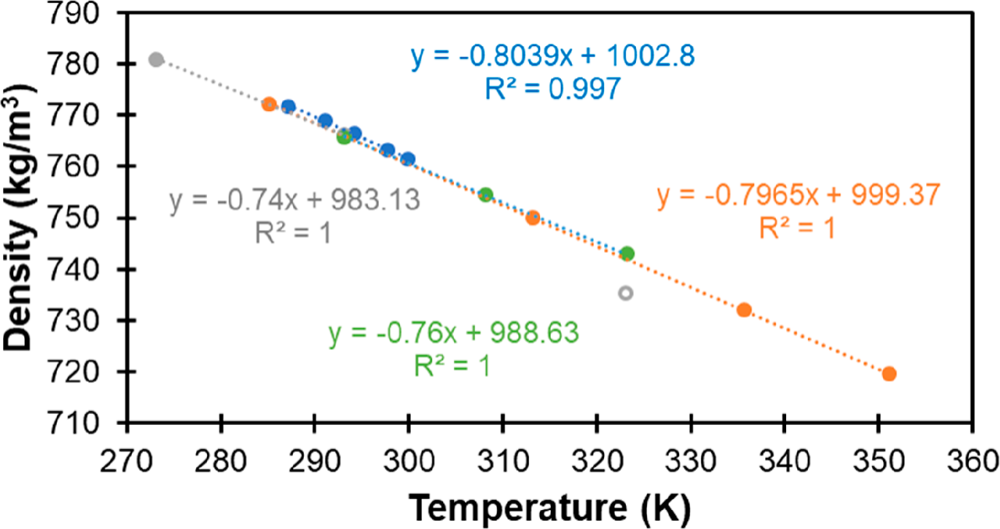
Linear fits to the data for density of tetraethylsilane as a function of temperature obtained from Yokoyama et al.^64^ (blue), Bažant et al.^55^ (green), Sugden and Wilkins^68^ (orange), and Whitmore et al.^69^ (gray). The linear fit equations and correlation coefficients are show in the insets with the corresponding color code. Notice that the last point at 323 K from Whitmore et al. (open circle) was excluded from the fit because it falls outside the observed trends.

When a given literature source reported data at a temperature other than 298 K, it was corrected by data fitting or other approximate schemes, as described in detail in section S1 of the Supporting Information. Hence, except where explicitly noted, all the experimental data points considered in the model parametrization and validation stages correspond to a temperature of 298 K. For the density, this was the only necessary analysis step. In Figure 1, we show an example of this analysis for the case of tetraethylsilane.

As we can see, all the data series are quite consistent with the exception of the last point from Whitmore et al.,^69^ which was therefore removed from the analysis. Each linear fit equation was then used to estimate the density at 298 K for each of the corresponding data sets. A correction factor was also derived from the average of the slopes shown in Figure 1, allowing us to correct density values that were reported at temperatures different from 298 K.^56,57^ As shown in Table S2, these two corrected values, the four values obtained from the linear fits in Figure 1, and three additional data points reported at 298 K^24,53,67^ are quite consistent with each other, yielding an average density of 761.1 kg/m^3^ with a very small uncertainty of 0.4 kg/m^3^.

**Figure 2:**
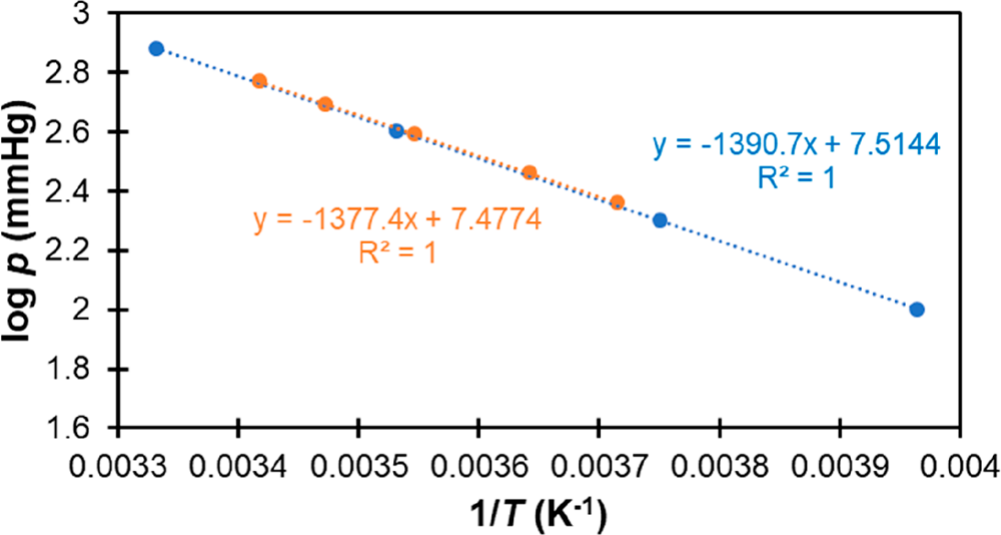
Logarithm of the vapor pressure as a function of inverse temperature for tetramethylsilane. The data were obtained from Stull^60^ (blue) and Aston et al.^76^ (orange). The equations and correlation coefficients for the corresponding linear fits are shown as insets with the same color code.

Literature data for the enthalpy of vaporization of organosilicon compounds was most often estimated from measurements of the vapor pressure over a range of temperatures, although some data (most notably that of Voronkov et al.^58^) was obtained from calorimetry measurements. Whenever raw data for the vapor pressure was available, we carried out our own calculations following the procedure described by Chickos and Acree.^59^ Specifically, the data were fitted to an equation of the form
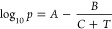
1with pressure (*p*) in mmHg and temperature (*T*) in Kelvin. Once the data for the vapor pressure was fitted to eq 1, Δ*H*_Vap_ was calculated from
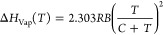
2where *R* is the ideal gas constant. Whenever possible, a narrow range of temperatures centered around 298 K was selected for the fitting procedure. This was not always possible, however, in which cases the calculated enthalpy needed to be corrected for the temperature difference. To achieve this, we implemented a step-by-step process that depended on the data availability for each particular compound, as described in detail in section S1.2 of the Supporting Information. An example is shown in Figure 2 for tetramethylsilane. Data for the vapor pressure as a function of temperature obtained from Aston et al.^76^ and Stull^60^ are plotted together with the corresponding linear fits. From the slopes of these fits, the enthalpy of vaporization was calculated from eq 2. Note that, although in principle it would have been possible to combine all the vapor pressure data sets together and perform a single fit, we opted to treat each data set independently, thus obtaining an estimate of experimental uncertainty directly from values of the target property (i.e., the enthalpy of vaporization).

Despite the fact that both vapor pressure data sets are consistent with each other, they span different ranges of temperature. As such, the average temperatures that correspond to each enthalpy value are different (275.6 K for Stull and 281.3 K for Aston et al.). For this particular molecule, the temperature correction factor was estimated from the parameters of a temperature-dependent correlation reported by Yaws,^54^ which was shown to be consistent with an estimate obtained from the heat capacity difference (see Supporting Information). The temperature-corrected enthalpies derived from Figure 2 are consistent with each other and with three additional values reported at 298 K,^54,58,77^ leading to an average of 25.2 ± 0.5 kJ/mol for Δ*H*_Vap_.

Self-solvation free energies (i.e., when the solute and solvent are the same compound) were calculated from the experimental vapor pressure at 298 K using eq 3:
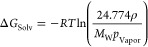
3where ρ is the experimental density in kg/m^3^, *M*_W_ is the molecular weight in g/mol, and *p*_Vapor_ is the vapor pressure in bar. The vapor pressures for each compound of interest were obtained from the coefficients of eq 1, when available, or otherwise from the boiling point and the enthalpy of vaporization after integrating the Clausius–Clapeyron equation between two points on the vapor/liquid equilibrium curve (see Supporting Information section S1.3 for details). Finally, electronic properties were obtained directly from literature sources without further analysis, except for the calculation of averages and uncertainties when more than one value was available. A complete set of experimental properties used in model development and validation is provided in Table S70.

### 2.2 Computational Details

Molecular Dynamics (MD) simulations were carried out with GROMACS version 5.1.2,^93,94^ using the Verlet leapfrog algorithm^95^ to integrate the equations of motion with a time step of 2 fs. Simulation boxes were cubic with periodic boundary conditions in all directions and a box length of ∼3.1 nm (average box lengths for each individual molecule varied, but were always close to this value). Liquid phase simulations were run in the *NpT* ensemble, using a V-rescale thermostat^96^ with a coupling constant of 0.1 ps to keep the temperature constant at 298 K, and a Parrinello–Rahman barostat^97^ with a coupling constant of 2 ps and a compressibility of 4.5 × 10^–5^ m^3^/bar to keep the pressure constant at 1 bar. The Lennard-Jones potential was cut off at 1.0 nm, with long-range dispersion corrections added to both energy and pressure. Long-range electrostatic interactions were accounted for by using the particle-mesh Ewald method.^98^ Constraints were applied on all bonds using the LINCS algorithm.^99^ Most liquid-phase MD simulations were run for a total of 10 ns for the force field parametrization stage and 50 ns for the validation stage, with the first 0.5 ns of each run being discarded for equilibration purposes. The exceptions were molecules containing silanol groups, which exhibited larger fluctuations in thermodynamic properties and hence were run for twice as long (i.e., 20 ns for parametrization and 100 ns for validation).

The bulk liquid density was directly calculated from the average volume of the simulation box using the GROMACS analysis tool *gmx energy*. This tool was also applied to calculate the average potential energy required to estimate the enthalpy of vaporization, following eq 4:
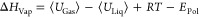
4In this equation, *U*_Liq_ is the potential energy per mole in the liquid phase, *U*_Gas_ is the potential energy per mole in the gas phase, and *E*_Pol_ is a correction term to account for the effects of polarization (see below for details). The angular brackets denote ensemble averages. Gas-phase MD simulations to compute *U*_Gas_ made use of the same protocol as bulk liquid simulations, except that no barostat was used (i.e., simulations were run in the *NVT* ensemble), the simulation boxes contained a single molecule, and no periodic boundary conditions or cutoff radius were applied, hence replicating a vacuum environment.

A unique feature of the PolCA approach is to explicitly account for the effects of polarization in the calculation of phase-change properties (e.g., enthalpy of vaporization or solvation free energy) and electronic properties (e.g., dielectric constant). For phase-change properties, this takes the form of an additive energy term, *E*_Pol_, given by
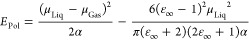
5where μ_Gas_ is the dipole moment of the molecule in the gas phase, μ_Liq_ is the dipole moment of the molecule in the liquid phase, α is the polarizability of the molecule, and ε_∞_ is the infinite-frequency dielectric constant of the liquid. The first term on the right-hand side of eq 5 is the positive distortion energy, which represents the energetic cost of distorting the wave function of the molecule when it is transferred from an unpolarized state in the gas to a polarized state in the liquid. It is similar to the correction first applied by Berendsen et al.^100^ when developing the widely used SPC/E model of water, except that in Berendsen’s expression, the average dipole moment of the molecular model was used as a proxy for μ_Liq_. The second term on the right-hand side is the negative electronic energy, describing the favorable interaction between the polarized molecule and the electronic clouds of the surrounding liquid molecules. This term accounts for the purely electronic effects of polarization, which are not captured in classical nonpolarizable force fields.^40,41^ The expression for the electronic energy term was first proposed by Leontyev and Stuchebrukhov^41^ and is based on representing the surrounding solvent by a uniform dielectric continuum characterized by the infinite-frequency dielectric constant of the liquid, here described by the simple Onsager model for a dipole in a spherical cavity.^101^ Use of ε_∞_ in eq 5 ensures that only the purely electronic polarization response of the system is taken into account, since the nuclear response is already described implicitly in the classical model parameters (namely, the effective point charges of the molecule).^40^ We note that eq 5 is identical to the expression used in our previous work,^49^ except that it has the opposite sign—i.e., the polarization corrections are defined here with reference to a transfer from the gas to the liquid phase.

To apply eq 5, μ_Gas_ and α were obtained directly from experimental data sources (see Supporting Information section S1.4), while ε_∞_ was calculated as the square of the experimental index of refraction of the liquid measured at the sodium D-line frequency. Although μ_Liq_ has been estimated for water from scattering experiments,^102^ we are not aware of experimental values for any other liquids, including organosilicates. It is also possible to calculate μ_Liq_ from quantum mechanical (QM) calculations that replicate the liquid environment (e.g., *ab initio* molecular dynamics or quantum mechanics/molecular mechanics methods). However, such calculations are very computationally demanding and to our knowledge have only been performed for simple molecules like water (see ref (103) and references therein) and methanol (see, e.g., ref (104)). Therefore, and in line with our previous work,^49^ we estimate μ_Liq_ from an analytical expression derived by applying the Onsager dielectric continuum model to the surrounding liquid:
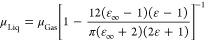
6Equation 6 makes use of the experimental dielectric constant of the liquid (ε) and was derived by treating the radius of the cavity self-consistently so that it is eliminated from the final expression.^41^ The reader is referred to previous publications by our group and others for further details on the calculation and interpretation of polarization energies.^40−42,49,103^ The estimated liquid dipole moments and polarization energies for the entire set of organosilicate molecules considered here are reported in Table S69.

The self-diffusion coefficient was calculated, by applying the Einstein equation, from the slope of the mean-square displacement averaged over the entire MD trajectory, calculated using the *gmx msd* tool of GROMACS. It is well-known that the calculation of *D* is sensitive to finite-size effects.^105^ To account for these effects, we calculated *D* for three values of the simulation box length (*L*), plotted *D* as a function of 1/*L*, and extrapolated to infinite box size. The results of this procedure for tetramethylsilane are shown in Figure S47. We note that these finite-size corrections were only carried out for tetramethylsilane, since experimental values of *D* were not available for any other organosilicate molecule.

The static dielectric constant of the liquid was calculated from MD simulations using the GROMACS tool *gmx dipoles*, which applies eq 7
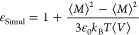
7where *k*_B_ is Boltzmann’s constant, ε_0_ is the vacuum permittivity, *V* is the volume of the simulation box, and *M* is the dipole moment of the entire simulation box. The dielectric constant is also affected by the neglect of explicit polarization in fixed-charge force fields,^45−47^ in the sense that eq 7 makes use of the effective dipole moment of the model instead of considering the dipole moment of the real liquid and neglects the purely electronic response of the medium. To mitigate those shortcomings, we apply a simple correction to the dielectric constant obtained from the MD simulations (ε_Simul_)
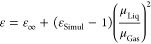
8It has been shown that application of eq 8 to the results obtained from MD simulations using several nonpolarizable force fields can eliminate systematic errors due to the neglect of explicit polarization and yield predictions that are in good agreement with experimental values for a wide variety of pure liquids^46^ as well as mixtures.^47^

Self-solvation free energies were calculated using the Bennett Acceptance Ratio (BAR) method,^106^ with details described in our previous publication.^49^ In brief, we decoupled the Lennard-Jones (LJ) and electrostatic free energy contributions separately, using 16 λ-states for LJ (0, 0.05, 0.1, 0.15, 0.2, 0.25, 0.3, 0.35, 0.4, 0.45, 0.5, 0.6, 0.7, 0.8, 0.9, and 1) and 6 λ-states for electrostatics (0, 0.2, 0.4, 0.6, 0.8, and 1). The intermediate lambdas were chosen based on a calculation of their relative entropy, which is an effective measure of phase-space overlap between adjacent λ-states.^107^ A soft-core function was used to avoid instabilities close to the noninteracting state,^108^ with parameters sc-power = 1 and sc-sigma = 0.3. Sc-alpha was 0.5 for the LJ term, while sc-alpha = 0 was used for the electrostatic component. The MD simulation protocol was the same as for the calculation of bulk liquid properties, except for the use of a leapfrog stochastic dynamics integrator.^109^ Convergence tests were carried out as described in ref (49) to determine the optimal length of each simulation run. For all molecules, each λ-state of the LJ component was run for a total of 10 ns. However, the length of each λ-state run for the electrostatic component varied for each class of molecules—it was 5 ns for alkylsilanes, 10 ns for alkoxysilanes, and 20 ns for silanols. Polarization corrections were applied to the solvation free energies by adding *E*_Pol_ obtained from eq 5 to the results of the MD simulations. The uncertainty in all simulated properties was estimated by block averaging and is reported as error bars representing approximately a 95% confidence interval on the mean.

Quantum Mechanical (QM) calculations, necessary to obtain point charges and to parametrize the torsional potential terms (see section 2.4 for details), were carried out with Gaussian 09,^110^ using the hybrid B3LYP exchange-correlation functional^111,112^ and Dunning’s aug-cc-pVTZ basis set.^113^ This protocol has been shown to yield accurate predictions for electronic properties of molecules.^114,115^ Calculations to derive point charges for organosilicate molecules made use of the IEFPCM method,^116^ which was used to include implicitly the solvent effects under the self-consistent reaction field (SCRF) formalism with the default parameters from Gaussian 09 except where noted.

### 2.3 PolCA Force Field

In line with our previous work, the model for organosilicate compounds is based on the United-Atom paradigm, which means that aliphatic hydrogens are not described explicitly, but their effect is implicitly included in the parametrization of the adjacent carbon atoms—in other words, each CH_*x*_ group is considered as a single interaction site. The force field is a sum of several energy terms, namely, bond stretching, angle bending, dihedral torsion, 12-6 Lennard-Jones (representing dispersion and repulsion interactions) and fixed point charges described by the Coulomb potential. The functional forms for these interactions, described below, were chosen to maintain compatibility with the PolCA force field for other molecules^48,49^ as well as to ensure the model could be applied in most standard molecular simulation software packages.

Bond stretching terms are normally applied to all atoms connected by one covalent bond and are described by a simple harmonic function, given in eq 9:
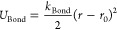
9where *k*_Bond_ is the bond force constant, *r*_0_ is the equilibrium bond length, and *r* is the actual distance between the two bonded atoms. Consistently with previous parametrizations of the PolCA force field,^48,49^ as well as with the TraPPE force field^23^ on which it is based, all bonds were treated as rigid by enforcing constraints during the MD simulations. This effectively means that the force constant in eq 9 is taken as infinite, and therefore only the bond length needs to be specified.

Angle bending terms were described by a harmonic function, given in eq 10:
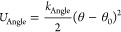
10where *k*_Angle_ is the angle force constant, θ_0_ is the equilibrium bond angle, and θ is the actual angle between the three bonded atoms.

Torsion energies were described by the Ryckaert-Bellemans function, eq 11, which is a sum of powers of cosines of the dihedral angle (φ), and where *C*_*i*_ are the coefficients for each corresponding cosine power term.
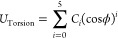
11In several force fields, the dihedral energy is supplemented by including, in total or in part, Lennard-Jones and Coulomb interactions between atoms separated by three bonds—i.e., the so-called 1-4 interactions between atoms on the two extremes of a dihedral angle. Although such interactions may be necessary in some particular cases, e.g., to avoid excessive attraction or repulsion caused by highly charged groups, our approach has been to eliminate 1-4 interactions from the force field as it improves transferability and reduces the degree of coupling between different parameters of the model.

Nonbonded interactions are described by two potential energy terms, one to describe repulsion and dispersion, in the form of the ubiquitous 12-6 Lennard-Jones potential, eq 12, and another to describe permanent electrostatic interactions between atoms with fixed point charges, eq 13. In those equations, *r*_*ij*_ is the distance between any pair of interaction sites, *σ*_*ij*_ is the LJ collision diameter, *ε*_*ij*_ is the LJ potential energy well depth, *q*_*i*_ is the point charge on site *i*, and ε_0_ is the vacuum permittivity.
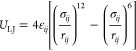
12
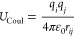
13The LJ parameters for interactions between different types of sites were calculated using the standard Lorentz–Berthelot combining rules:
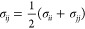
14
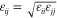
15

### 2.4 Parameterization Approach

Given the relative scarcity of experimental data for organosilicate molecules in the liquid phase, as discussed in section 2.1 and associated Supporting Information, it was important to keep the number of fitting parameters of the model to a minimum. It was also our priority to maintain compatibility with the PolCA approach and its previous parametrization efforts.^48,49^ For these reasons, only data for the density and the enthalpy of vaporization of selected compounds was used in the parametrization stage, and a step-by-step process was implemented, as described below. Furthermore, parameters for aliphatic hydrocarbons^48^ and alkyl alcohols^49^ were carried over from previous work—in practice, however, only the alkane parameters were relevant for the molecules considered here. Any relevant bonded parameters were also carried over from the TraPPE force field, as done in previous PolCA parametrizations.^48,49^ In the remainder of this paper, we have used the following nomenclature for atom types. Si is a tetrahedrally substituted silicon atom; where relevant, we use a superscript to denote the number of oxygenated substituent groups (e.g., Si^3^ is a silicon atom with three oxygenated substituents—i.e. silanol, alkoxy, or siloxane—and one remaining alkyl substituent). O_H_ is a silanol oxygen atom (i.e., part of a Si–O–H group). O_C_ is an alkoxy oxygen atom (i.e., part of a Si–O–CH_*x*_ group). O_B_ is a “bridging” siloxane oxygen atom (i.e., part of a Si–O–Si group). C is an alkyl or alkoxy carbon atom, while H represents any hydrogen atom.

In the PolCA model for alcohols,^49^ point charges on each atom were optimized empirically by fitting to experimental data. Here, instead, we opted to assign point charges to specific atom types by carrying out QM calculations on solvated molecules, and fit only the LJ parameters to experimental data. This significantly simplifies the fitting procedure and prevents overfitting, given the limited set of experimental properties available. As described in section 2.2, QM calculations were carried out for selected compounds solvated in a dielectric continuum model. This approach yields point charges that effectively take into account the electronic environment of the surrounding liquid, and are hence well-suited for models that aim to predict liquid-phase properties.^117^ The molecules were selected in order to span all the necessary environments (namely, alkylsilane, alkoxysilane, and silanol), while ensuring a reasonable computational expense. Due to the lack of organosilicate solvent parameters in the IEFPCM implementation of Gaussian 09, alternative solvents were selected among those available based on the similarity of the static dielectric constant and/or molecular structure. Table S71 lists the solvents selected for each organosilicate solute, together with their dielectric constants.

After each molecule was optimized in the corresponding solvent, point charges were calculated using the Density Derived Electrostatic and Chemical (DDEC) method,^118^ and the results are reported in Table 1. We also fitted charges using the CHelpG procedure,^119^ but those led to several chemical inconsistencies, mainly due to the presence of buried atoms (most prominently, all the tetrahedrally substituted Si atoms). DDEC yielded more chemically realistic charges and therefore was adopted as the method of choice. We note also that, in line with the UA approach, we added together all the hydrogen and carbon charges in each alkane CH_*x*_ group, so only the aggregate charge is reported in Table 1.

**Table 1:** Point Charges on Each Atom Obtained from DDEC Calculations on Organosilicate Molecules Optimized in an IEFPCM Continuum Model with Solvents Listed in Table S71^a^

molecule	*q*_Si_	*q*_CH_3_(Si)_	*q*_CH_2_(Si)_	*q*_CH_3_(CH_2_Si)_	*q*_O_	*q*_H_	*q*_CH__*_x_*(O)_	*q*_CH_3_(CH_2_O)_
Met4Si	0.960	–0.240	–	–	–	–	–	–
Eth4Si	0.975	–	–0.2875	0.0438	–	–	–	–
Met3SiOH	1.451	–0.329	–	–	–0.908	0.444	–	–
Eth3SiOH	1.237	–	–0.333	0.0605	–0.849	0.4295	–	–
Met6Si2O	1.458	–0.3335	–	–	–0.915	–	–	–
SiOMet4	1.852	–	–	–	–0.719	–	0.256	–
SiOEth4	1.878	–	–	–	–0.758	–	0.326	–0.0375
M3SiOE	1.390	–0.320	–	–	–0.678	–	0.294	–0.046
M2ESiOE	1.329	–0.319	–0.332	0.0583	–0.664	–	0.2915	–0.0448
ME2SiOE	1.258	–0.309	–0.3282	0.0519	–0.645	–	0.289	–0.0404
E3SiOE	1.194	–	–0.322	0.0554	–0.638	–	0.287	–0.0432

a Note that charges on aliphatic hydrogens have been added together with those of the adjacent carbon, in line with the UA approach. Molecule abbreviations are described in the Supporting Information.

From an analysis of the charge values in Table 1, several trends are apparent. First of all, the variability of the charge on the silicon atom is significant, depending strongly on the nature of the substituent groups. Charges on the outward-facing substituent groups, in contrast, are much more stable across different molecules. It is also clear that charges on alkane groups that are connected to other alkane groups (e.g., terminal CH_3_ groups in ethyl substituents—fifth and ninth columns in Table 1) are very small. This supports our approach of assigning a value of zero to the charge of those groups, which essentially behave as neutral UA sites in alkanes. In those cases, the net charge for the alkyl chain is placed on the first CH_*x*_ group (e.g., the group adjacent to the Si atom in alkylsilanes).

Based on these observations, and with the aim of keeping the model as simple as possible (i.e., avoiding the proliferation of different atom types), we arrived at the charge assignment shown in Table 2. The charges for each substituent group were calculated by averaging charges in similar functional groups from Table 1 and then rounding to the second decimal point. For each molecule, the silicon charge then needs to be calculated after assigning the charges on all substituent atoms so as to ensure overall charge neutrality. This approach satisfies our criteria for simplicity, while providing a good approximation to the original DDEC point charges reported in Table 1. In fact, the RMSD between the force field charges and the original DDEC charges is below 0.05, with the largest deviation of 0.15 being observed for the Si atom of SiOEth4.

**Table 2:** Atom Types and Point Charge Assignments for the PolCA Organosilicate Force Field^a^

atom type	description	*q*
**-CH**_*x*_-Si-(CH_*x*_)_3_	Any alkyl group connected to Si in an alkysilane molecule	–0.24
**CH**_**3**_-Si-O-	CH_3_ group connected to Si in a silanol, alkoxysilane, or siloxane molecule	–0.32
-**CH**_**2**_-Si-O-	CH_2_ group connected to Si in a silanol, alkoxysilane, or siloxane molecule	–0.27
-Si-O_C_-**CH**_***x***_-	Any alkyl group connected to an alkoxysilane oxygen	+0.25
-Si-**O**_**C**_-CH_*x*_-	Alkoxysilane oxygen	–0.68
-Si-**O**_**H**_-H	Silanol oxygen	–0.88
-Si-O_H_-**H**	Silanol hydrogen	+0.44
-Si-**O**_**B**_-Si-	Siloxane “bridging” oxygen	–0.88
**-Si-**	Tetrahedrally substituted silicon	bespoke

a The atom type in question is shown in bold. The silicon charges for each molecule are calculated after assigning all other charges by enforcing the charge neutrality constraint.

With point charges having been assigned, we are in a position to address the bonded potential parameters. The bond lengths were extracted from the literature, either from experimental determinations^120^ or QM calculations,^121,122^ as summarized in Table 3 and discussed in detail in section S3.1 of the Supporting Information. Equilibrium angle values and force constants were taken from the work of Grigoras and Lane^121^ and are shown in Table 4. Although those authors used an anharmonic angle bending potential, we confirmed that restricting this to the harmonic component alone, i.e., eq 10, led to a good description of the region around the energy minimum (see Figure S21). Furthermore, we assumed that the force constant for the C–Si–O_C_ angle, which was not given by Grigoras and Lane, was the same as that for the C–Si–O_H_ angle. All remaining bond and angle parameters were taken from the TraPPE force field.^23,123,124^

**Table 3:** Bond Lengths for the Organosilicate Molecules Considered in This Work

bond	length (nm)	source
Si–C	0.1875	(120)
Si–O_H_	0.1653	(121)
Si–O_C_	0.1656	(121)
Si–O_B_	0.1640	(122)
C–C	0.154	(23)
C–O_C_	0.141	(123)
O_H_−H	0.945	(124)

**Table 4:** Angle Bending Parameters for the Organosilicate Molecules Considered in This Work

angle	θ_0_ (deg)	*k*_Angle_ (kJ mol^–1^ rad^–2^)	source
C–Si–C	112.0	656.2	(121)
Si–C–C	111.5	726.5	(121)
C–Si–O_H_	107.4	774.6	(121)
Si–O_H_–H	115.5	257.8	(121)
O_H_–Si–O_H_	104.4	872.6	(121)
Si–O_B_–Si	149.5	61.3	(121)
Si–O_C_–C	124.8	298.1	(121)
C–Si–O_C_	111.0	774.6	(121)
O_C_–Si–O_C_	105.7	795.0	(121)
C–C–C	114.0	519.7	(23)
O_C_–C–C	112.0	418.2	(123)

Regarding the torsion terms, we have decided to parametrize these from in-house QM calculations, the details of which are described in section 2.2. Although we were able to find parameters for some relevant dihedrals in the literature, most of them include 1-4 interactions, which goes against our parametrization approach. We therefore fitted the coefficients of eq 11 to DFT energy scans for each dihedral angle. For each molecule, the atoms pertaining to the dihedral of interest were rotated incrementally in steps of 30° over the entire 360° range, leading to a total of 12 DFT calculations per dihedral. In each calculation, all the heavy atoms as well as the hydrogen atoms belonging to hydroxyl groups (which are explicitly represented in our force field) were kept fixed, while all the aliphatic hydrogen atom positions were optimized. This is in keeping with the philosophy of a UA model, where the aliphatic hydrogens are implicitly described through the parameter set for the adjacent carbon atom. In each case, an effort was made to eliminate, as far as possible, contributions from other types of interatomic interactions. For example, the bond and angle terms were always kept constant over each scan, and so could be disregarded from the calculation. Furthermore, molecules were chosen to minimize the contribution of LJ and Coulomb interactions between atoms separated by four or more bonds. Whenever this was not possible, those contributions were calculated according to the corresponding classical force field expressions (eqs 12 and 13) and subtracted from the DFT energy profiles. Full details of the dihedral fitting procedure, including raw data and fits for each individual case, are presented in section S4 of the Supporting Information.

**Figure 3:**
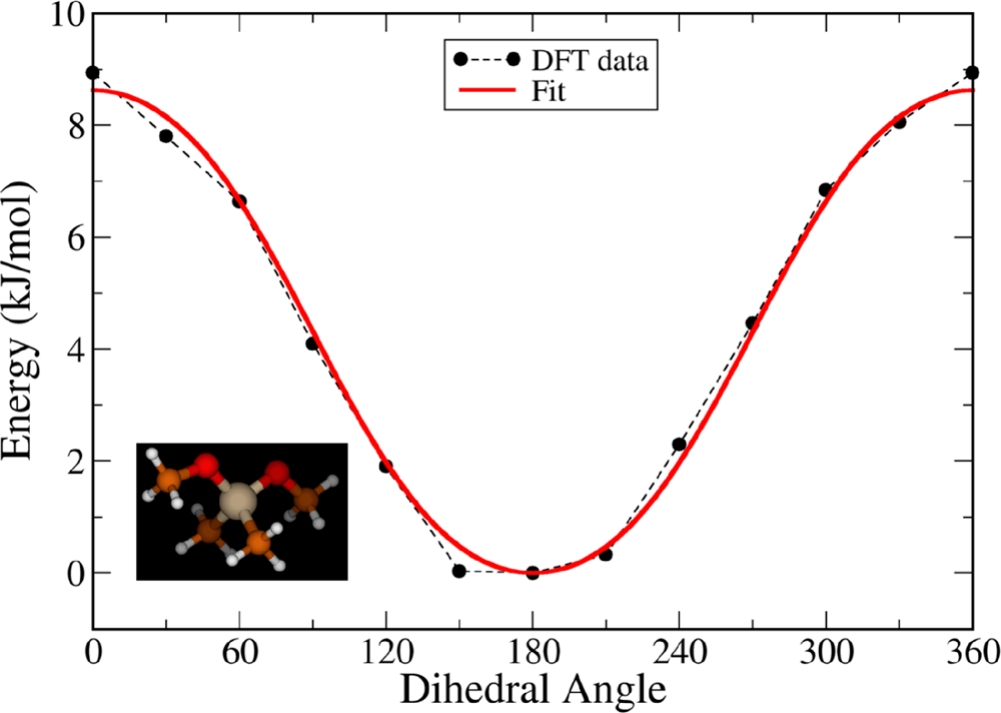
Comparison between the normalized DFT energy profile (black circles) and the classical torsion potential (red line) for the C–O_C_–Si–O_C_ dihedral. The black dashed line is a guide to the eye. The inset shows a ball-and-stick representation of the dimethyldimethoxysilane molecule used to parametrize this dihedral angle. Carbon atoms are shown in brown, silicon in cream, oxygens in red, and hydrogens in white.

As an example, we show the DFT data and the fit to eq 11 for the C–O_C_–Si–O_C_ dihedral angle in Figure 3, where the inset shows the dimethyldimethoxysilane molecule selected for the parametrization of this dihedral. In this molecule, there are 1–5 interactions between the terminal carbons in both methoxy groups. Since both of these atoms are bonded to an oxygen atom, they are charged (see Table 2), and therefore, both LJ and Coulomb contributions were nonzero. The total DFT energy scan also contains contributions from two C–Si–O_C_–C dihedrals, which were subtracted from the energy profile, having been previously parametrized (see Supporting Information). As we can see from Figure 3, the classical torsion potential shows a good fit to the DFT energy profile. The full set of dihedral parameters used in this work are shown in Table 5.

**Table 5:** Final Set of Torsion Parameters (in kJ/mol) for All Dihedrals Considered in This Work

dihedral	*k*_T,0_	*k*_T,1_	*k*_T,2_	*k*_T,3_	*k*_T,4_	*k*_T,5_
CCSiC	1.224	3.672	0.0	–4.895	0.0	0.0
CSiO_C_C	1.364	4.093	0.0	–5.457	0.0	0.0
CCSiO_C_	0.692	2.456	0.437	–3.416	0.0	0.0
CCO_C_Si	7.949	7.892	2.723	–18.563	0.0	0.0
CO_C_SiO_C_	4.314	4.803	0.0	–0.489	0.0	0.0
CSiO_H_H	0.870	2.600	0.0	–3.470	0.0	0.0
CCSiO_H_	0.801	2.760	0.508	–3.615	0.0	0.0
O_C_SiO_H_H	10.189	2.939	0.0	6.918	0.0	0.0
CO_C_SiO_H_	13.021	0.350	–39.801	–25.132	39.605	31.769
O_H_SiO_H_H	10.071	6.167	2.322	6.236	0.0	0.0
CCSiO_B_	0.692	2.456	0.437	–3.416	0.0	0.0
CO_C_SiO_B_	4.314	4.803	0.0	–0.489	0.0	0.0
CSiO_B_Si	0.0503	0.151	0.0	–0.201	0.0	0.0
O_C_SiO_B_Si	10.607	0.420	0.0	10.187	0.0	0.0
O_B_SiO_B_Si	10.607	0.420	0.0	10.187	0.0	0.0
O_H_SiO_B_Si	14.871	8.170	0.0	6.700	0.0	0.0

The final stage of parametrization was to determine the optimal Lennard-Jones parameters for each type of atom. As mentioned above, alkane parameters were taken from the PolCA model,^48^ so the atom types that had to be parametrized were as follows: Si, O_C_, O_H_, and O_B_. We adopted a step-by-step approach to determine these parameters, by fitting the LJ parameters for each atom type in turn. In each case, the parameters were designed to match the density and enthalpy of vaporization of selected compounds, as described in detail below. To find the optimal parameters for each atom type, we applied the same optimization algorithm as in our previous work for alcohols.^49^ In brief, a learning grid was created using simulations with varying LJ parameters for the atom of interest, and then, meta-models were generated by fitting each property’s learning set to a second-order equation with cross-interaction terms
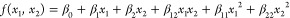
16where *x*_1_ and *x*_2_ are coded values of σ and ε, respectively. These meta-models predict how the fitted properties change with the input parameters and were used to define the objective function
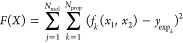
17where *N*_mol_ and *N*_prop_ are the number of molecules and target properties used in the optimization, respectively, *f*_*k*_(*x*_1_*, x*_2_) is the value predicted by the meta-model, and *y*_exp_*k*__ is the experimental value. This objective function was minimized using a steepest descent algorithm with a variable step length and a maximum number of iterations equal to 4000. The lowest value from these iterations was used as the initial point for a second optimization which used smaller step lengths and a maximum number of iterations equal to 100.

We started by fitting the parameters for an Si atom with four alkyl substituents. For that purpose, we chose to match the density and enthalpy of vaporization of tetramethylsilane (Met4Si) and tetraethylsilane (Eth4Si) and used properties of other alkylsilanes for validation purposes. After preliminary tests with a broad range of values for σ and ε, we carried out a full optimization with a grid composed of the following parameter values: σ ∈ [0.5;0.525;0.55;0.575;0.6;0.625] and ε ∈ [0.05;0.075;0.1;0.125;0.15;0.175]. This returned values of σ = 0.58 nm and ε = 0.108 kJ/mol for the Si atom in alkylsilanes.

**Figure 4:**
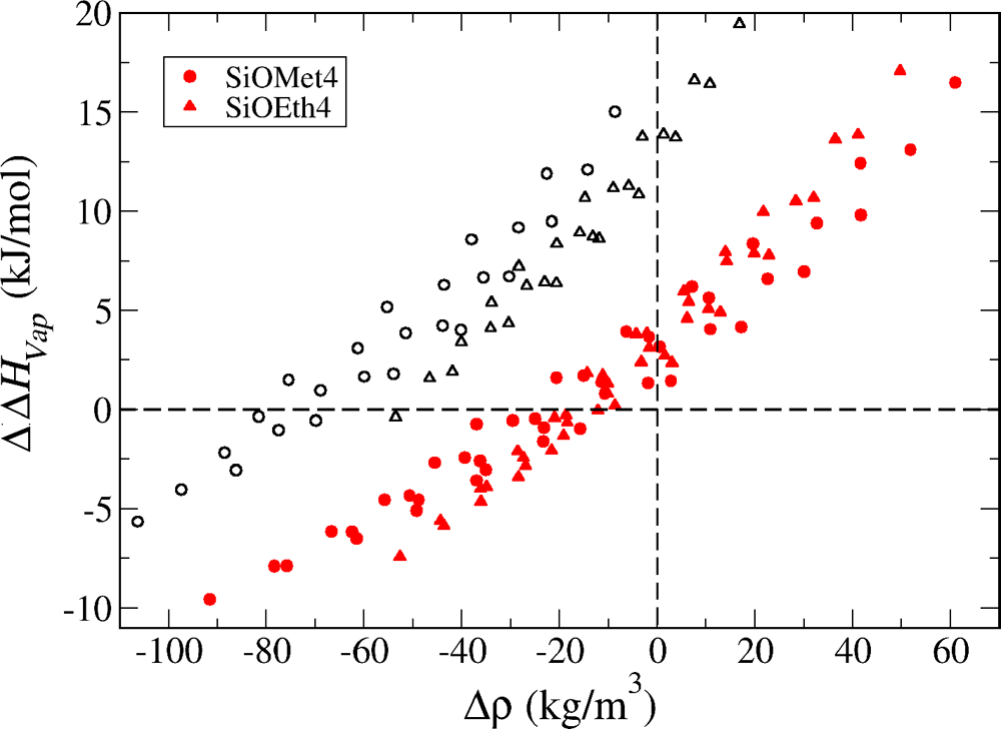
Absolute deviations between simulated and experimental properties for each of the target alkoxysilane molecules (circles for tetramethoxysilane and triangles for tetraethoxysilane), obtained with the same grid of parameters for σ_O_ and ε_O_. Black open symbols are results obtained with the default σ_Si_ and ε_Si_ parameters obtained for alkylsilanes, while red full symbols were obtained with σ_Si_ scaled down by 20% (see text for details).

Once those parameters were determined, we moved on to parametrize the alkoxy oxygen atom, O_C_, by fitting to the density and enthalpy of vaporization of tetramethoxysilane (SiOMet4) and tetraethoxysilane (SiOEth4). Apart from computational convenience, those two molecules were chosen for their great importance as precursors in the synthesis of porous silica materials such as zeolites and periodic mesoporous silica. As a first attempt, we transferred the parameters for the Si atom directly from the previous optimization on alkylsilanes, described above. However, we found that by using those parameters for silicon, it was not possible to simultaneously fit the four target experimental properties to within a reasonable tolerance (see open symbols in Figure 4). We carried out several tests by slightly perturbing some of the bonded potential parameters (e.g., bond lengths, dihedral constants) and the point charges (e.g., using CHelpG instead of DDEC charges),^117^ but found that the behavior was the same—the models still fell within the same region depicted by open symbols in Figure 4.

To solve this problem, we had to scale down the value of σ for the Si atom in alkoxysilanes, relative to the original value in alkylsilanes. A similar approach was used in the parametrization of both TraPPE^123^ and PolCA^49^ force fields for alcohols, where the value of σ for α-carbon atoms in secondary and tertiary alcohols was lower than the corresponding values in alkanes. It is justified physically by the stronger electron-withdrawing character of an oxygen atom when compared with a carbon atom. To account for this effect in a physically reasonable and systematic way, we reduced the value of σ for silicon by 5% for each oxygen-containing substituent group—e.g., the value of σ was scaled by 20% for tetramethoxysilane, which has 4 alkoxy groups, but only by 5% for methoxytrimethylsilane, which has a single alkoxy substituent. Using this scaling rule, our parametrization grid (σ ∈ [0.23;0.24;0.25;0.26;0.27;0.28] and ε ∈ [0.7;0.8;0.9;1.0;1.1;1.3;1.5]) yielded values for the target properties that passed close to the origin in Figure 4, indicating the possibility of obtaining a good set of parameters for the alkoxy oxygen. The values found after optimization were σ = 0.235 nm and ε = 1.344 kJ/mol.

For siloxane oxygen atoms, O_B_, we opted to directly transfer the parameters obtained for alkoxysilane oxygens, O_C_. This was because preliminary tests on hexamethyldisiloxane showed good performance with this set of parameters (see section 3). Finally, we carried out a parametrization of the LJ parameters of silanol oxygens by matching the density and enthalpy of vaporization of trimethylsilanol and triethylsilanol—two of the few silanols that are liquid at room temperature and for which experimental data was available. The value of σ for the Si atom was scaled using the same rule determined above for alkoxysilanes. A grid composed of σ ∈ [0.29;0.30;0.305;0.31;0.32;0.33] and ε ∈ [0.7;0.8;0.9;1.0;1.1;1.3;1.5;1.7] was used. The final set of LJ parameters for the organosilicate molecules considered in this paper is provided in Table 6.

**Table 6:** Final Lennard-Jones Parameters for the Organosilicate Molecules Considered in This Work^a^

atom	σ (nm)	ε (kJ/mol)
Si^0^	0.580	0.108
Si^1^	0.551	0.108
Si^2^	0.522	0.108
Si^3^	0.493	0.108
Si^4^	0.464	0.108
O_C_	0.235	1.344
O_B_	0.235	1.344
O_H_	0.304	1.750

a The superscript in the Si atom denotes the number of oxygen-containing substituent groups.

## 3 Results and Discussion

### 3.1 Alkylsilanes

**Figure 5:**
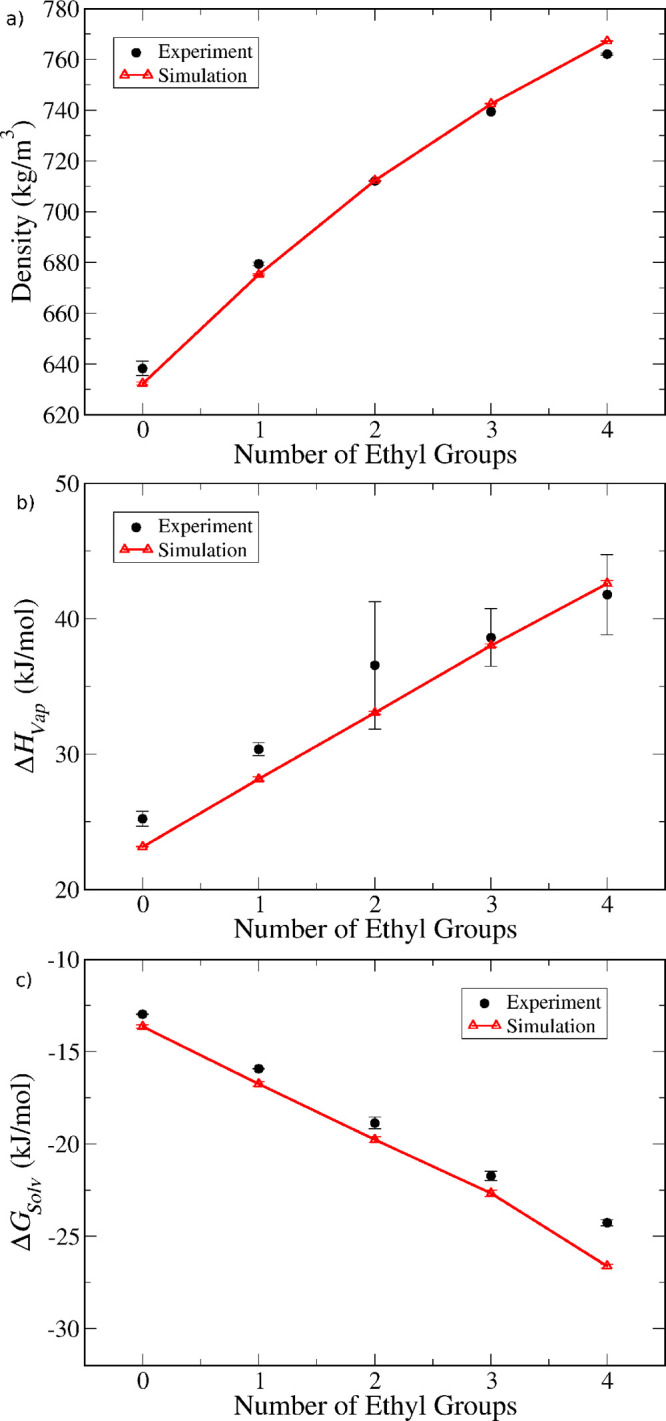
Comparison between model predictions (open triangles and lines) and experimental data (full circles) for (a) density, (b) enthalpy of vaporization, and (c) self-solvation free energy. The data is for alkylsilanes with only methyl or ethyl substituents, plotted as a function of the number of ethyl substituents.

As described in section 2.4, the LJ parameters for the silicon atom in tetrahedrally substituted alkylsilanes were optimized to match the density and enthalpy of vaporization of tetramethylsilane and tetraethylsilane. In Figure 5, we compare the model predictions against experimental data for those two properties, as well as for the self-solvation free energy (calculated from the experimental vapor pressure, as described in section 2.1) for all alkylsilanes containing methyl or ethyl substituent groups. The data is plotted as a function of the number of ethyl substituents for ease of visualization, hence in the order: tetramethylsilane, ethyltrimethylsilane, diethyldimethylsilane, triethylmethylsilane, and tetraethylsilane. As we can see from Figure 5a, the density of all compounds is predicted quite accurately. The simulated enthalpy of vaporization of most compounds is within experimental uncertainty, although there seems to be a tendency to slightly underestimate the enthalpy for methyl-rich compounds. In contrast, the self-solvation free energies show a slight underestimation—i.e., the simulations predict more favorable solvation for all compounds. However, with the exception of tetraethylsilane, the deviation is always below 1 kJ/mol, which is quite reasonable considering the simplifications of the model and the limited amount of experimental data. Furthermore, the model is able to capture the correct trends of increasing density, increasing enthalpy and decreasing solvation free energy with increasing number of ethyl groups.

It is useful to compare the predictions of our PolCA model to those of previous parametrization attempts. As discussed in the Introduction, very few models have been tested for liquid phase properties of alkylsilanes. A notable exception is the work of Polyakov et al.,^24^ who report values for the density and enthalpy of vaporization of tetraethylsilane using the model of Striolo et al.^20−22^ as well as their own reparametrized model. Their results are compared to the PolCA predictions and to experimental data in Table 7. It is clear from this table that PolCA performs even better than the model of Polyakov et al., even though the latter was specifically designed for the tetraethylsilane molecule.

**Table 7:** Density and Enthalpy of Vaporization of Tetraethylsilane Predicted by Several Models and Obtained from Experimental Measurements

model	source	ρ (kg/m^3^)	Δ*H*_Vap_ (kJ/mol)
Striolo et al.	ref (24)	795	33.2
Polyakov et al.	ref (24)	773	37.9
PolCA	This work	767.2	42.6
Experimental	several^a^	762.1	41.8

a See Supporting Information for details.

We also compared our model predictions against available experimental data for the self-diffusion coefficient and dielectric constant of alkylsilanes. For the diffusion coefficient, only data for tetramethylsilane was available. The values range between 3.6 × 10^–9^ and 4.4 × 10^–9^ m^2^/s, with an average value of 4.0 × 10^–9^ m^2^/s.^63,65,125−129^ After correcting for finite-size effects (see Figure S47), our predicted result of 3.8 × 10^–9^ m^2^/s compares quite well with the experimental measurements. As for the dielectric constant, experimental values for tetramethysilane (1.921) and tetraethylsilane (2.09) are available.^84^ The model predictions, after applying polarization corrections using eq 8, are 1.88 and 2.06, respectively, which are very close to the experimental values. It should be noted that before applying eq 8, the dielectric constants predicted by the simulations were all very close to 1 as a consequence of the nonpolar nature of alkylsilanes. As demonstrated previously for other classes of compounds,^46,47,49^ applying polarization corrections to the dielectric constant is essential to obtain predictions in line with experimental data.

### 3.2 Alkoxylsilanes

**Figure 6:**
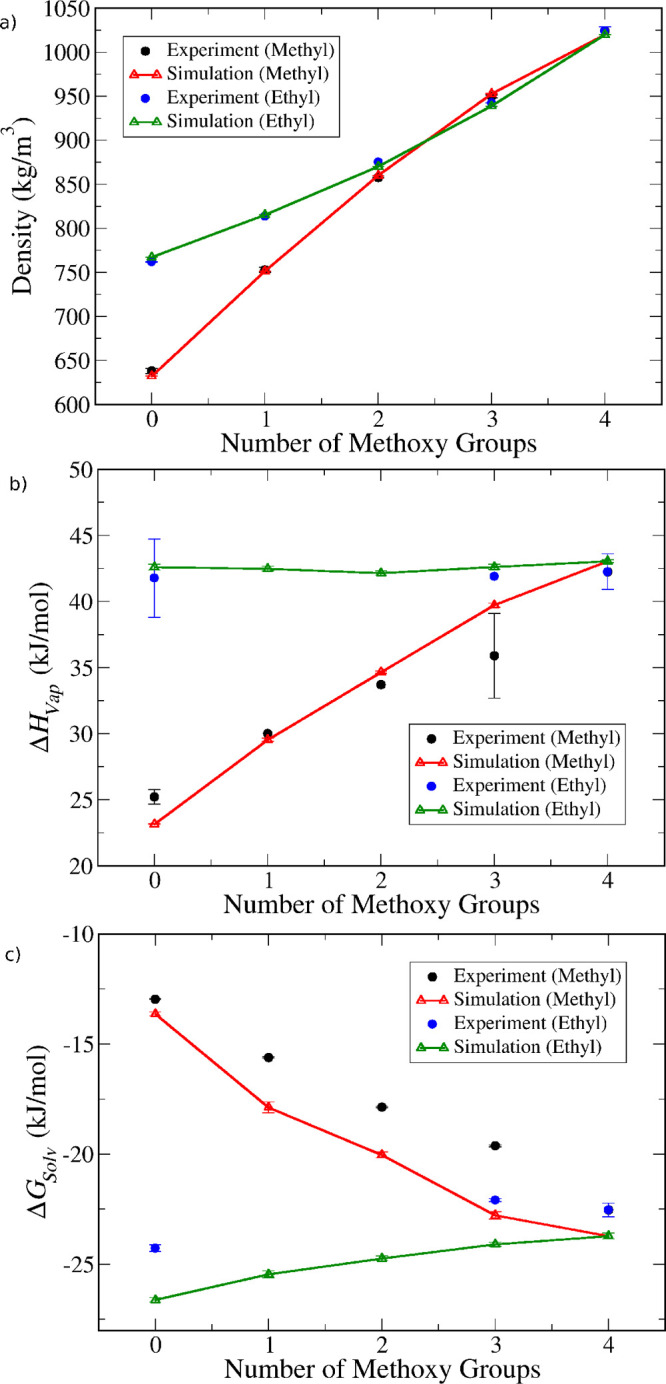
Comparison between model predictions (open triangles and lines) and experimental data (full circles) for (a) density, (b) enthalpy of vaporization, and (c) self-solvation free energy. The data is for alkylmethoxysilanes with either methyl (black/red) or ethyl (blue/green) substituents, plotted as a function of the number of methoxy groups present in the molecule.

LJ parameters for the oxygen atom in alkoxysilanes were optimized to match the target properties of tetramethoxysilane and tetraethoxysilane. To validate the model, we attempted to predict the density, enthalpy of vaporization, and self-solvation free energy of a wide range of compounds based on different combinations of methyl, ethyl, methoxy, and ethoxy substituents. For ease of visualization, we grouped these compounds into homologous series, as shown in Figure 6. Here, data for all molecules containing methoxy substituents is plotted (an equivalent plot for molecules with ethoxy substituents is shown in Figure S49). Two series are shown, one for methyl- and another for ethyl-containing molecules. Hence, the black circles (experimental) and red triangles (simulations) go from tetramethylsilane to tetramethoxysilane by progressively replacing methyl with methoxy groups, while the blue circles and green triangles show a similar progression but starting from tetraethylsilane and replacing ethyl groups with methoxy groups.

It can be seen that replacing either methyl or ethyl groups with methoxy groups causes a gradual increase in the bulk liquid density (Figure 6a). The model captures this trend quite well and predicts the density of all compounds quite accurately. The enthalpy of vaporization (Figure 6b), on the other hand, shows a different trend depending on the type of alkyl substituent—replacing methyl groups with methoxy groups causes a pronounced increase in the enthalpy, while replacing ethyl with methoxy groups practically causes no change. A similar tendency is observed for alkylethoxysilanes (Figure S49), although not as pronounced. The model is able to capture the trends quite reliably and predicts enthalpies that are in very good agreement with experimental data. In this regard, it is important to notice that a few experimental points have very large error bars, originating from discrepancies in different enthalpy measurements. In those cases, it would be useful to carry out additional measurements to resolve such discrepancies. Finally, Figure 6c shows that the self-solvation free energy is once again systematically underestimated by the model (i.e., simulations predict more favorable solvation), albeit by relatively small amounts.

**Figure 7:**
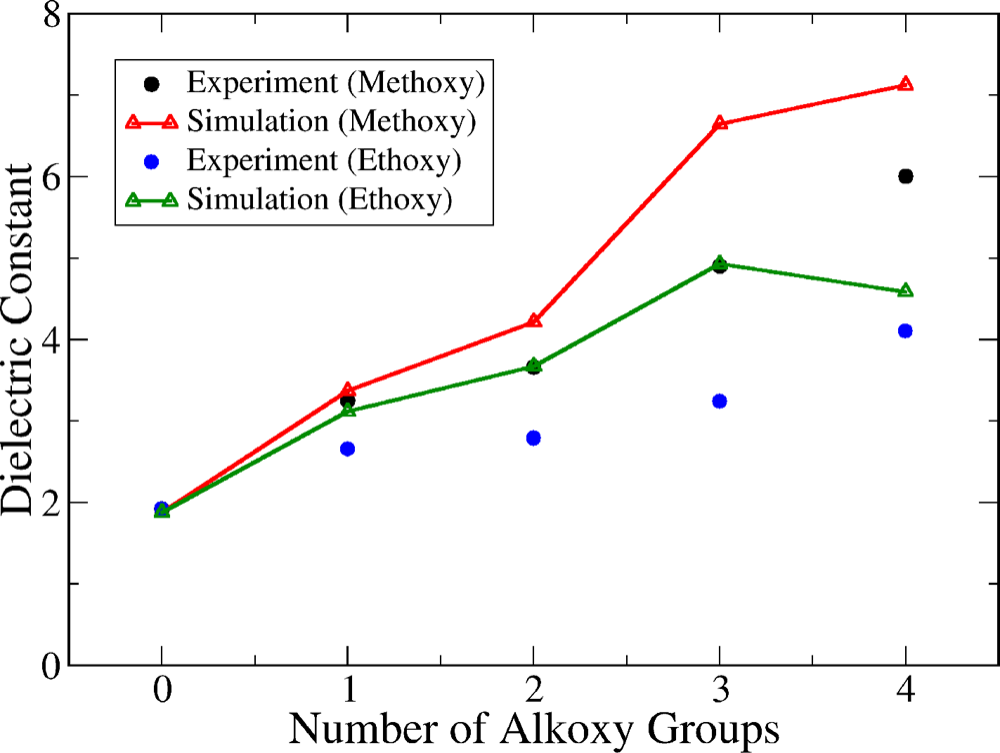
Comparison between model predictions (open triangles and lines) and experimental data (full circles) for the dielectric constant of methylalkoxysilanes with either methoxy (black/red) or ethoxy (blue/green) substituents, plotted as a function of the number of alkoxy groups present in the molecule.

As mentioned above, no data for the self-diffusion coefficient of alkoxysilanes was found. Dielectric constant data was only available for molecules with methyl substituents, and these are shown in Figure 7 together with the model predictions. The dielectric constant is generally higher for molecules with methoxy groups than their ethoxy counterparts and increases with the number of alkoxy substituents. This is to be expected, since the presence of alkoxy substituents increases the polarity of the molecule and hence its dielectric response. The model is able to capture all these trends correctly and yield quite good quantitative predictions across the whole family of compounds, although it does seem to slightly overestimate the magnitude of ε. As observed above for alkylsilanes, application of eq 8 is essential to obtain predictions in reasonable agreement with experiments.

Finally, in Table 8 we compare predictions from our model with those reported by Pereira et al. using their bespoke force field for alkoxysilanes as well as with experimental values. The new PolCA model yields much more accurate predictions of both properties for both compounds. Unfortunately, we are not aware of any additional simulation data for liquid alkoxysilanes that can be compared to our predictions.

**Table 8:** Density and Enthalpy of Vaporization of Tetramethoxysilane and Tetraethoxylsilane Predicted by Different Models and Obtained from Experimental Measurements

		SiOMet4		SiOEth4	
model	source	ρ (kg/m^3^)	Δ*H*_Vap_ (kJ/mol)	ρ (kg/m^3^)	Δ*H*_Vap_ (kJ/mol)
Pereira et al.	ref (25)	1040	66.2	941	64.3
PolCA	This work	1019.9	43.0	925.7	54.3
Experimental	several^a^	1024.2	42.3	926.6	52.5

a See Supporting Information for details.

### 3.3 Silanols

**Figure 8:**
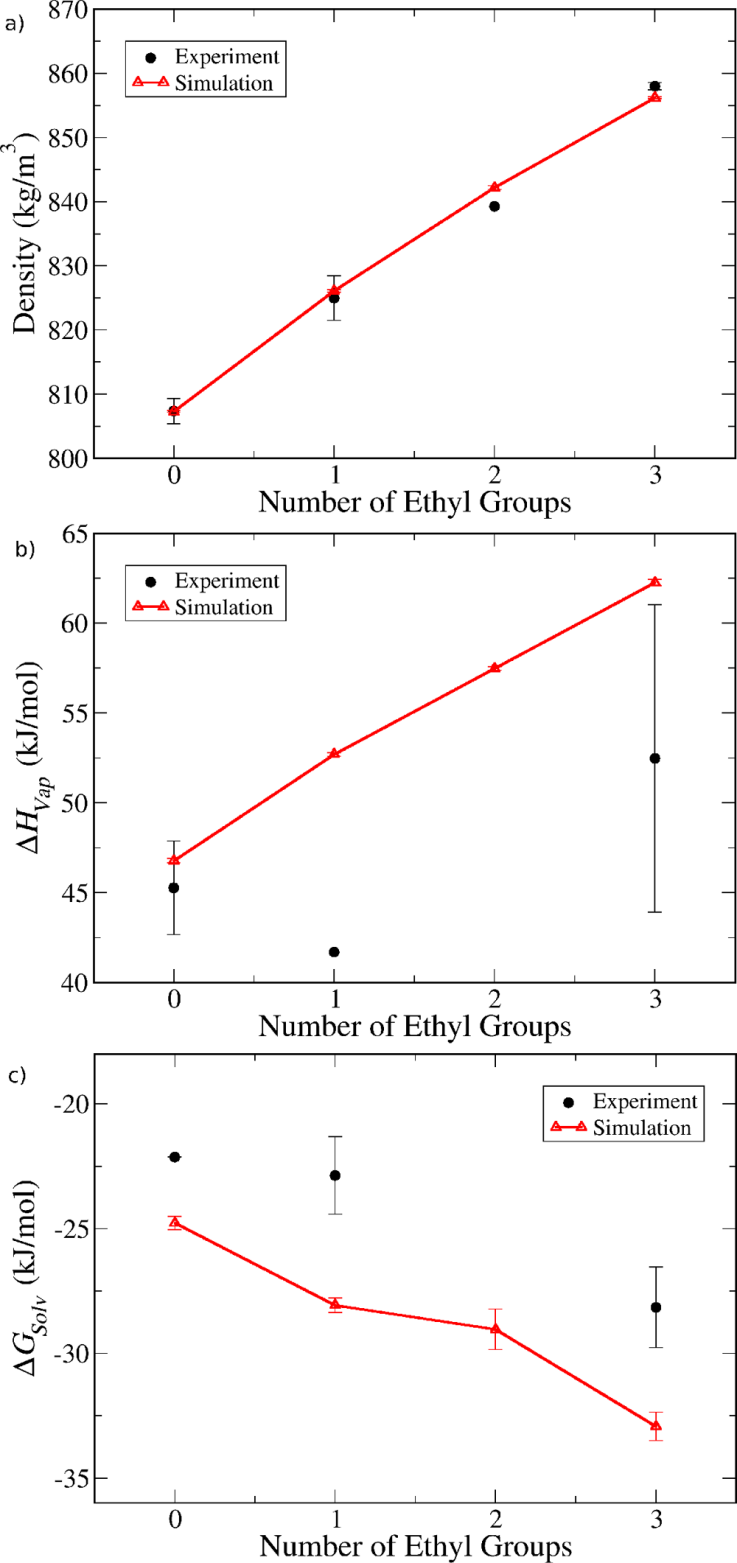
Comparison between model predictions (open triangles and lines) and experimental data (full circles) for (a) density, (b) enthalpy of vaporization, and (c) self-solvation free energy. The data is for alkylsilanols with only methyl or ethyl substituents, plotted as a function of the number of ethyl substituents.

In Figure 8, we show predictions for bulk liquid properties of silanol molecules. Unfortunately, molecules with more than one silanol group per Si atom are solid at room temperature, and therefore no experimental liquid phase properties were found. Therefore, we restrict our analysis here to monosilanol compounds with a range of alkyl substituents. Nevertheless, it is important to note that our model is, in principle, fully transferrable to molecules containing any number of Si–OH groups, including monosilicic acid, which plays a crucial role in the synthesis of silica-based materials. We intend to report simulations of such systems in future work.

Figure 8a shows that the model can match the experimental density values quite closely, replicating the trend of increasing density with number of ethyl substituents (replacing methyl groups). For the self-solvation free energy (Figure 8c), we observe again a systematic underestimation by the model, which is somewhat more pronounced than for alkoxysilanes. Nevertheless, the decreasing trend is reproduced quite faithfully. The situation with the enthalpy of vaporization, however, is less clear, primarily due to the small amount of data and the extremely high uncertainty associated with some points. In particular, for dimethylethylsilanol, only one measurement was available; hence, no uncertainty could be estimated. However, this value is unlikely to be very accurate, since an increase in the enthalpy is expected upon replacing a methyl with an ethyl group (as observed in Figures 5b and 6b for other classes of molecule). For triethylsilanol, three values were found, but these are very inconsistent (see Table S37), leading to very large error bars. Our simulation predictions are consistent with the value of 60.5 kJ/mol obtained from vapor pressure reported by Bažant et al.,^55^ but not with the two other experimental values. Further experimental measurements are required to assess the validity of our predictions.

We were also able to find experimental data for the dielectric constant of two silanol molecules, trimethylsilanol and triethylsilanol. Our model predicts the dielectric constant of the latter quite accurately (2.44 compared to 2.66 in experiment) and only slightly underestimates that of the former (5.84 compared to 7.17 in experiment). Once again, polarization corrections are essential to achieve this level of agreement, and this is particularly important for polar molecules—the uncorrected values are 1.39 and 2.85 for triethylsilanol and trimethylsilanol, respectively, which are far below the experimental values.

### 3.4 Overall Model Performance

**Figure 9:**
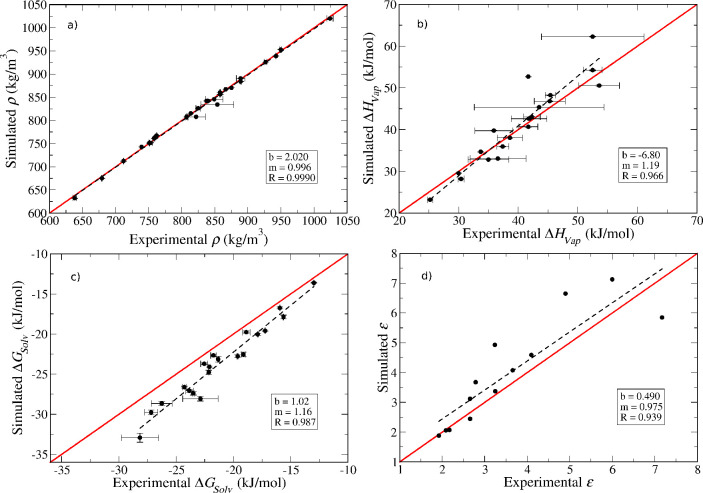
Overall comparison between simulation and experimental data for (a) density, (b) enthalpy of vaporization, (c) self-solvation free energy, and (d) dielectric constant. Points with error bars are shown for every compound for which experimental values are available. The red line represents parity between simulation and experiment, while the black dashed lines represent linear fits to the data with corresponding intercepts (*b*), slopes (*m*), and correlation coefficients (*R*) reported in the insets.

In Figure 9, we show an overall comparison between simulations and experiments for all compounds that have available experimental data. This includes some compounds which were not explicitly discussed in sections 3.1–3.3 because they did not belong to homologous series. In particular, we include hexamethyldisiloxane, which is the only compound that contains a bridging siloxane oxygen atom. As mentioned in section 2.4, directly transferring the parameters for the alkoxy oxygen to the siloxane oxygen led to results in very good agreement with experimental data—ρ = 761.7 ± 0.3 kg/m^3^ compared to 758.4 ± 0.7 kg/m^3^; Δ*H*_Vap_ = 35.9 ± 0.1 kJ/mol compared to 37.4 ± 1 kJ/mol; Δ*G*_Solv_ = −22.5 ± 0.3 kJ/mol compared to −19.1 ± 0.2 kJ/mol; ε = 2.07 compared to 2.17. Full tables containing all the simulated data, as well as experimental data, when available for each property, are provided in Supporting Information (Tables S73–S77).

We can see from Figure 9a that the density is very accurately predicted for all relevant compounds. Agreement for the enthalpy of vaporization is also quite satisfactory (Figure 9b), particularly taking into account the very large uncertainty associated with the experimental values for some organosilicate compounds. Additional measurements of this property would be extremely useful to provide more robust validation data. As for the self-solvation free energy (Figure 9c), the simulations predict systematically more favorable solvation than observed experimentally, although the differences are seldom larger than 2–3 kJ/mol. Given that the enthalpy of vaporization shows no such systematic deviation, the observed trend in the free energies is likely to reflect an overestimation of the entropy of solvation by the model, although a more detailed analysis is needed to confirm this. Arguably, one could include solvation free energies for a few compounds in the parametrization data set to improve the model’s performance for predicting this property. However, this would be quite computationally expensive, and the additional effort was not deemed worthwhile. Finally, Figure 9d shows that, despite a fair amount of scatter, the model does quite a reasonable job at predicting the dielectric constant. It is important to note that this property was not considered in the model parametrization, and therefore the results in Figure 9d constitute pure predictions. The good agreement observed is further evidence that polarization corrections are important in predicting the dielectric constant, as shown for other families of liquids.^46,47^

## 4 Conclusions

In this paper, we reported the parametrization of a new model for organosilicate molecules in the liquid phase. The model is based on the United-Atom approach and is an extension of the Polarization-Consistent Approach that was previously developed for alkanes^48^ and alkyl alcohols.^49^ PolCA represents a new paradigm in force field development, whereby polarization effects are explicitly considered in the calculation of phase-change and electronic properties through the application of *post facto* corrections. To parametrize and validate the model, we carried out a comprehensive data collection and analysis of experimental properties of organosilicates, obtaining robust data with realistic uncertainty estimates. This allowed us to fit the model parameters in a step-by-step approach and carry out a thorough model validation. The experimental database is itself an important outcome of the present study, as it enables other researchers to carry out their own model development and validation for this important class of molecules.

The model was shown to accurately predict the density and enthalpy of vaporization of several molecules, including alkylsilanes, alkoxysilanes, siloxanes, and silanols, even those that were not used in its parametrization. Furthermore, the model provided reasonable predictions of the self-solvation free energy of organosilicates, despite a small systematic deviation. Predictions of the dielectric constant were quite good, provided that polarization effects were taken into account—if not, this property was severely underestimated, as shown previously.^46,47^ For the only molecule for which experimental self-diffusion data was available, namely, tetramethylsilane, the agreement between simulation and experiment was very good. This bodes well for the transferability of the model to other organosilicates containing the same functional groups. The next step in this parametrization will be to extend the approach to include organosilicates with less than four substituent groups (i.e., containing SiH_*x*_ groups), as well as those with halogen substituents, which are widely used in the synthesis of polymer materials. We hope to report on such developments in forthcoming publications.

## Supporting Information Available

Details of the collection and analysis of experimental data for organosilicates, including full data tables; details of the model parametrization, including point charge calculations, bond and angle parameters, dihedral angle fits; additional model validation results (PDF)

## Notes

All data underpinning this publication are openly available from the University of Strathclyde KnowledgeBase at 10.15129/0db53861-cc47-4617-81a5-88ce1a23349a.
