# Correction to “New Force-Field for Organosilicon Molecules in the Liquid Phase”

**DOI:** 10.1021/acsphyschemau.2c00004

**Published:** 2022-02-07

**Authors:** Miguel Jorge, Tom Stavert, Andrew W. Milne, Maria Cecilia Barrera, José R. B. Gomes

We noticed a mistake in the values of some dihedral parameters reported in Table 5 of our original paper. Incorrect parameters were reported for the O_C_SiO_B_Si dihedral (and hence also for the O_B_SiO_B_Si dihedral, which was assumed to be identical) and for the O_H_SiO_B_Si dihedral. Table 5 here reports the correct parameters and should replace Table 5 in the original paper.

**Table 5:** Final Set of Torsion Parameters (in kJ/mol) for All Dihedrals Considered in This Work

dihedral	*k*_T,0_	*k*_T,1_	*k*_T,2_	*k*_T,3_	*k*_T,4_	*k*_T**,**5_
CCSiC	1.224	3.672	0.0	–4.895	0.0	0.0
CSiO_C_C	1.364	4.093	0.0	–5.457	0.0	0.0
CCSiO_C_	0.692	2.456	0.437	–3.416	0.0	0.0
CCO_C_Si	7.949	7.892	2.723	–18.563	0.0	0.0
CO_C_SiO_C_	4.314	4.803	0.0	–0.489	0.0	0.0
CSiO_H_H	0.870	2.600	0.0	–3.470	0.0	0.0
CCSiO_H_	0.801	2.760	0.508	–3.615	0.0	0.0
O_C_SiO_H_H	10.189	2.939	0.0	6.918	0.0	0.0
CO_C_SiO_H_	13.021	0.350	–39.801	–25.132	39.605	31.769
O_H_SiO_H_H	10.071	6.167	2.322	6.236	0.0	0.0
CCSiO_B_	0.692	2.456	0.437	–3.416	0.0	0.0
CO_C_SiO_B_	4.314	4.803	0.0	–0.489	0.0	0.0
CSiO_B_Si	0.0503	0.151	0.0	–0.201	0.0	0.0
O_C_SiO_B_Si	7.984	8.311	0.0	–0.327	0.0	0.0
O_B_SiO_B_Si	7.984	8.311	0.0	–0.327	0.0	0.0
O_H_SiO_B_Si	13.239	13.076	0.0	0.163	0.0	0.0

Tom Stavert has been added a coauthor. His author affiliation address is as follows:

Department of Chemical and Process Engineering, University of Strathclyde, 75 Montrose Street, Glasgow G1 1XJ, United Kingdom

