# Supplementary material for: New Force-Field for Organosilicon Molecules in the Liquid Phase
Source: ACS Phys Chem Au. 2021 Aug 27;1(1):54–69. doi: 10.1021/acsphyschemau.1c00014 (PMC8679648; doi:10.1021/acsphyschemau.1c00014)
Supplement: Supplementary file 1 — pg1c00014_si_001.pdf [file pg1c00014_si_001.pdf]

Supplementary Material for:

New Force-Field for Organosilicon Molecules in the Liquid Phase

Miguel Jorge[1,], Andrew W. Milne[1], Maria Cecilia Barrera[1], José R. B. Gomes[2]*

[1] Department of Chemical and Process Engineering, University of Strathclyde, 75 Montrose Street, Glasgow G1 1XJ, United Kingdom

[2] CICECO – Aveiro Institute of Materials, Department of Chemistry, University of Aveiro, Campus Universitário de Santiago, Aveiro, Portugal

[*] Email: miguel.jorge@strath.ac.uk

S1. Experimental Data

Ideally, one would like to be able to find experimental data for each target property of each compound at the reference temperature from multiple sources, so as to increase confidence in the value of that property when comparing with simulation data. Unfortunately, experimental data for thermodynamic and transport properties of organosilicon compounds is rather scarce. As such, a significant effort of data collection and curation was needed, which is described in detail below.

For each property, we present all the experimental data collected and analysed for each compound, reporting average values and uncertainty estimates that were used in the force field development and validation stages. The average was taken over all data points after appropriate curation, and the uncertainty is reported as twice the standard error of the mean (i.e. corresponding to approximately a 95% confidence interval). We refer to each compound by name, chemical formula and acronym used in the main paper.

S1.1 Density

Whenever the density values were reported at temperatures other than 298 K, they were corrected as follows:

1) For each literature source where data for density at several temperatures was available, this was fitted linearly within a range of temperatures around 293-298 K (if available). The density at 298 K was obtained from this fit by interpolation (or extrapolation, in the small number of cases where 298 K was marginally outside the range of available data).

2) The slope of the fits carried out in 1) allowed us to estimate a correction term in kg/m^3K, which was applied to correct cases where only a single density value at a temperature other than 298 K was reported.

3) If data for density as a function of temperature was available for the same compound from multiple sources, separate fits were carried out for each data set as in 1). The corrections were then averaged over all fits, and the average correction in $kg \cdot m^{-3} \cdot K^{-1}$ was applied as in 2) to convert all densities for that compound to a temperature of 298 K.

4) If data for density as a function of temperature was not available for a given compound, the correction was estimated from data for chemically similar compounds (see details below).

Tetramethylsilane; $C_4H_{12}Si$; Met4Si

Density data at 298 K and 1 bar was available from Sharko et al. [1] and Yaws [2]. Density data as a function of temperature was obtained from Yokoyama et al. [3] and fitted linearly, as shown in Figure S1.

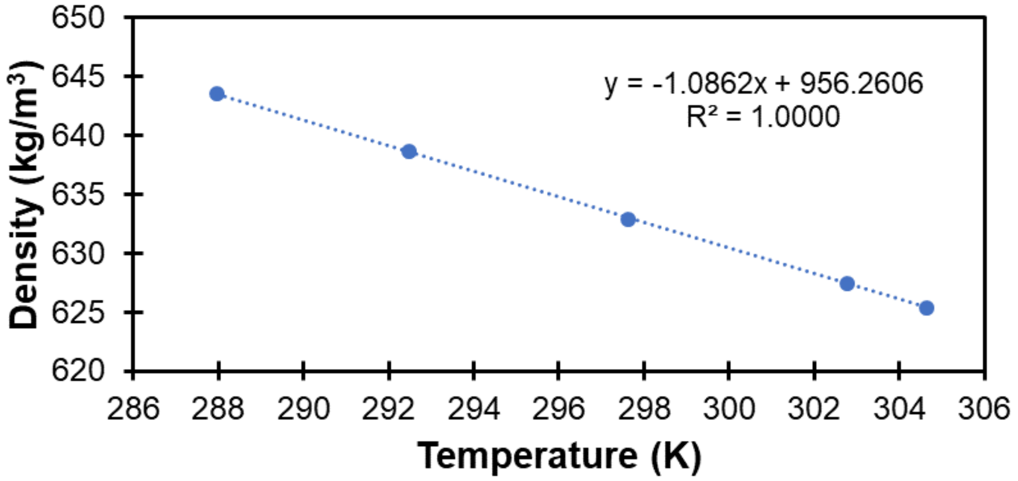

Figure S1 *– Linear fit to the data of Yokoyama et al. [3] for density of tetramethylsilane as a function of temperature in the vicinity of 293-298 K. The linear fit equation and correlation coefficient are show in the inset.*

The slope of the fit gives the density correction, which was rounded to -1.1 kg·m^{-3}·K^{-1}. This was used to correct density values that were reported at temperatures different from 298 K. Namely, the data from Bažant et al. [4] and Rochow [5], reported at 293 K, were thus corrected to 298 K. Finally, data was available from Parkhurst and Jonas [6] at elevated pressures; in this case, the density was extrapolated to 1 bar using the coefficients for the Tait equation determined by the authors.

Table S1 *– Experimental data for density of Met4Si, together with the average value and uncertainty.*

**Density at 298 K (kg/m³)**	**Reference**	**Comments**
640.0	[1]	
640.9	[2]	
632.4	[3]	Interpolated in the range 288-305 K
640.5	[4]	Corrected from 293 K using data from [3]
635.6	[5]	Corrected from 293 K using data from [3]
640.2	[6]	Estimated at 1 bar from Tait equation [6]
**638.3 ± 2.8**		

Tetraethylsilane; $C_8H_{20}Si$; Eth4Si

Density data at 298 K was available from Yaws [2], Polyakov et al. [7], and Iseard et al. [8]. Density data as a function of temperature was available from four different sources: Yokoyama et al. [3], Bažant et al. [4], Sugden and Wilkins [9], and Whitmore et al. [10]. The four data sets, with corresponding linear fits, are shown in Figure S2.

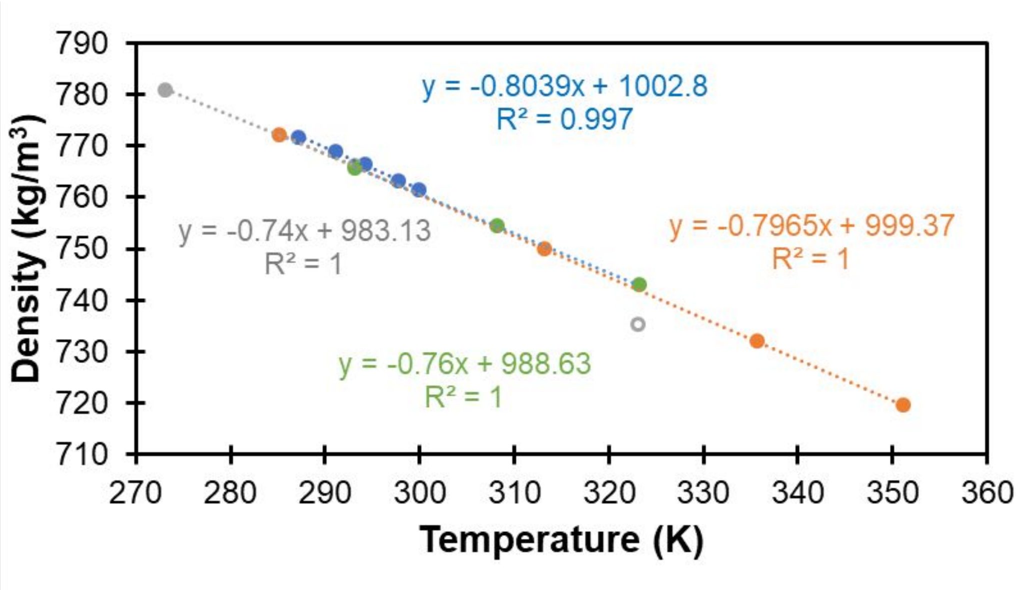

Figure S2 *– Linear fits to the data for density of tetraethylsilane as a function of temperature obtained from: Yokoyama et al. [3] (blue), Bažant et al. [4] (green), Sugden and Wilkins [9] (orange), and Whitmore et al. [10] (grey). The linear fit equations and correlation coefficients are show in the insets with the corresponding color code. Notice that the last point at 323 K from Whitmore et al. (open circle) was excluded from the fit because it falls outside the observed trends.*

As we can see, all the data are quite consistent with the exception of the last point from Whitmore et al. [10], which was therefore removed from the analysis. Each fit equation was then used to estimate the density at 298 K for each of the corresponding data sets.

The density correction term was calculated from the slopes of the fits to the data of Yokoyama et al. [3], Bažant et al. [4], and Sugden and Wilkins [9] (the data from Whitmore et al. [10] was not considered because only two points were used in the fit), from which we obtained an average value of -0.79 kg·m^{-3}·K^{-1}. This was used to correct density values that were reported at temperatures different from 298 K. Namely, the data from Rochow [5] and Eaborn [11], reported at 293 K, were thus corrected to 298 K.

Table S2 *– Experimental data for density of Eth4Si, together with the average value and uncertainty.*

Density at 298 K (kg/m^3)	Reference	Comments
761.9	[2]	
762.0	[8]	
761.0	[7]	
763.1	[3]	Interpolated
762.0	[4]	Interpolated
761.9	[9]	Interpolated
762.5	[10]	Interpolated
762.1	[5]	Corrected from 293 K using data from [3, 4, 9]
762.2	[11]	Corrected from 293 K using data from [3, 4, 9]
**761.1 ± 0.4**		

<u>Methyltriethylsilane; $C_7H_{18}Si$; M1E3Si</u>

No data was found for the density at 298 K. However, density data as a function of temperature was obtained from Whitmore et al. [10] and fitted linearly, as shown in Figure S3.

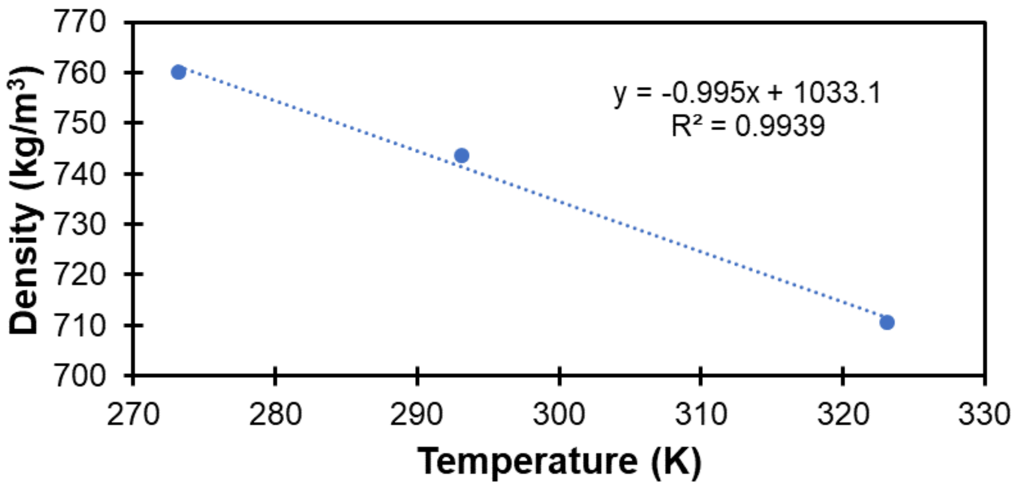

Figure S3 *– Linear fit to the data of Whitmore et al. [10] for density of methyltriethylsilane as a function of temperature. The linear fit equation and correlation coefficient are show in the inset.*

As can be seen, the data deviate slightly from linearity, as observed in Figure S2 for tetraethylsilane (see points in grey). Because no other data for the temperature dependence of density was available for this compound or any other alkylsilanes, a different approach was adopted. Starting from the temperature correction determined above for tetramethylsilane and tetraethylsilane, we estimated the corrections for intermediate molecules (i.e. containing combinations of methyl and ethyl substituent groups) by interpolation. The results for these corrections are shown in Table S3.

Table S3 *– Estimated density corrections for the effect of temperature on alkylsilanes.*

Compound	Number of ethyl substituents	Density Correction ($kg \cdot m^{-3} \cdot K^{-1}$)
Met4Si	0	-1.10
M3E1Si	1	-1.02
M2E2Si	2	-0.95
M1E3Si	3	-0.87
Eth4Si	4	-0.79

It is useful to note that similar values for the correction could be obtained from the expression of the temperature dependence of density reported by Yaws [12]. In that compilation, coefficients are reported for both Met4Si and Eth4Si. By applying the expression over the range between 293 and 298 K, we obtained corrections of -1.2 $kg \cdot m^{-3} \cdot K^{-1}$ for Met4Si and -0.8 $kg \cdot m^{-3} \cdot K^{-1}$ for Eth4Si, which are indeed quite similar to the values reported in Table S3, supporting the robustness of our approach.

Making use of the values in Table S3, we were able to correct the data from Whitmore et al. at 293 K [10], as well as the data from Bažant et al. [4], also reported at 293 K. The corresponding corrected densities are shown in Table S4. Because these two values are identical within the reported precision, no uncertainty could be reliably estimated.

Table S4 *– Experimental data for density of M1E3Si, together with the average value.*

Density at 298 K (kg/m^3)	Reference	Comments
739.4	[4]	Corrected from 293 K using data from Table S3
739.4	[10]	Corrected from 293 K using data from Table S3
**739.4**		

Trimethylethylsilane; $C_5H_{14}Si$; M3E1Si

No data was found for the density at 298 K. Whitmore et al. [10] only report data at 273 and 293 K. Therefore, we again corrected the latter, as well as the value reported by Bažant et al. [4] at 293 K, using the estimated corrections from Table S3. The final results are shown in Table S5.

Table S5 *– Experimental data for density of M3E1Si, together with the average value and uncertainty.*

Density at 298 K (kg/m^3)	Reference	Comments
679.8	[4]	Corrected from 293 K using data from Table S3
679.1	[10]	Corrected from 293 K using data from Table S3
**679.5 ± 0.7**		

Dimethyldiethylsilane; $C_6H_{16}Si$; M2E2Si

Again, no data was found for the density at 298 K, as well as for the temperature dependence of density. We therefore applied the corrections shown in Table S3 to correct the value of Bažant et al. [4] at 293 K. We were also able to find a density value from a chemical compounds website [13], which was quite similar to the corrected value of Bažant et al. (see Table S6). This allows us to obtain an estimate for the uncertainty; however, given that only two very similar points were available, this is likely to be somewhat underestimated.

Table S6 *– Experimental data for density of M2E2Si, together with the average value and uncertainty.*

Density at 298 K (kg/m^3)	Reference	Comments
712.1	[4]	Corrected from 293 K using data from Table S3
712.0	[13]	Corrected from 293 K using data from Table S3
**712.1 ± 0.1**		

Tetraethoxylsilane; $C_8H_{20}SiO_4$; Si(OEth)4

There was one available data point for density at 298 K, from Kato and Tanaka [14] and there were three data sets that reported the temperature dependence of density, those of Bažant et al. [4], Sugden and Wilkins [9], and Yokoyama et al. [3]. Those data sets, together with the corresponding linear fits, are shown in Figure S4.

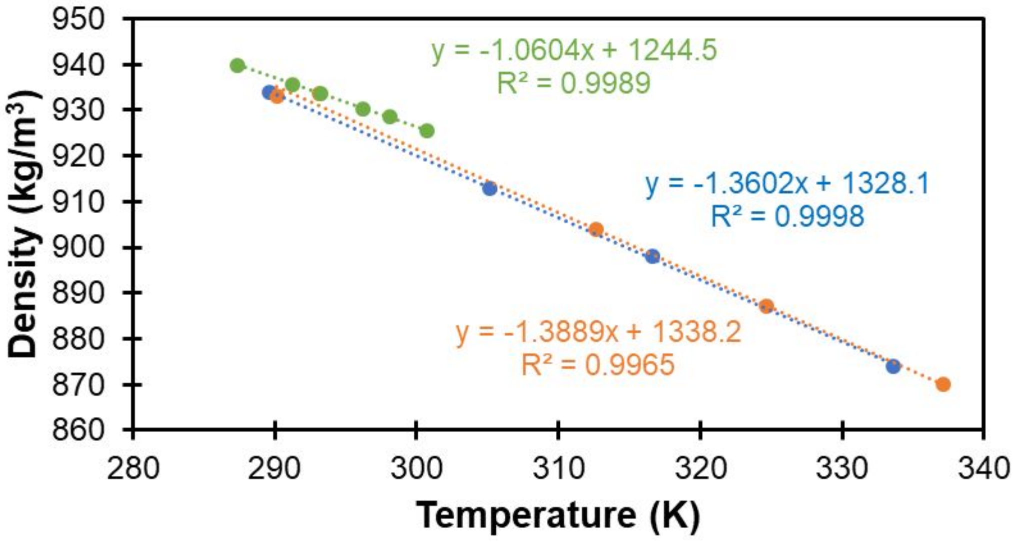

***Figure S4** – Linear fits to the data for density of tetraethoxysilane as a function of temperature obtained from: Bažant et al. [4] (orange), Sugden and Wilkins [9] (blue), and Yokoyama et al. [3] (green). The linear fit equations and correlation coefficients are show in the insets with the corresponding color code.*

Two of the data sets [4, 9] are practically overlapping, whereas the third [3] shows a different slope. The average slope from the three data sets was taken to estimate a temperature correction of -1.3 kg/m^{3}K for Si(OEth)4, which was applied to correct the values reported at 293 K by Yaws [2] and Rochow [5]. All the density values, together with their average and uncertainty estimate, are shown in Table S7.

***Table S7** – Experimental data for density of Si(OEth)4, together with the average value and uncertainty.*

Density at 298 K (kg/m^3)	Reference	Comments
924.1	[4]	Interpolated
922.6	[9]	Interpolated
928.3	[3]	Interpolated
929.0	[14]	
926.7	[5]	Corrected from 293 K using data from [3, 4, 9]
925.7	[2]	Corrected from 293 K using data from [3, 4, 9]
**926.1 ± 2.0**		

Dimethyldimethoxylsilane; $C_4H_{12}SiO_2$; M2Si(OM)2

The only data available for this compound at 298 K was that of Yaws [12], who also reported parameters for a correlation of density vs temperature over a wide range of conditions. We applied that correlation in the vicinity of 293-298 K to estimate a temperature correction of -1.3 kg·m^{-3}·K^{-1} for M2Si(OM)2. This is in perfect agreement with the value determined above for Si(OEth)4, and was thus applied to correct the values reported at 293 K by Eaborn [11] and Bažant et al. [4]. All the density values, together with their average and uncertainty estimate, are shown in Table S8.

Table S8 – Experimental data for density of M2Si(OM)2, together with the average value and uncertainty.

Density at 298 K (kg/m^3)	Reference	Comments
858.3	[12]	From correlation (temperature dependence)
858.1	[4]	Corrected from 293 K using data from [12]
857.3	[11]	Corrected from 293 K using data from [12]
**857.8 ± 0.6**		

Tetramethoxylsilane; $C_4H_{12}SiO_4$; Si(OMet)4

There was one available data point for density at 298 K, from Kato and Tanaka [14], and there were two data sets that reported the temperature dependence of density, those of Bažant et al. [4] and Sugden and Wilkins [9]. Those data sets, together with the corresponding linear fits, are shown in Figure S5.

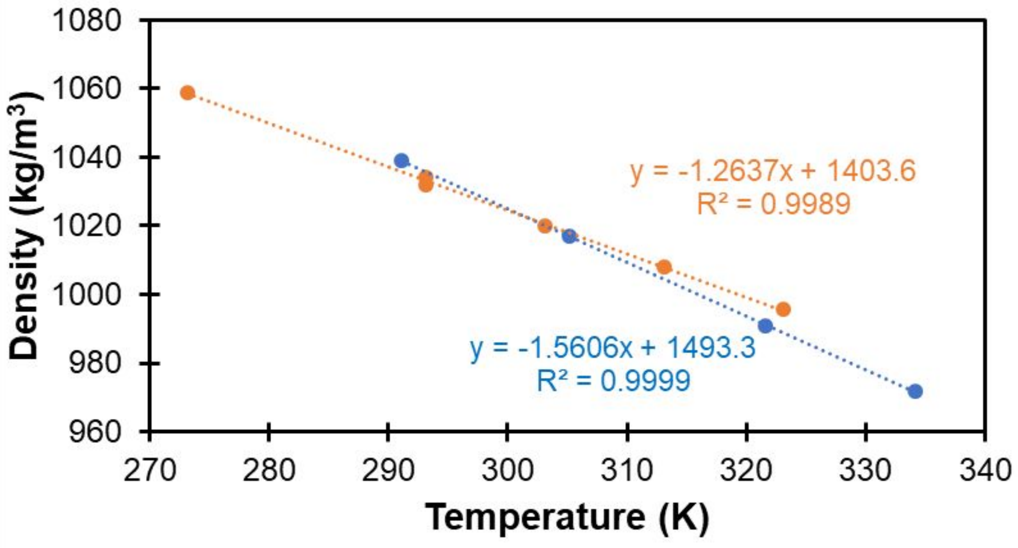

Figure S5 *– Linear fits to the data for density of tetramethoxysilane as a function of temperature obtained from: Bažant et al. [4] (orange), and Sugden and Wilkins [9] (blue). The linear fit equations and correlation coefficients are show in the insets with the corresponding color code.*

Although the density values around room temperature are quite similar in both data sets, the slopes of the fits are rather distinct, which points to a potential inconsistency between the two sets of measurements. We note that the slope obtained from fitting the data of Bažant et al. [4] agrees very well with the value of -1.3 kg·m^{-3}·K^{-1} obtained previously for both Si(OEth)4 and M2Si(OM)2, while that obtained from data of Sugden and Wilkins [9] is somewhat larger. For consistency, and because we were not able to find any more data for the temperature dependence of density of alkoxysilanes, we have applied a correction of -1.3 kg·m^{-3}·K^{-1} to all alkoxysilane compounds, including Si(OMet)4. This allowed us to correct the values reported by Rochow [5] (at 296 K) and Yaws [2] (at 293 K). The final density values, together with their average and uncertainty estimate, are shown in Table S9. While

the top three values are very similar, the value reported by Yaws [2] is significantly lower, which leads to a rather large uncertainty for this compound.

Table S9 *– Experimental data for density of Si(OMet)4, together with the average value and uncertainty.*

Density at 298 K (kg/m^3)	Reference	Comments
1026.8	[4]	Interpolated
1028.0	[9]	Interpolated
1021.0	[14]	
1028.4	[5]	Corrected from 296 K using data from [4, 12]
1016.8	[2]	Corrected from 293 K using data from [4, 12]
**1024.2 ± 4.6**		

Trimethylmethoxylsilane; $C_4H_{12}SiO$; M3SiOM

There were two values of density available at 298 K, from Yaws [2] and Bažant et al. [4]. The latter also provided a value at 293 K, while Eaborn [11] also reported the density at 293 K; both those values were corrected to 298 K as described above (see Table S10).

Table S10 *– Experimental data for density of M3SiOM, together with the average value and uncertainty.*

Density at 298 K (kg/m^3)	Reference	Comments
756.0	[2]	
753.7	[4]	
749.5	[4]	Corrected from 293 K using data from [12]
752.7	[11]	Corrected from 293 K using data from [12]
**753.0 ± 2.7**		

Trimethylethoxylsilane; $C_5H_{14}SiO$; M3SiOE

There was one value of density at 298 K, from Bažant et al. [4], and several values at 293 K: from Bažant et al. [4], Eaborn [11], Rochow [5], Yaws [2], and Sauer [15]. The latter were all corrected to 298 K as described previously (see Table S11).

Table S11 *– Experimental data for density of M3SiOE, together with the average value and uncertainty.*

Density at 298 K (kg/m^3)	Reference	Comments
752.1	[4]	
750.8	[4]	Corrected from 293 K using data from [12]
750.8	[2]	Corrected from 293 K using data from [12]
751.1	[11]	Corrected from 293 K using data from [12]
751.5	[5]	Corrected from 293 K using data from [12]
750.8	[15]	Corrected from 293 K using data from [12]
**751.2 ± 0.4**		

Triethylmethoxylsilane; $C_7H_{18}SiO$; E3SiOM

Only one density value was found at 293 K, reported by Bažant et al. [4]. This was corrected to 298 K as described above, yielding a density of **813.8 kg/m^3** for E3SiOM. No uncertainty could be estimated for this compound.

Triethylethoxylsilane; $C_8H_{20}SiO$; E3SiOE

There several density values available at 293 K, from Yaws [2], Eaborn [11], and Bažant et al. [4], as well as a value at 299.5 K from Bažant et al. [4]. All these values were corrected to 298 K as described above (see Table S12). We note there is significant disagreement between two families of values, leading to a very high uncertainty in the density for E3SiOE.

Table S12 *– Experimental data for density of E3SiOE, together with the average value and uncertainty.*

**Density at 298 K (kg/m^3)**	**Reference**	**Comments**
809.5	[2]	Corrected from 293 K using data from [12]
809.5	[4]	Corrected from 293 K using data from [12]
833.0	[4]	Corrected from 299.5 K using data from [12]
834.9	[11]	Corrected from 293 K using data from [12]
**822 ± 14**		

Methylethyldimethoxylsilane; $C_5H_{14}SiO_2$; MESi(OM)2

Only one density value was found at 293 K, reported by Bažant et al. [4]. This was corrected to 298 K as described above, yielding a density of **866.6 kg/m^3** for MESi(OM)2. No uncertainty could be estimated for this compound.

Diethyldimethoxylsilane; $C_6H_{16}SiO_2$; E2Si(OM)2

Only one density value was found at 293 K, reported by Bažant et al. [4]. This was corrected to 298 K as described above, yielding a density of **875.3 kg/m^3** for E2Si(OM)2. No uncertainty could be estimated for this compound.

Dimethyldiethoxylsilane; $C_6H_{16}SiO_2$; M2Si(OE)2

There was one value of density at 298 K, from Yaws [2], and three values at 293 K: from Bažant et al. [4], Eaborn [11], and Rochow [5]. The latter were all corrected to 298 K as described previously (see Table S13).

Table S13 *– Experimental data for density of M2Si(OE)2, together with the average value and uncertainty.*

**Density at 298 K (kg/m^3)**	**Reference**	**Comments**
865.0	[2]	
833.0	[4]	Corrected from 293 K using data from [12]
833.5	[11]	Corrected from 293 K using data from [12]
883.5	[5]	Corrected from 293 K using data from [12]
**854 ± 25**		

Once again, we note a significant discrepancy between values reported in different sources, leading to a very large uncertainty.

Methylethyldiethoxylsilane; $C_7H_{18}SiO_2$; MESi(OE)2

Only one density value was found at 293 K, reported by Bažant et al. [4]. This was corrected to 298 K as described above, yielding a density of **837.5 kg/m³** for MESi(OE)2. No uncertainty could be estimated for this compound.

Diethyldiethoxylsilane; $C_8H_{20}SiO_2$; E2Si(OE)2

Two density values were found, one at 293 K from Bažant et al. [4] and one at 273 K from Rochow [5]. Both were corrected to 298 K as described previously (see Table S14).

Table S14 *– Experimental data for density of E2Si(OE)2, together with the average value and uncertainty.*

Density at 298 K (kg/m³)	Reference	Comments
855.7	[4]	Corrected from 293 K using data from [12]
842.5	[5]	Corrected from 273 K using data from [12]
**849 ± 13**		

Methyltrimethoxylsilane; $C_4H_{12}SiO_3$; MSi(OM)3

We found two identical density values 298 K, from Eaborn [11] and Bažant et al. [4], and two other identical values at 293 K, from Yaws [2] and Bažant et al. [4]. The latter were corrected to 298 K as described above (see Table S15).

Table S15 *– Experimental data for density of MSi(OM)3, together with the average value and uncertainty.*

Density at 298 K (kg/m³)	Reference	Comments
951.0	[11]	
951.0	[4]	
948.3	[4]	Corrected from 293 K using data from [12]
948.3	[2]	Corrected from 293 K using data from [12]
**949.7 ± 1.6**		

Methyltriethoxylsilane; $C_7H_{18}SiO_3$; MSi(OE)3

Three values of density at 298 K were available, from Yaws [2], Shorr [16] and Zhang et al. [17], as well as one value at 293 K from Bažant et al. [4], and one at 273 K from Rochow [5]. Table S16 reports all values corrected to 298 K, together with their average and estimated uncertainty.

Table S16 – *Experimental data for density of MSi(OE)3, together with the average value and uncertainty.*

Density at 298 K (kg/m^3)	Reference	Comments
894.8	[2]	
877.0	[16]	
889.6	[17]	
888.3	[4]	Corrected from 293 K using data from [12]
895.5	[5]	Corrected from 273 K using data from [12]
**889 ± 7**		

Ethyltrimethoxylsilane; $C_5H_{14}SiO_3$; ESi(OM)3

Two density values at 293 K, from Yaws [2] and Bažant et al. [4], and one at 273 K from Rochow [5], were available. All values were corrected to 298 K as described above (see Table S17).

Table S17 – *Experimental data for density of ESi(OM)3, together with the average value and uncertainty.*

Density at 298 K (kg/m^3)	Reference	Comments
942.3	[2]	Corrected from 293 K using data from [12]
942.3	[4]	Corrected from 293 K using data from [12]
942.5	[5]	Corrected from 273 K using data from [12]
**942.4 ± 0.1**		

Ethyltriethoxylsilane; $C_8H_{20}SiO_3$; ESi(OE)3

We found two density values at 293 K, from Yaws [2] and Bažant et al. [4], and two values at 293 K, from Rochow [5] and Bažant et al. [4]. All values were corrected to 298 K as described above (see Table S18).

Table S18 – *Experimental data for density of ESi(OE)3, together with the average value and uncertainty.*

Density at 298 K (kg/m^3)	Reference	Comments
888.5	[5]	Corrected from 273 K using data from [12]
888.1	[4]	Corrected from 273 K using data from [12]
889.8	[4]	Corrected from 293 K using data from [12]
889.8	[2]	Corrected from 293 K using data from [12]
**889.1 ± 0.9**		

Trimethylsilanol; $C_3H_{10}SiO$; Met3SiOH

For all the silanol molecules, no data at 298 K was found. There was also no systematic information about the temperature dependence of density. As such, we adopted the same value for the temperature correction of -1.3 kg·m^{-3}·K^{-1} as used previously for the alkoxysilanes. This is a somewhat crude approximation, given the different nature of the

functional groups involved; however, this is unlikely to have a very significant effect on the density values, since the correction is mostly applied over a range of 5 K, as discussed below.

We found four density values at 293 K: from Bažant et al. [4], Sauer [15], Sommer et al. [18] and Boksányi et al. [19]. All corrected values, together with their average and uncertainty, are shown in Table S19.

Table S19 *– Experimental data for density of Met3SiOH, together with the average value and uncertainty.*

Density at 298 K (kg/m^3)	Reference	Comments
805.7	[4]	Corrected from 293 K using data from [12]
805.7	[15]	Corrected from 293 K using data from [12]
808.6	[18]	Corrected from 293 K using data from [12]
809.5	[19]	Corrected from 293 K using data from [12]
**807.4 ± 2.0**		

Triethylsilanol; $C_6H_{16}SiO$; Eth3SiOH

Several density values were available at 293 K: from Yaws [2], Bažant et al. [4], Rochow [5], Eaborn [11], and Sommer et al. [18]. All corrected values, together with their average and uncertainty, are shown in Table S20.

Table S20 *– Experimental data for density of Eth3SiOH, together with the average value and uncertainty.*

Density at 298 K (kg/m^3)	Reference	Comments
858.2	[2]	Corrected from 293 K using data from [12]
857.3	[4]	Corrected from 293 K using data from [12]
858.0	[4]	Corrected from 293 K using data from [12]
858.2	[4]	Corrected from 293 K using data from [12]
859.5	[5]	Corrected from 293 K using data from [12]
857.5	[11]	Corrected from 293 K using data from [12]
857.3	[18]	Corrected from 293 K using data from [12]
**858.0 ± 0.6**		

Dimethylethylsilanol; $C_4H_{12}SiO$; M2ESiOH

Density values at 293 K were available from Yaws [2], Bažant et al. [4], and Boksányi et al. [19]. All corrected values, together with their average and uncertainty, are shown in Table S21.

Table S21 *– Experimental data for density of M2ESiOH, together with the average value and uncertainty.*

Density at 298 K (kg/m^3)	Reference	Comments
826.7	[2]	Corrected from 293 K using data from [12]
826.7	[4]	Corrected from 293 K using data from [12]
821.5	[19]	Corrected from 293 K using data from [12]
**825.0 ± 3.5**		

Methyldiethylsilanol; $C_5H_{14}SiO$; ME2SiOH

Only one density value was found at 293 K, reported by Bažant et al. [4]. This was corrected to 298 K as described above, yielding a density of **839.3 kg/m³** for ME2SiOH. No uncertainty could be estimated for this compound.

Hexamethyldisiloxane; $C_6H_{18}Si_2O$; Met6Si2O

Three density values at 298 K were available for this compound, from Yaws [12], Iseard [8] and Bažant et al. [4]. Yaws [12] also provided parameters for a correlation as a function of temperature, from which a correction term of -1.02 $kg \cdot m^{-3} \cdot K^{-1}$was estimated in the range 293-298 K. This is very close to values determined previously for methyl-substituted silanes (see Table S3), which suggests it is a reasonable estimate. This allowed us to correct several other values available at 293 K, from Bažant et al. [4], Rochow [5], Holland and Smyth [31], and Sauer [15]. The entire set of values, together with their average and estimated uncertainty, are shown in Table S22.

Table S22 *– Experimental data for density of Met6Si2O, together with the average value and uncertainty.*

Density at 298 K (kg/m³)	Reference	Comments
760.0	[12]	
758.0	[4]	
758.4	[8]	
758.2	[4]	Corrected from 293 K using data from [12]
756.8	[5]	Corrected from 293 K using data from [12]
758.8	[31]	Corrected from 293 K using data from [12]
758.7	[15]	Corrected from 293 K using data from [12]
**758.4 ± 0.8**		

S1.2 Enthalpy of vaporization

Literature data for the enthalpy of vaporization of organosilicon compounds was most often estimated from measurements of the vapor pressure over a range of temperatures, although some data (most notably that of Voronkov et al. [20]) was obtained from calorimetry measurements. We made extensive use of the compilations by Yaws [12], Chickos and Acree [21], and Stull [22] as well as other literature sources.

In many cases, whenever raw data for the vapor pressure was available, we carried out our own calculations following the procedure described by Chickos and Acree [21]. Specifically, the data were fitted to an equation of the form:

$$\log_{10} p = A - \frac{B}{C + T} \#(1)$$

with pressure (p) in mmHg and temperature (T) in Kelvin. Whenever possible, a narrow range of temperatures centered around 298 K was selected. This was not always possible, however, in which cases the calculated enthalpy needed to be corrected for the temperature difference (see below). Once the data for the vapor pressure was fit to equation (1), the enthalpy of vaporization (ΔH_{Vap}) was calculated from:

$$\Delta H_{\mathrm{Vap}}(T) = 2.303RB\left(\frac{T}{C+T}\right)^2 \#(2)$$

To correct for the effect of temperature, when the enthalpy of vaporization was determined at a temperature other than 298 K, we followed the procedure below:

1) In the small number of cases where the enthalpy was reported at several different temperatures for a given compound, the enthalpy at 298 K was estimated by linearly fitting the available data, as done above for density.

2) In some cases, coefficients for a correlation of ΔH_{Vap} as a function of T were reported by Yaws [12], from which a temperature correction was estimated for the corresponding compounds. Whenever possible, the correlation was used to estimate corrections for chemically similar compounds (i.e. the temperature dependence of ΔH_{Vap} was assumed to be the same for similar compounds).

3) When no temperature dependence data was available, the enthalpy was corrected using equation (3) [21]:

$$\Delta H_{\mathrm{Vap}}(T_2) = \Delta H_{\mathrm{vap}}(T_1) + \left(C_p^{\mathrm{Liq}} - C_p^{\mathrm{Gas}}\right)(T_1 - T_2)\#(3)$$

where C_p is the heat capacity in the liquid (superscript “Liq”) or gas (superscript “Gas”) phases. In most cases where the compound is liquid at 298 K, the experimental heat capacity of the gas at that temperature is not available. Therefore, we applied the following approximation [21]:

$$\Delta H_{\mathrm{Vap}}(298\ \mathrm{K}) = \Delta H_{\mathrm{Vap}}(T_1) + \left(10.58 + 0.26C_p^{\mathrm{Liq}}\right)(T_1 - 298)\#(4)$$

To apply equation (4), we collected experimental heat capacities in the liquid phase for all available compounds from NIST [23], and estimated those which were unavailable by interpolation or analogy with the corresponding carbon-based compounds. Figure S6 shows the heat capacity of Met4Si and Eth4Si, as well as methyl- and ethyl-substituted tetrahedral carbon compounds (e.g. the analogous compound of Met4Si is neopentane). It can be seen that the data for the carbon compounds follows a straight line with the number of ethyl substituents. The data for the two silicon compounds is offset upwards by a nearly constant amount, following a similar trend as their carbon analogues. This suggests that it is reasonable to estimate the heat capacities of the missing alkylsilanes through linear interpolation from the two available data points. The equation used for this interpolation is shown as an inset in Figure S6.

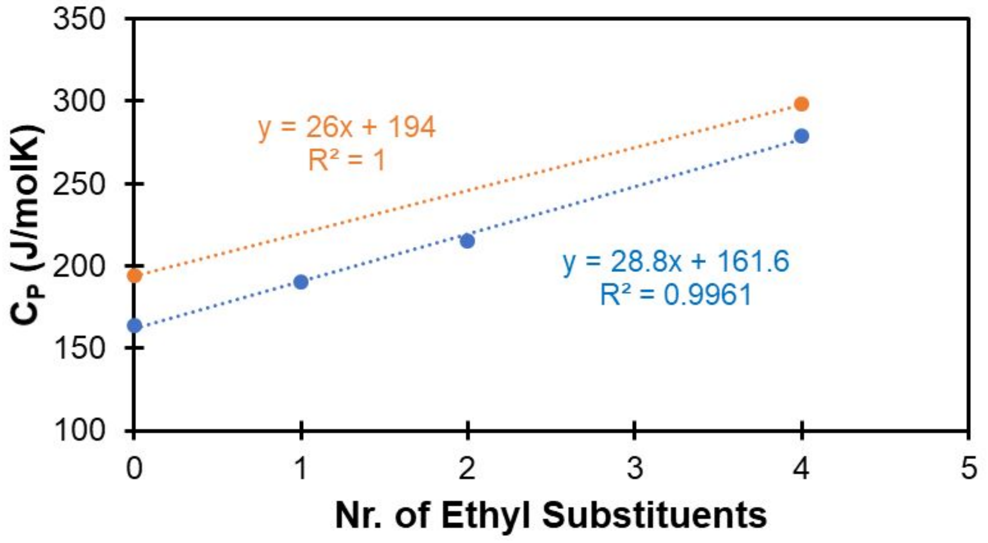

Figure S6 *– Heat capacity of tetrahedral carbon-based (blue) and silicon-based (orange) molecules with different number of ethyl substituents, the remainder being methyl substituents (e.g. 3 in the x-axis corresponds to M1E3Si or M1E3C). Also shown are linear fits through the data, with equations and correlation coefficients shown in the corresponding color.*

We were also able to obtain the heat capacity of tetramethoxysilane and tetraethoxysilane from NIST [23]. This allowed us to estimate the heat capacity of all alkoxysilanes by interpolating between Met4Si, Eth4Si, Si(OMet)4, and Si(OEth)4, depending on the number of methyl, ethyl, methoxy and ethoxy substituents.

Finally, we estimated the heat capacity of Met3SiOH and Eth3SiOH by analogy with their carbon-based analogues. Specifically, we assumed the same ratio between the heat capacity of the fully alkylated compound and the corresponding tertiary alcohol; in other words, we assumed that the effect of replacing an alkyl with a hydroxyl substituent was the same in carbon-based and silicon-based compounds. For example, the heat capacity of Met3SiOH was estimated by multiplying the value for Met4Si by the ratio of the heat capacities of 2-methyl-2-propanol and neopentane. Once this was done, we were able to estimate the heat capacities of M2ESiOH and ME2SiOH from those of Met3SiOH and Eth3SiOH by linear interpolation.

Table S23 shows the full set of heat capacity values used herein, with estimated values shown in italics.

Table S23 – *Heat capacities (J·mol^{-1}·K^{-1}) in the liquid phase for all organosilicon compounds considered here, used to correct for the temperature effect on the enthalpy of vaporization. Values estimated by linear interpolation are shown in italics. Other values were taken from NIST [23].*

**Compound name**	**Acronym**	**Formula**	**Heat Capacity (J/molK)**
Tetramethylsilane	Met4Si	$C_4H_{12}Si$	194
Trimethylethylsilane	M3E1Si	$C_5H_{14}Si$	*220*
Dimethyldiethylsilane	M2E2Si	$C_6H_{16}Si$	*246*
Methyltriethylsilane	M1E3Si	$C_7H_{18}Si$	*272*
Tetraethylsilane	Eth4Si	$C_8H_{20}Si$	298
Trimethylmethoxylsilane	M3SiOM	$C_4H_{12}SiO$	*206*
Trimethylethoxylsilane	M3SiOE	$C_5H_{14}SiO$	*233*
Triethylmethoxylsilane	E3SiOM	$C_7H_{18}SiO$	*287*
Triethylethoxylsilane	E3SiOE	$C_8H_{20}SiO$	*315*
Dimethyldimethoxylsilane	M2Si(OM)2	$C_4H_{12}SiO_2$	*217*
Methylethyldimethoxylsilane	MESi(OM)2	$C_5H_{14}SiO_2$	*246*
Diethyldimethoxylsilane	E2Si(OM)2	$C_6H_{16}SiO_2$	*274*
Dimethyldiethoxylsilane	M2Si(OE)2	$C_6H_{16}SiO_2$	*274*
Methylethyldiethoxylsilane	MESi(OE)2	$C_7H_{18}SiO_2$	*303*
Diethyldiethoxylsilane	E2Si(OE)2	$C_8H_{20}SiO_2$	*331*
Methyltrimethoxylsilane	MSi(OM)3	$C_4H_{12}SiO_3$	*229*
Ethyltrimethoxylsilane	ESi(OM)3	$C_5H_{14}SiO_3$	*259*
Methyltriethoxylsilane	MSi(OE)3	$C_7H_{18}SiO_3$	*318*
Ethyltriethoxylsilane	ESi(OE)3	$C_8H_{20}SiO_3$	*348*
Tetramethoxylsilane	Si(OMet)4	$C_4H_{12}SiO_4$	241
Tetraethoxylsilane	Si(OEth)4	$C_8H_{20}SiO_4$	364
Trimethylsilanol	Met3SiOH	$C_3H_{10}SiO$	*259*
Triethylsilanol	Eth3SiOH	$C_6H_{16}SiO$	*378*
Dimethylethylsilanol	M2ESiOH	$C_4H_{12}SiO$	*299*
Methyldiethylsilanol	ME2SiOH	$C_5H_{14}SiO$	*338*
Hexamethyldisiloxane	Met6Si2O	$C_6H_{18}Si_2O$	311

Tetramethylsilane; $C_4H_{12}Si$; Met4Si

Yaws reported parameters for the temperature dependence of ΔH_{Vap} for Met4Si, from which a correction of 0.061 kJ·mol^{-1}·K^{-1} was estimated. Encouragingly, the correction obtained by applying equation (4) with the experimental heat capacity value shown in Table S23 was exactly 0.061 kJ·mol^{-1}·K^{-1}, which supports the consistency of our approach. This correction was used to render all available vaporization enthalpy values at a temperature of 298 K.

Data for the vapor pressure as a function of temperature for Met4Si was available from Aston et al. [24] and Stull [22]. These data sets are shown in Figure S7 together with the corresponding linear fits.

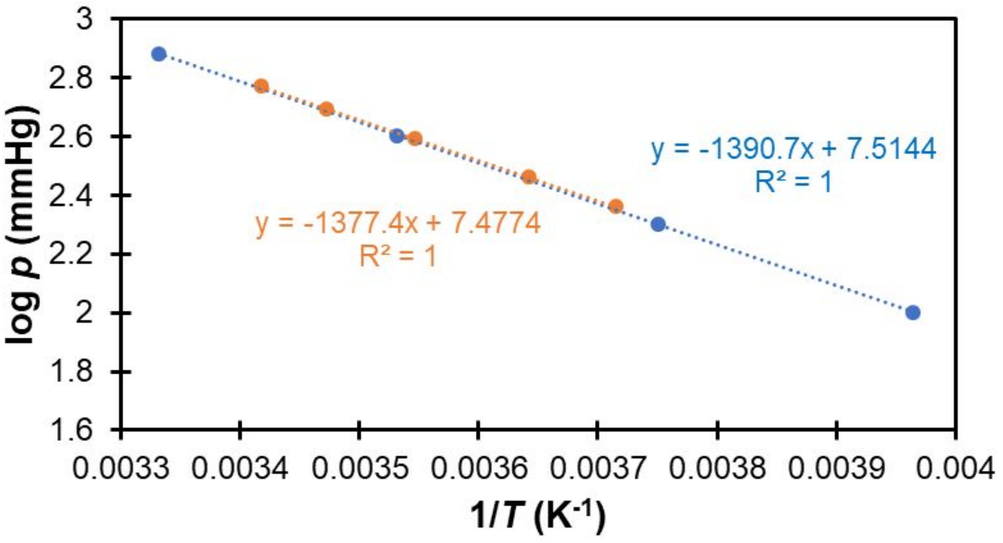

Figure S7 *– Logarithm of the vapor pressure as a function of inverse temperature for Met4Si. The data were obtained from Stull [22] (blue) and Aston et al. [24]. The equations and correlation coefficients for the corresponding linear fits are shown as insets with the same color code.*

From the slopes of these fits, the enthalpy of vaporization was calculated from equation (2) – notice that in this case, and in the majority of cases shown below, the constant C in equation (1) was taken as 0 as long as satisfactory fits were obtained. This led to values of 26.63 kJ/mol from the data of Stull [22] and 26.37 kJ/mol from Aston et al. [24]. However, it should be noticed that the temperature ranges, as well as the average temperatures (275.6 K for Stull and 281.3 K for Aston et al.) are different for the two data sets. The two values were thus corrected for the temperature effect as described above, yielding enthalpies at 298 K of 25.2 kJ/mol for Stull and 25.3 kJ/mol for Aston et al. It is quite reassuring that application of our analysis led to values that were almost identical when converted to the same temperature.

Data was also available at 298 K from Voronkov et al. [20] and Thomas et al. [25]. We note that the compilation of Chickos and Acree [21] reports the values by Voronkov et al. [20] and Aston et al. [24] under the entry for Met4Si, so they were not considered in the averaging process to avoid a bias due to duplicate values. This procedure was followed in all subsequent cases, so we only report data from Chickos and Acree [21] in our tables when the original source was not available. The full list of ΔH_{Vap} data for Met4Si is provided in Table S24, together with the average and uncertainty estimate.

Table S24 *– Experimental data for enthalpy of vaporization of Met4Si, together with the average value and uncertainty.*

ΔH_{Vap} at 298 K (kJ/mol)	Reference	Comments
25.2	[22]	From fit to vapor pressure data
25.3	[24]	From fit to vapor pressure data
26.0	[20]	
24.3	[25]	
25.3	[12]	From temperature correlation
**25.2 ± 0.5**		

Tetraethylsilane; $C_8H_{20}Si$; Eth4Si

Data from Stull [22] for the vapor pressure is plotted in Figure S8 together with the corresponding linear fit. The average temperature over the chosen range was 300.5 K, so only a small temperature correction was needed. This was estimated from the correlation of Yaws [12]. The full data set is shown in Table S25, together with the average and uncertainty estimate.

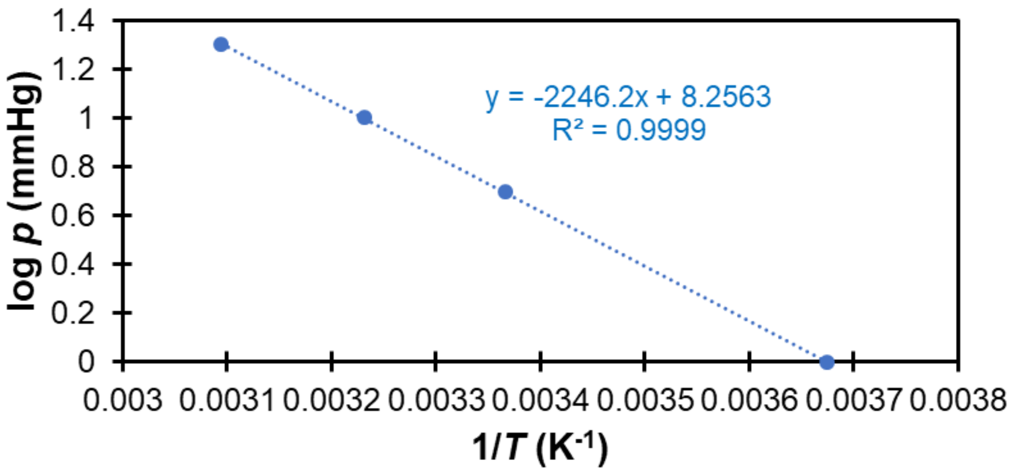

Figure S8 *– Logarithm of the vapor pressure as a function of inverse temperature for Eth4Si, together with the corresponding linear fit equation and correlation coefficient. The data were obtained from Stull [22].*

Table S25 *– Experimental data for enthalpy of vaporization of Eth4Si, together with the average value and uncertainty.*

ΔH_{Vap} at 298 K (kJ/mol)	Reference	Comments
43.1	[22]	From fit to vapor pressure data
39.0	[20]	
39.7	[10]	
45.3	[12]	From temperature correlation
**41.8 ± 3.0**		

Trimethylethylsilane; $C_5H_{14}Si$; M3E1Si

Data from Stull [22] for the vapor pressure is plotted in Figure S9 together with the corresponding linear fit. The average temperature over the chosen range was 300.8 K, so only a small temperature correction was needed. This was estimated from the correlation of Yaws for Met4Si [12]. The full data set is shown in Table S26, together with the average and uncertainty estimate.

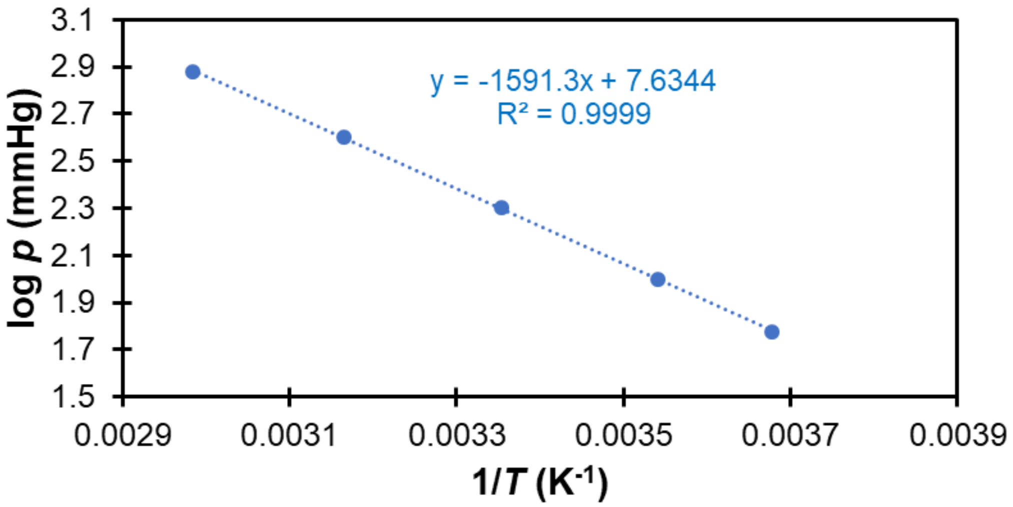

Figure S9 *– Logarithm of the vapor pressure as a function of inverse temperature for M3E1Si, together with the corresponding linear fit equation and correlation coefficient. The data were obtained from Stull [22].*

Table S26 *– Experimental data for enthalpy of vaporization of M3E1Si, together with the average value and uncertainty.*

ΔH_{Vap} at 298 K (kJ/mol)	Reference	Comments
30.6	[22]	From fit to vapor pressure data
30.1	[10]	
**30.4 ± 0.5**		

Dimethyldiethylsilane; $C_6H_{16}Si$; M2E2Si

Data for this compound was only available from Voronkov et al. [20] and from a chemical compounds website [13], from which an average and uncertainty were estimated (Table S27).

Table S27 *– Experimental data for enthalpy of vaporization of M2E2Si, together with the average value and uncertainty.*

ΔH_{Vap} at 298 K (kJ/mol)	Reference	Comments
38.9	[20]	
34.2	[13]	
**36.6 ± 4.7**		

Methyltriethylsilane; $C_7H_{18}Si$; M1E3Si

Data from Stull [22] for the vapor pressure is plotted in Figure S10 together with the corresponding linear fit. The average temperature over the chosen range was 302.9 K, so only a small temperature correction was needed. This was estimated from the correlation of Yaws for Eth4Si [12]. The full data set is shown in Table S28, together with the average and uncertainty estimate.

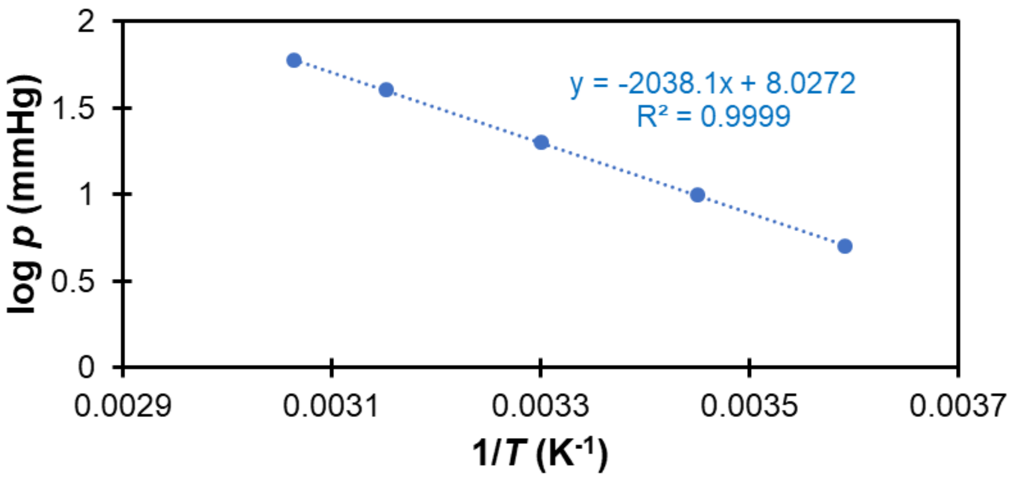

Figure S10 *– Logarithm of the vapor pressure as a function of inverse temperature for M1E3Si, together with the corresponding linear fit equation and correlation coefficient. The data were obtained from Stull [22].*

Table S28 *– Experimental data for enthalpy of vaporization of M1E3Si, together with the average value and uncertainty.*

**ΔH_{Vap} at 298 K (kJ/mol)**	**Reference**	**Comments**
39.3	[22]	From fit to vapor pressure data
40.5	[20]	
36.0	[10]	
**38.6 ± 2.1**		

Tetramethoxylsilane; $C_4H_{12}SiO_4$; Si(OMet)4

Data for the vapor pressure as a function of temperature for Si(OMet)4 obtained from Thomas et al. [25] and Kato and Tanaka [14] are plotted in Figure S11, together with the corresponding linear fit equations and correlation coefficients. The average temperatures for the two data sets over the chosen ranges were 329 K and 368 K, respectively, so the temperature corrections, estimated through equation (4), were quite significant. The full set of data, with average value and uncertainty, are shown in Table S29.

Table S29 *– Experimental data for enthalpy of vaporization of Si(OMet)4, together with the average value and uncertainty.*

**ΔH_{Vap} at 298 K (kJ/mol)**	**Reference**	**Comments**
43.3	[25]	From fit to vapor pressure data
43.5	[14]	From fit to vapor pressure data
41.4	[20]	
40.8	[12]	Corrected from T_b using equation (4)
**42.3 ± 1.4**		

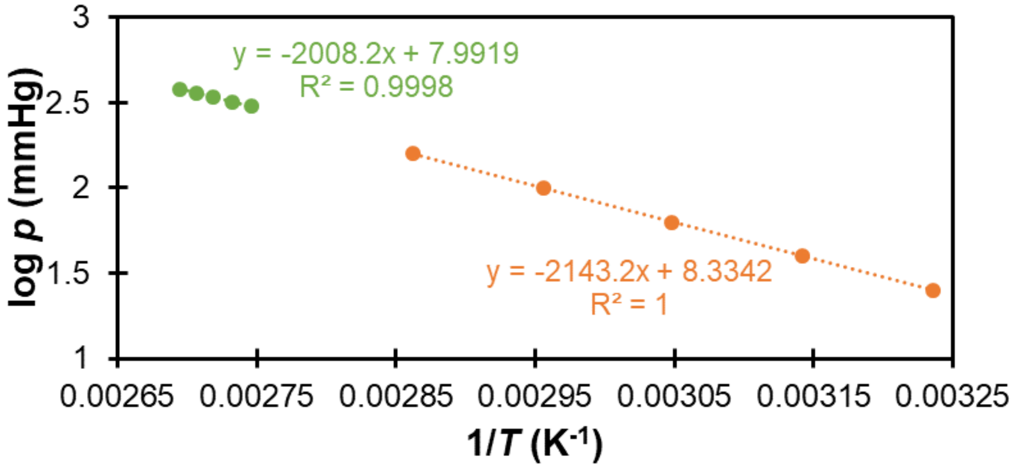

Figure S11 *– Logarithm of the vapor pressure as a function of inverse temperature for Si(OMet)4, together with the corresponding linear fit equations and correlation coefficients. The data were obtained from Thomas et al. [25] (orange) and Kato and Tanaka [14] (green).*

<u>Tetraethoxylsilane; $C_8H_{20}SiO_4$; Si(OEth)4</u>

Data for the vapor pressure as a function of temperature for Si(OMet)4 obtained from Stull [22], Thomas et al. [25] and Kato and Tanaka [14] are plotted in Figure S12, together with the corresponding linear fit equations and correlation coefficients. Bažant et al. [4] also report several values for liquid/vapor equilibrium, which are also plotted in Figure S12. The temperature corrections, estimated through equation (4), were mild for the first two data sets (average temperatures of 317 K and 329 K, respectively) but was quite significant for the latter two (average temperatures of 408 K and 373 K, respectively). Nevertheless, the corrected enthalpies were consistent with each other and with other reported values (see Table S30).

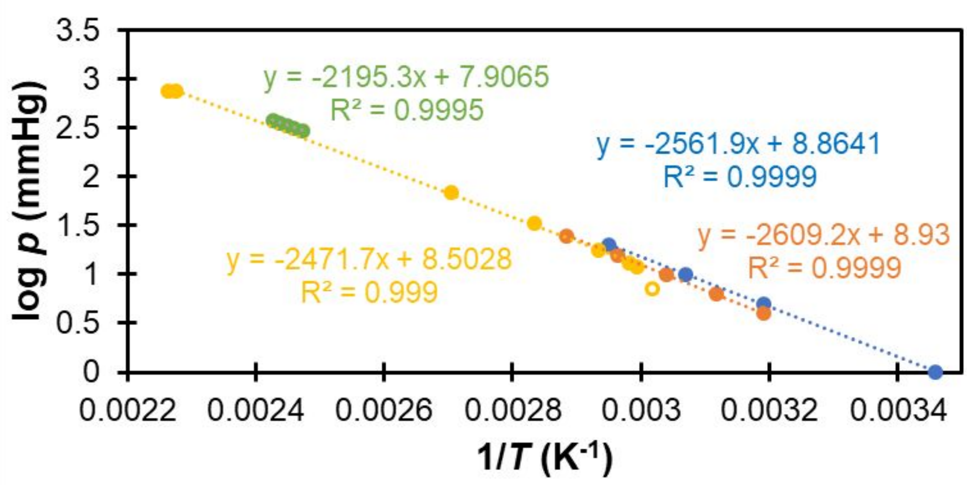

Figure S12 *– Logarithm of the vapor pressure as a function of inverse temperature for Si(OEth)4, together with the corresponding linear fit equation and correlation coefficient. The data were obtained from Stull [22] (blue), Thomas et al. [25] (orange), Kato and Tanaka [14] (green), and Bažant et al. [4] (yellow). One of the points reported by Bažant et al. (open circle) was not considered in the fit because it deviates significantly from the observed trend.*

The paper by van der Vis et al. [26] reported the enthalpy of vaporization as a function of temperature over a rather wide range. The data were well described by a linear fit (see Figure S13), from which an extrapolated value at 298 K was calculated.

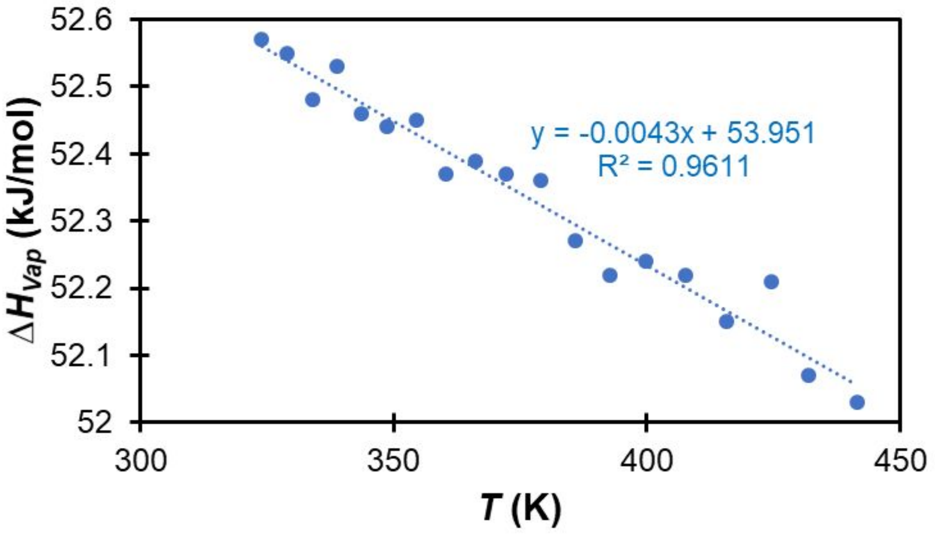

Figure S13 *– Enthalpy of vaporization of Si(OEth)4 as a function of temperature, together with the corresponding linear fit equation and correlation coefficient. The data were obtained from van der Vis et al. [26].*

Table S30 *– Experimental data for enthalpy of vaporization of Si(OEth)4, together with the average value and uncertainty.*

ΔH_{Vap} at 298 K (kJ/mol)	Reference	Comments
51.0	[22]	From fit to vapor pressure data
53.2	[25]	From fit to vapor pressure data
53.6	[14]	From fit to vapor pressure data
55.2	[4]	From fit to vapor pressure data
48.5	[20]	
52.7	[26]	Extrapolated to 298 K
53.5	[12]	Corrected from T_b using equation (4)
**52.5 ± 1.6**		

Trimethylmethoxylsilane; $C_4H_{12}SiO$; M3SiOM

Only one data point was available: Yaws [12] reported the enthalpy of vaporization at the boiling point, which for M3SiOM is 330.6 K. The value was corrected for 298 K by applying equation (4) with the estimated heat capacity from Table S23, yielding a value of **30.0 kJ/mol**. Notice that if one were to estimate the correction from the coefficients of the Yaws correlation for M2Si(OM)2 [12], a very similar compound, one would have obtained an enthalpy of 29.8 kJ/mol, very close to the value above. This gives an idea of the error arising from the approximations employed in the temperature correction. However, because only one value was available, we were not able to estimate an experimental uncertainty for the enthalpy of vaporization of this compound.

Triethylmethoxylsilane; $C_7H_{18}SiO$; E3SiOM

No experimental data for ΔH_{Vap} was found for this compound.

Trimethylethoxylsilane; $C_5H_{14}SiO$; M3SiOE

Data from Stull [22] for the vapor pressure is plotted in Figure S14 together with the corresponding linear fit. The average temperature over the chosen range was 299.5 K, so only a very small temperature correction was needed. This was estimated from equation (4) with the heat capacity from Table S23.

Yaws [12] also reported the value of ΔH_{Vap} at the boiling point (349.15 K), while Voronkov et al. [20] also reported an enthalpy of vaporization for this compound. The full data set is shown in Table S31, together with the average and uncertainty estimate.

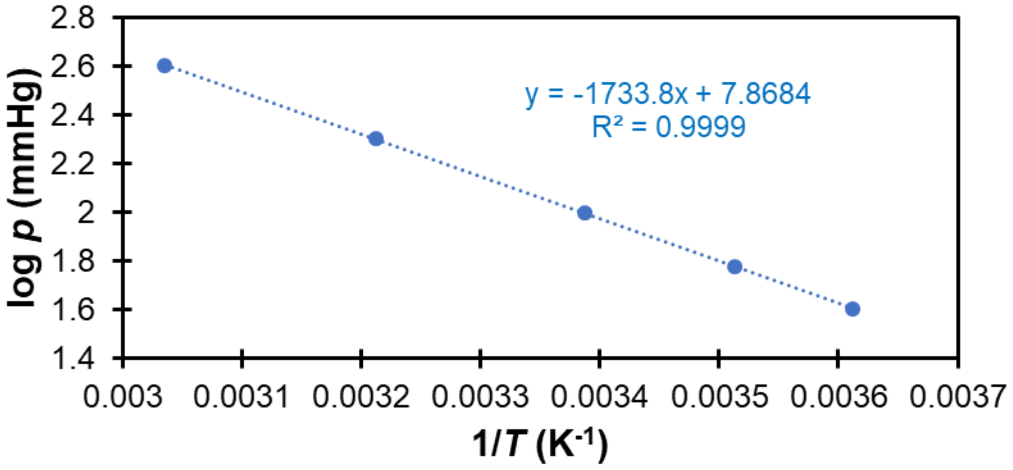

Figure S14 *– Logarithm of the vapor pressure as a function of inverse temperature for M3SiOE, together with the corresponding linear fit equation and correlation coefficient. The data were obtained from Stull [22].*

Table S31 *– Experimental data for enthalpy of vaporization of M3SiOE, together with the average value and uncertainty.*

ΔH_{Vap} at 298 K (kJ/mol)	Reference	Comments
33.3	[22]	From fit to vapor pressure data
38.4	[20]	
33.2	[12]	Corrected from T_b using equation (4)
**35.0 ± 3.4**		

Triethylethoxylsilane; $C_8H_{20}SiO$; E3SiOE

A single data point was found, reported by Yaws [12] at the boiling point, which was corrected for 298 K using equation (4). The value obtained was **48.9 kJ/mol**. No uncertainty could be estimated, but since the boiling temperature (427.65 K) is quite far from 298 K, the uncertainty in this value is expected to be rather high.

Dimethyldimethoxylsilane; $C_4H_{12}SiO_2$; M2Si(OM)2

The only source of data we found for the enthalpy of vaporization of M2Si(OM)2 was a correlation reported by Yaws [12]. This allowed us to calculate a value of **33.7 kJ/mol** for this compound at 298 K. No uncertainty could be estimated.

Methylethyldimethoxylsilane; $C_5H_{14}SiO_2$; MESi(OM)2

No data was found for this compound.

Diethyldimethoxylsilane; $C_6H_{16}SiO_2$; E2Si(OM)2

No data was found for this compound.

Dimethyldiethoxylsilane; $C_6H_{16}SiO_2$; M2Si(OE)2

Data from Stull [22] for the vapor pressure is plotted in Figure S15 together with the corresponding linear fit. The average temperature over the chosen range was 298 K, so no temperature correction was needed.

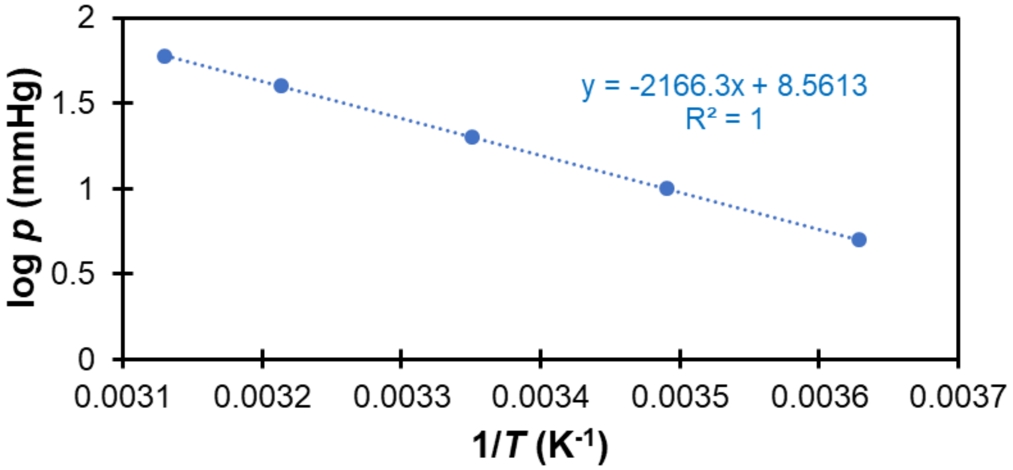

Figure S15 *– Logarithm of the vapor pressure as a function of inverse temperature for M2Si(OE)2, together with the corresponding linear fit equation and correlation coefficient. The data were obtained from Stull [22].*

Data was also available from Voronkov et al. [20] at 298 K and from Yaws [12] at the boiling point (387.15 K), which was corrected to 298 K using equation (4) and the heat capacity value from Table S23. The full set of data, together with average and uncertainty, is shown in Table S32.

Table S32 *– Experimental data for enthalpy of vaporization of M2Si(OE)2, together with the average value and uncertainty.*

ΔH_{Vap} at 298 K (kJ/mol)	Reference	Comments
41.5	[22]	From fit to vapor pressure data
43.1	[20]	
40.4	[12]	Corrected from T_b using equation (4)
**41.7 ± 1.6**		

Methylethyldiethoxylsilane; $C_7H_{18}SiO_2$; MESi(OE)2

No data was found for this compound.

Diethyldiethoxylsilane; $C_8H_{20}SiO_2$; E2Si(OE)2

No data was found for this compound.

Methyltrimethoxylsilane; $C_4H_{12}SiO_3$; MSi(OM)3

Data was available for MSi(OM)3 from Voronkov et al. [20] at 298 K and from Yaws [12] at the boiling point (375.65 K), which was corrected to 298 K using equation (4) and the heat capacity value from Table S23. The full set of data, together with average and uncertainty, is shown in Table S33.

Table S33 *– Experimental data for enthalpy of vaporization of MSi(OM)3, together with the average value and uncertainty.*

ΔH_{Vap} at 298 K (kJ/mol)	Reference	Comments
34.3	[20]	
37.5	[12]	Corrected from T_b using equation (4)
**35.9 ± 3.2**		

Methyltriethoxylsilane; $C_7H_{18}SiO_3$; MSi(OE)3

Data from Stull [22] for the vapor pressure is plotted in Figure S16 together with the corresponding linear fit. The average temperature over the chosen range was 298.8 K, so a nearly negligible temperature correction was needed, obtained from equation (4). We also fitted the vapor/liquid equilibrium data collected by Bažant et al. [4], also shown in Figure S16, which was corrected to 298 K from an average temperature of 351 K.

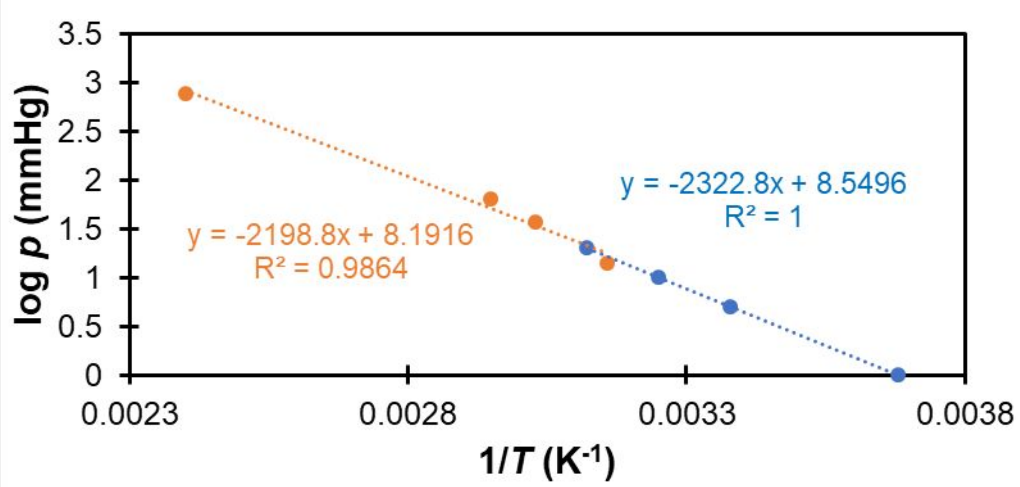

Figure S16 *– Logarithm of the vapor pressure as a function of inverse temperature for MSi(OE)3, together with the corresponding linear fit equations and correlation coefficients. The data were obtained from Stull [22] and Bažant et al. [4].*

Data was also available from Voronkov et al. [20] at 298 K and from Yaws [12] at the boiling point (415.15 K), which was corrected to 298 K using equation (4) and the heat capacity

value from Table S23. The full set of data, together with average and uncertainty, is shown in Table S34.

***Table S34** – Experimental data for enthalpy of vaporization of MSi(OE)3, together with the average value and uncertainty.*

**ΔH_{Vap} at 298 K (kJ/mol)**	**Reference**	**Comments**
44.5	[22]	From fit to vapor pressure data
45.3	[4]	From fit to vapor pressure data
45.1	[20]	
46.7	[12]	Corrected from T_b using equation (4)
**45.4 ± 0.9**		

Ethyltrimethoxylsilane; $C_5H_{14}SiO_3$; ESi(OM)3

A single data point was found, reported by Yaws [12] at the boiling point, which was corrected for 298 K using equation (4). The value obtained was **41.9 kJ/mol**. No uncertainty could be estimated, but since the boiling temperature (397.45 K) is quite far from 298 K, the uncertainty in this value is expected to be rather high.

Ethyltriethoxylsilane; $C_8H_{20}SiO_3$; ESi(OE)3

Data for the vapor pressure as a function of temperature was available from Jenkins and Chambers [27]. This data is plotted in Figure S17, together with the corresponding linear fit equation and correlation coefficient. From this fit, an enthalpy of vaporization for ESi(OE)3 at the average temperature of 375.4 K was calculated, and subsequently corrected to 298 K using equation (4). We also fitted the vapor/liquid equilibrium data collected by Bažant et al. [4], also shown in Figure S17, which was corrected to 298 K from an average temperature of 377 K. Data was also available from Yaws [12] at the boiling point (431.65 K), which was corrected to 298 K using equation (4) and the heat capacity value from Table S23. The full set of data, together with average and uncertainty, is shown in Table S35.

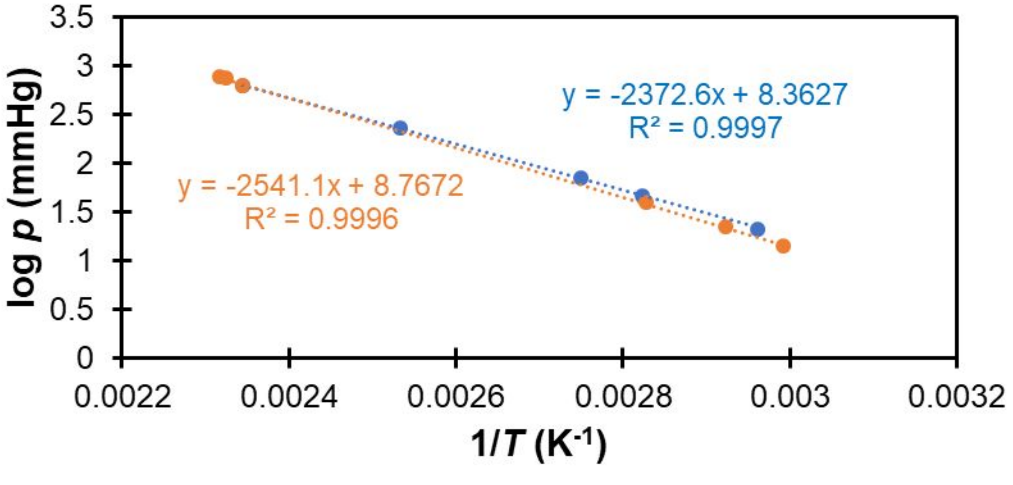

***Figure S17** – Logarithm of the vapor pressure as a function of inverse temperature for ESi(OE)3, together with the corresponding linear fit equations and correlation coefficients. The data were obtained from Jenkins and Chambers [27] and Bažant et al. [4].*

Table S35 *– Experimental data for enthalpy of vaporization of MSi(OE)3, together with the average value and uncertainty.*

**ΔH_{Vap} at 298 K (kJ/mol)**	**Reference**	**Comments**
53.2	[27]	From fit to vapor pressure data
56.7	[4]	From fit to vapor pressure data
50.8	[12]	Corrected from T_b using equation (4)
**53.6 ± 3.4**		

Trimethylsilanol; $C_3H_{10}SiO$; Met3SiOH

Grubb and Osthoff [28] reported data for the vapor pressure of Met3SiOH as a function of temperature. This data is plotted in Figure S28, together with the linear fit equation and correlation coefficient. An enthalpy of vaporization was obtained at an average temperature of 298 K, for which no temperature correction was needed.

Two additional data points were also reported by Chickos and Acree [21], one of which required a correction from 306 K, and another by Bažant et al. [4]. Temperature corrections were estimated via equation (4) with the heat capacities reported in Table S23. The full data set, together with average and uncertainty, is reported in Table S36.

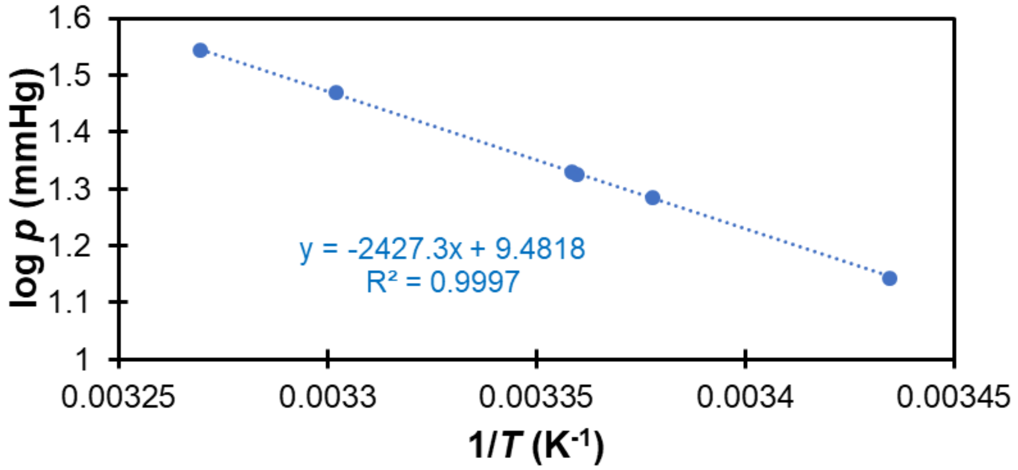

Figure S18 *– Logarithm of the vapor pressure as a function of inverse temperature for Met3SiOH, together with the corresponding linear fit equation and correlation coefficient. The data were obtained from Grubb and Osthoff [28].*

Table S36 *– Experimental data for enthalpy of vaporization of Met3SiOH, together with the average value and uncertainty.*

**ΔH_{Vap} at 298 K (kJ/mol)**	**Reference**	**Comments**
46.5	[28]	From fit to vapor pressure data
47.4	[21]	Corrected from 306 K using equation (4)
45.7	[21]	
41.5	[4]	
**45.3 ± 2.6**		

Triethylsilanol; $C_6H_{16}SiO$; Eth3SiOH

Data for the vapor pressure of Eth3SiOH as a function of temperature reported by Grubb and Osthoff [28] is plotted in Figure S28, together with the linear fit equation and correlation coefficient. An enthalpy of vaporization was obtained at an average temperature of 319 K, which was then corrected to 298 K using equation (4). A fit was also carried out over several vapor/liquid equilibrium data reported by Bažant et al. [4], together with a temperature correction from an average temperature of 347.5 K in that data set. Yaws [12] also reported the enthalpy of vaporization of Eth3SiOH at the boiling point (427 K), which was corrected to 298 K using the same method. The full data set, together with average and uncertainty, is reported in Table S37. We notice the significant discrepancy between all three enthalpy values, which leads to a very large uncertainty on this property.

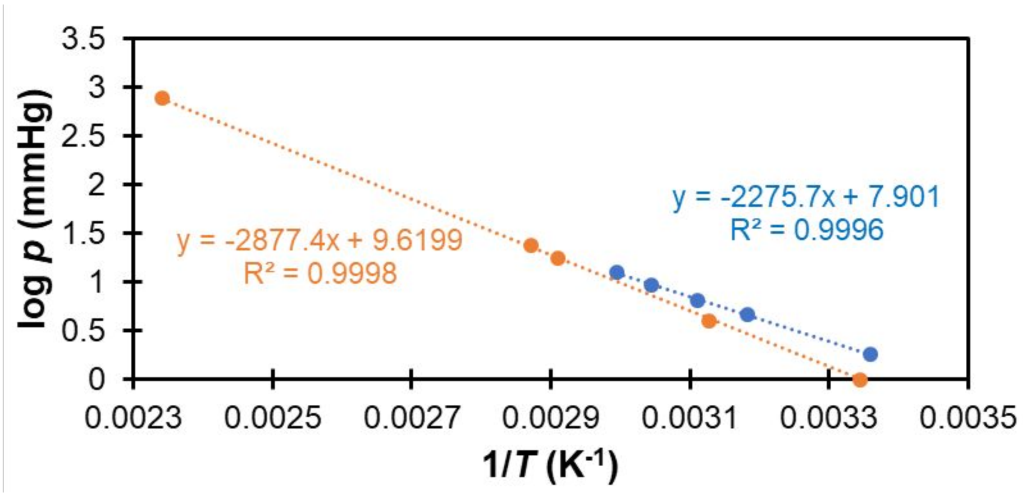

Figure S19 *– Logarithm of the vapor pressure as a function of inverse temperature for Eth3SiOH, together with the corresponding linear fit equations and correlation coefficients. The data were obtained from Grubb and Osthoff [28], and Bažant et al. [4].*

Table S37 *– Experimental data for enthalpy of vaporization of Eth3SiOH, together with the average value and uncertainty.*

ΔH_{Vap} at 298 K (kJ/mol)	Reference	Comments
45.9	[28]	From fit to vapor pressure data
60.5	[4]	From fit to vapor pressure data
51.0	[12]	Corrected from T_b using equation (4)
**52.5 ± 8.6**		

Dimethylethylsilanol; $C_4H_{12}SiO$; M2ESiOH

A single data point was found, reported by Yaws [12] at the boiling point, which was corrected for 298 K using equation (4). The value obtained was **41.7 kJ/mol**. No uncertainty could be estimated, but since the boiling temperature (393 K) is quite far from 298 K, the uncertainty in this value is expected to be rather high.

Methyldiethylsilanol; $C_5H_{14}SiO$; ME2SiOH

No data was found for this compound.

Hexamethyldisiloxane; $C_6H_{18}Si_2O$; Met6Si2O

Vapor pressure data for Met6Si2O was obtained from three sources: Stull [22], Scott et al. [29] and Flaningam [30]. The data are plotted in Figure S20 together with the corresponding linear fit equations and correlation coefficients. The enthalpies of vaporization were obtained from the slopes of the fits as described above, and were corrected for a temperature of 298 K. The temperature corrections were estimated from the correlation reported by Yaws [12], from which we also calculated an enthalpy of vaporization at 298 K. Two other enthalpy values were reported by Chickos and Acree [21], one of which required a temperature correction from 327 K. An additional enthalpy value was reported by Iseard et al. [8]. The full data set, together with average and uncertainty, is reported in Table S38.

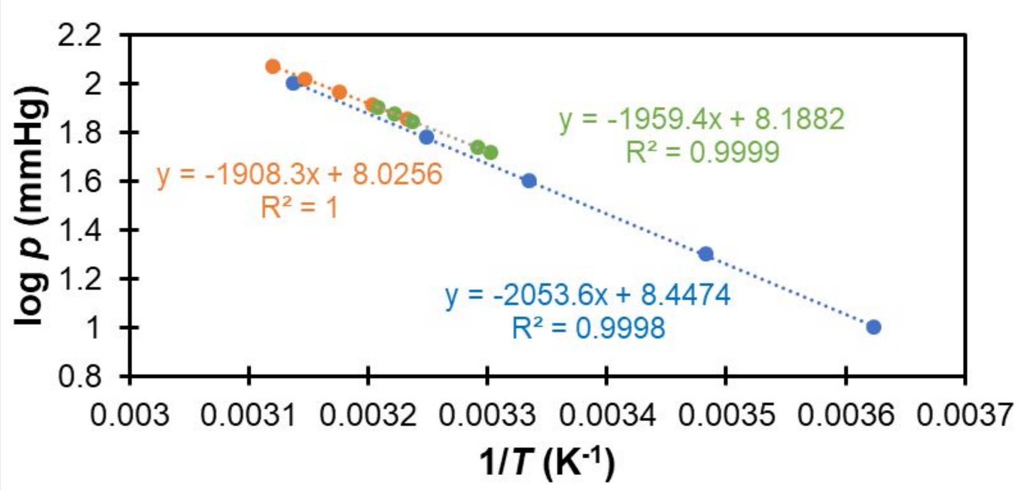

Figure S20 *– Logarithm of the vapor pressure as a function of inverse temperature for Met6Si2O, obtained from Stull [22] (blue), Scott et al. [29] (orange), and Flaningam [30] (green). The corresponding linear fit equations and correlation coefficients are shown as insets with the same color code.*

Table S38 *– Experimental data for enthalpy of vaporization of Met6Si2O, together with the average value and uncertainty.*

ΔH_{Vap} at 298 K (kJ/mol)	Reference	Comments
39.3	[22]	From fit to vapor pressure data
37.6	[29]	From fit to vapor pressure data
38.1	[30]	From fit to vapor pressure data
35	[21]	Corrected from 327 K using data from [12]
37.2	[21]	
37.2	[8]	
37.5	[12]	From temperature correlation
**37.4 ± 1.0**		

S1.3 Self-solvation free energies

Self-solvation free energies (i.e., corresponding to the free energy of transferring a single solute molecule to a solution at infinite dilution, for the special case when the solute and solvent are the same compound) can be related by thermodynamic expressions to the vaporization free energy and to the vapor pressure [58]. In this work, self-solvation free energies were calculated from the experimental vapor pressure at 298 K using equation (5):

$$\Delta G_{\mathrm{Solv}} = -RT.\ln\left(\frac{24.774\rho}{M_W p_{\mathrm{Vapor}}}\right) \#(5)$$

where R is the ideal gas constant, T is the temperature, ρ is the density in kg/m^3, M_W is the molecular weight in g/mol, and p_{Vapor} is the vapor pressure in bar. Experimental densities are available for all compounds, as described in section S1.1. Here, we describe the process of gathering and curating experimental vapor pressures for application of equation (5).

As discussed in section S1.2, data for the vapor pressure as a function of temperature was available for many compounds. In those cases, the vapor pressure at 298 K was obtained by fitting the data to equation (1) and interpolating or extrapolating to the desired temperature. In other cases, Antoine coefficients were available, from which the vapor pressure at 298 K was calculated.

Finally, for compounds where none of the above were available, we estimated the vapor pressure from the boiling point and the enthalpy of vaporization using the Clausius-Clapeyron equation:

$$\frac{\mathrm{d}p}{\mathrm{d}T} = \frac{\Delta H_{\mathrm{Vap}} p}{RT^2} \#(6)$$

This was integrated between two points on the vapor/liquid equilibrium curve, taking into account the temperature dependence of the enthalpy of vaporization using equation (4). This yielded equation (7):

$$p_{\mathrm{Vapor}} = p_0 \exp\left[-\frac{\Delta H_{\mathrm{Vap}}}{R}\left(\frac{1}{298{,}15} - \frac{1}{T_0}\right) - \frac{10.58 + 0.26 * C_P^l}{R}\left(1 - \frac{298{,}15}{T_0} + \ln\frac{298{,}15}{T_0}\right)\right] \#(7)$$

where p_0 and T_0 are a reference pressure and temperature, respectively, for vapor-liquid equilibrium of the selected compound (normally the boiling temperature at a pressure of 1 atm).

Whenever possible, several estimates of the vapor pressure were averaged so that an uncertainty could be estimated. The entries below report all the data for vapor pressures compiled from literature sources. The uncertainty (ε) was then propagated to the solvation free energy using equation (8):

$$\varepsilon_{\Delta G} = RT\sqrt{\left(\frac{\varepsilon_\rho}{\rho}\right)^2 + \left(\frac{\varepsilon_{P_V}}{P_V}\right)^2} \#(8)$$

Tetramethylsilane; $C_4H_{12}Si$; Met4Si

Vapor pressure data from Aston et al. [24] and Stull [22] is shown in Figure S7, from which values at 298 K were interpolated. Antoine coefficients are available from Yaws [12], while Flaningam [30] also reports coefficients for a correlation of vapor pressure vs temperature. This provides four independent estimates, from which an average and uncertainty can be calculated (Table S39). It is worth noting that several values estimated from the boiling point at 1 atm using equation (6) were found to be consistent with those reported in Table S39. However, because this method is expected to be less precise, they were not considered in the averaging procedure.

Table S39 *– Experimental data for vapor pressure of Met4Si, together with the average value and uncertainty.*

p_{Vapor} at 298 K (bar)	Reference	Comments
0.944	[22]	From fit to p_{Vapor} data
0.961	[24]	From fit to p_{Vapor} data
0.960	[12]	From Antoine equation
0.958	[30]	From correlation
**0.956 ± 0.008**		

Tetraethylsilane; $C_8H_{20}Si$; Eth4Si

The vapor pressure at 298 K was interpolated from data of Stull [22], shown in Figure S8, as well as from Antoine coefficients reported by Yaws [12]. The data are shown in Table S40.

Table S40 *– Experimental data for vapor pressure of Eth4Si, together with the average value and uncertainty.*

p_{Vapor} at 298 K (bar)	Reference	Comments
0.00704	[22]	From fit to p_{Vapor} data
0.00750	[12]	From Antoine equation
**0.00727 ± 0.00046**		

Trimethylethylsilane; $C_5H_{14}Si$; M3E1Si

The vapor pressure at 298 K was interpolated from data from Stull [22], shown in Figure S9. Two other identical values were found at 298 K, reported by Whitmore et al. [10] and by Bažant et al. [4]. The three values were averaged and an uncertainty was estimated, as shown in Table S41.

Table S41 *– Experimental data for vapor pressure of M3E1Si, together with the average value and uncertainty.*

p_{Vapor} at 298 K (bar)	Reference	Comments

0.264	[22]	From fit to p_{Vapor} data
0.267	[10]	
0.267	[4]	
**0.266 ± 0.002**		

Dimethyldiethylsilane; $C_6H_{16}Si$; M2E2Si

Vapor pressure data at 298 K for this compound was not available, with the exception of a value reported in a chemical compounds website [13]. We therefore also estimated the vapor pressure from the boiling points reported by Eaborn [11] and Bažant et al. [4], using equation (6). The three values, together with average and uncertainty, are shown in Table S42.

Table S41 *– Experimental data for vapor pressure of M2E2Si, together with the average value and uncertainty.*

p_{Vapor} at 298 K (bar)	Reference	Comments
0.0844	[13]	
0.07051	[11]	From boiling point via equation (6)
0.06961	[4]	From boiling point via equation (6)
**0.07484 ± 0.01**		

Methyltriethylsilane; $C_7H_{18}Si$; M1E3Si

The vapor pressure at 298 K was interpolated from data from Stull [22], shown in Figure S10. We also extrapolated the vapor pressure to 298 K from two points reported by Whitmore et al. [10]. The two values thus allowed us to estimate an uncertainty for this compound, as shown in Table S42.

Table S42 *– Experimental data for vapor pressure of M1E3Si, together with the average value and uncertainty.*

p_{Vapor} at 298 K (bar)	Reference	Comments
0.02072	[22]	From fit to p_{Vapor} data
0.02284	[10]	Extrapolated from two data points.
**0.02178 ± 0.002**		

Tetramethoxylsilane; $C_4H_{12}SiO_4$; Si(OMet)4

The vapor pressure at 298 K was interpolated from data from Thomas et al. [25], shown in Figure S11, and extrapolated from the Antoine coefficients reported by Kato et al. [14]. A correlation was also available from Yaws [12], from which we calculated the vapor pressure at 298 K. Finally, Bažant et al. [4] reported a value for the vapor pressure of Si(OMet)4 at 298 K. The full data set, together with average and uncertainty, is reported in Table S43.

Table S43 *– Experimental data for vapor pressure of Si(OMet)4, together with the average value and uncertainty.*

p_{Vapor} at 298 K (bar)	Reference	Comments
0.01866	[25]	From fit to p_{Vapor} data
0.02168	[14]	From Antoine coefficients

0.0183	[12]	From temperature correlation
0.016	[4]	
**0.01866 ± 0.002**		

Tetraethoxylsilane; $C_8H_{20}SiO_4$; Si(OEth)4

The vapor pressure at 298 K was interpolated from data from Thomas et al. [25], Stull [22], and Bažant et al. [4], shown in Figure S12. Correlations were also available from Yaws [12] and from van der Vis et al. [26], from which we calculated the vapor pressure at 298 K. Antoine coefficients were reported by Kato et al. [14], but the range of applicability was too distant from 298 K – extrapolation to 298 K using those coefficients led to a vapor pressure of 0.004338 bar, which was much higher than any of the other values (see Table S44 for the full data set).

Table S44 *– Experimental data for vapor pressure of Si(OEth)4, together with the average value and uncertainty.*

p_{Vapor} at 298 K (bar)	Reference	Comments
0.001992	[25]	From fit to p_{Vapor} data
0.002466	[22]	From fit to p_{Vapor} data
0.002155	[4]	From fit to p_{Vapor} data
0.001734	[26]	From temperature correlation
0.00117	[12]	From temperature correlation
**0.001903 ± 0.0004**		

Trimethylmethoxylsilane; $C_4H_{12}SiO$; M3SiOM

No data for the vapor pressure at 298 K or as a function of pressure was found for this compound. As such, the vapor pressure was estimated from several reported values of the boiling point at around 1 atm, using equation (6). These values, together with the average and uncertainty estimate, are shown in Table S45. We note that the actual uncertainty in this value is likely to be much higher due to the approximations involved in the procedure.

Table S45 *– Experimental data for vapor pressure of M3SiOM, together with the average value and uncertainty.*

p_{Vapor} at 298 K (bar)	Reference	Comments
0.3258	[15]	From boiling point via equation (6)
0.3315	[4]	From boiling point via equation (6)
0.3260	[5]	From boiling point via equation (6)
0.3325	[11]	From boiling point via equation (6)
0.3214	[12]	From boiling point via equation (6)
**0.3274 ± 0.004**		

Triethylmethoxylsilane; $C_7H_{18}SiO$; E3SiOM

No vapor pressure data was available for this compound. Also, because no enthalpy of vaporization is available, we were not able to apply equation (6) to boiling point data.

Trimethylethoxylsilane; $C_5H_{14}SiO$; M3SiOE

The vapor pressure at 298 K was interpolated from data from Stull [22], shown in Figure S14, yielding a value of **0.1507 bar**. No uncertainty could be estimated for this compound.

Triethylethoxylsilane; $C_8H_{20}SiO$; E3SiOE

No data for the vapor pressure at 298 K or as a function of pressure was found for this compound. As such, the vapor pressure was estimated from several reported values of the boiling point at around 1 atm, using equation (6). These values, together with the average and uncertainty estimate, are shown in Table S46. We note that the actual uncertainty in this value is likely to be much higher due to the approximations involved in the adopted procedure.

Table S46 *– Experimental data for vapor pressure of E3SiOE, together with the average value and uncertainty.*

p_{Vapor} at 298 K (bar)	Reference	Comments
0.009551	[4]	From boiling point via equation (6)
0.009854	[11]	From boiling point via equation (6)
0.009551	[12]	From boiling point via equation (6)
**0.009652 ± 0.0003**		

Dimethyldimethoxylsilane; $C_4H_{12}SiO_2$; M2Si(OM)2

No data for the vapor pressure at 298 K or as a function of pressure was found for this compound. As such, the vapor pressure was estimated from several reported values of the boiling point at around 1 atm, using equation (6). These values, together with the average and uncertainty estimate, are shown in Table S47. We note that the actual uncertainty in this value is likely to be much higher due to the approximations involved in the adopted procedure.

Table S47 *– Experimental data for vapor pressure of M2Si(OM)2, together with the average value and uncertainty.*

p_{Vapor} at 298 K (bar)	Reference	Comments
0.1277	[4]	From boiling point via equation (6)
0.1301	[11]	From boiling point via equation (6)
0.1306	[12]	From boiling point via equation (6)
**0.1303 ± 0.002**		

Methylethyldimethoxylsilane; $C_5H_{14}SiO_2$; MESi(OM)2

No vapor pressure data was available for this compound. Also, because no enthalpy of vaporization is available, we were not able to apply equation (6) to boiling point data.

Diethyldimethoxylsilane; $C_6H_{16}SiO_2$; E2Si(OM)2

No vapor pressure data was available for this compound. Also, because no enthalpy of vaporization is available, we were not able to apply equation (6) to boiling point data.

Dimethyldiethoxylsilane; $C_6H_{16}SiO_2$; M2Si(OE)2

The vapor pressure at 298 K was interpolated from data from Stull [22], shown in Figure S15. A correlation was also available from Yaws [12], from which we calculated the vapor pressure at 298 K. Both values are quite consistent, leading to a small uncertainty (Table S48).

Table S48 *– Experimental data for vapor pressure of M2Si(OE)2, together with the average value and uncertainty.*

p_{Vapor} at 298 K (bar)	Reference	Comments
0.02633	[22]	From fit to p_{Vapor} data
0.0260	[12]	From temperature correlation
**0.02617 ± 0.0003**		

Methylethyldiethoxylsilane; $C_7H_{18}SiO_2$; MESi(OE)2

No vapor pressure data was available for this compound. Also, because no enthalpy of vaporization is available, we were not able to apply equation (6) to boiling point data.

Diethyldiethoxylsilane; $C_8H_{20}SiO_2$; E2Si(OE)2

No vapor pressure data was available for this compound. Also, because no enthalpy of vaporization is available, we were not able to apply equation (6) to boiling point data.

Methyltrimethoxylsilane; $C_4H_{12}SiO_3$; MSi(OM)3

No data for the vapor pressure at 298 K or as a function of pressure was found for this compound. As such, the vapor pressure was estimated from several reported values of the boiling point at 1 atm, using equation (6). These values, together with the average and uncertainty estimate, are shown in Table S49. We note that the actual uncertainty in this value is likely to be much higher due to the approximations involved in the adopted procedure.

Table S49 *– Experimental data for vapor pressure of MSi(OM)3, together with the average value and uncertainty.*

p_{Vapor} at 298 K (bar)	Reference	Comments
0.06372	[4]	From boiling point via equation (6)
0.06129	[4]	From boiling point via equation (6)
0.06290	[11]	From boiling point via equation (6)
0.06290	[12]	From boiling point via equation (6)
**0.0627 ± 0.001**		

Methyltriethoxylsilane; $C_7H_{18}SiO_3$; MSi(OE)3

The vapor pressure at 298 K was interpolated from data from Stull [22], and from Bažant et al. [4], shown in Figure S16.

Table S50 *– Experimental data for vapor pressure of MSi(OE)3, together with the average value and uncertainty.*

p_{Vapor} at 298 K (bar)	Reference	Comments
0.007653	[22]	From fit to p_{Vapor} data
0.008744	[4]	From fit to p_{Vapor} data
**0.0082 ± 0.001**		

Ethyltrimethoxylsilane; $C_5H_{14}SiO_3$; ESi(OM)3

No data for the vapor pressure at 298 K or as a function of pressure was found for this compound. As such, the vapor pressure was estimated from several reported values of the boiling point at 1 atm, using equation (6). These values, together with the average and uncertainty estimate, are shown in Table S51. We note that the actual uncertainty in this value is likely to be much higher due to the approximations involved in the adopted procedure.

Table S51 *– Experimental data for vapor pressure of ESi(OM)3, together with the average value and uncertainty.*

p_{Vapor} at 298 K (bar)	Reference	Comments
0.02021	[4]	From boiling point via equation (6)
0.02112	[11]	From boiling point via equation (6)
0.02112	[12]	From boiling point via equation (6)
**0.02082 ± 0.0006**		

Ethyltriethoxylsilane; $C_8H_{20}SiO_3$; ESi(OE)3

The vapor pressure at 298 K was interpolated from data from Jenkins et al. [22], and from Bažant et al. [4], shown in Figure S17.

Table S52 *– Experimental data for vapor pressure of ESi(OE)3, together with the average value and uncertainty.*

p_{Vapor} at 298 K (bar)	Reference	Comments
0.003388	[27]	From fit to p_{Vapor} data
0.002340	[4]	From fit to p_{Vapor} data
**0.002864 ± 0.001**		

Trimethylsilanol; $C_3H_{10}SiO$; Met3SiOH

The vapor pressure at 298 K was interpolated from data from Grubb and Osthoff [28], shown in Figure S18, yielding a value of **0.02921 bar**. No other data was found, so no uncertainty could be estimated for this compound.

Triethylsilanol; $C_6H_{16}SiO$; Eth3SiOH

The vapor pressure at 298 K was interpolated from data from Grubb and Osthoff [28], shown in Figure S19. Bažant et al. [4] also reported the vapor pressure at 298 K. Both values, together with the average and uncertainty, are shown in Table S53. We notice the significant discrepancy between the two values, leading to a rather large uncertainty.

Table S53 *– Experimental data for vapor pressure of Eth3SiOH, together with the average value and uncertainty.*

p_{Vapor} at 298 K (bar)	Reference	Comments
0.002473	[28]	From fit to p_{Vapor} data
0.001255	[4]	
**0.001864 ± 0.001**		

Dimethylethylsilanol; $C_4H_{12}SiO$; M2ESiOH

No data for the vapor pressure at 298 K or as a function of pressure was found for this compound. As such, the vapor pressure was estimated from two reported values of the boiling point, using equation (6). These values, together with the average and uncertainty estimate, are shown in Table S54. We notice that the two values are quite different, hence the estimated uncertainty is high.

Table S54 *– Experimental data for vapor pressure of M2ESiOH, together with the average value and uncertainty.*

p_{Vapor} at 298 K (bar)	Reference	Comments
0.01320	[4]	From boiling point via equation (6)
0.02521	[12]	From boiling point via equation (6)
**0.01920 ± 0.012**		

Methyldiethylsilanol; $C_5H_{14}SiO$; ME2SiOH

No vapor pressure data was available for this compound. Also, because no enthalpy of vaporization is available, we were not able to apply equation (6) to boiling point data.

Hexamethyldisiloxane; $C_6H_{18}Si_2O$; Met6Si2O

The vapor pressure at 298 K was interpolated from data from Stull [22], Scott et al. [29] and Flaningam [30], shown in Figure S20. A correlation from Yaws [12] was also available, from which the vapor pressure was estimated at 298 K. The full data set, together with average and uncertainty, is reported in Table S55.

Table S55 *– Experimental data for vapor pressure of Met6Si2O, together with the average value and uncertainty.*

p_{Vapor} at 298 K (bar)	Reference	Comments
0.04836	[22]	From fit to p_{Vapor} data
0.05624	[29]	From fit to p_{Vapor} data
0.05511	[30]	From fit to p_{Vapor} data

0.0540	[12]	From temperature correlation
**0.05343 ± 0.0035**		

S1.4 Electronic properties and polarization corrections

Electronic properties are essential to estimate the polarization corrections as described in the main paper. This requires each molecule's dipole moment (μ), polarizability (α), static dielectric constant (ε) and refractive index (n_D) (from which the infinite-frequency dielectric constant can be estimated). The experimental dielectric constant, when available, was also used to validate the molecular models. The dipole moments, dielectric constants and refractive indices at the sodium D-line frequency were obtained from several literature sources, as indicated below, from which average values were calculated. Polarizabilities were obtained from the ChemSpider website [32]. Molecules for which certain properties were not available are shown as blank entries in the tables below.

Tetramethylsilane; $C_4H_{12}Si$; Met4Si

Table S56 *– Experimental electronic properties of Met4Si, together with the average values.*

μ (D)	α (Å^3)	ε	n_D	Reference
0		1.921	1.3588	[33]
			1.359	[4]
			1.3582	[12]
			1.3588	[11]
	11.6			[32]
**0**	**11.6**	**1.921**	**1.3587**	

Tetraethylsilane; $C_8H_{20}Si$; Eth4Si

Table S57 *– Experimental electronic properties of Eth4Si, together with the average values.*

μ (D)	α (Å^3)	ε	n_D	Reference
0		2.09	1.4269	[33]
			1.4252	[4]
			1.4268	[12]
			1.4268	[10]
	19.0			[32]
**0**	**19.0**	**2.09**	**1.4264**	

Trimethylethylsilane; $C_5H_{14}Si$; M3E1Si

Data for the dielectric constant or dipole moment of M3E1Si was not found. However, the dipole moment is expected to be very close to zero, in line with the data for Met4Si and Eth4Si. As such, the polarization corrections are expected to also be zero.

Dimethyldiethylsilane; $C_6H_{16}Si$; M2E2Si

Data for the dielectric constant or dipole moment of M2E2Si was not found. However, the dipole moment is expected to be very close to zero, in line with the data for Met4Si and Eth4Si. As such, the polarization corrections are expected to also be zero.

Methyltriethylsilane; $C_7H_{18}Si$; M1E3Si

Data for the dielectric constant or dipole moment of M1E3Si was not found. However, the dipole moment is expected to be very close to zero, in line with the data for Met4Si and Eth4Si. As such, the polarization corrections are expected to also be zero.

Tetramethoxylsilane; $C_4H_{12}SiO_4$; Si(OMet)4

Table S58 *– Experimental electronic properties of Si(OMet)4, together with the average values.*

μ (D)	α (Å^3)	ε	n_D	Reference
			1.37	[4]
			1.3683	[12]
			1.3672	[14]
1.78				[34]
1.75				[35]
1.78				[36]
		6.0		[37]
	14.4			[32]
**1.77**	**14.4**	**6.0**	**1.3685**	

Tetraethoxylsilane; $C_8H_{20}SiO_4$; Si(OEth)4

Table S59 *– Experimental electronic properties of Si(OEth)4, together with the average values.*

μ (D)	α (Å^3)	ε	n_D	Reference
			1.383	[4]
			1.3928	[12]
			1.3815	[14]
1.63				[34]
1.75				[35]
1.70				[38]
		4.1		[37]
	21.7			[32]
**1.693**	**21.7**	**4.1**	**1.3858**	

Trimethylmethoxylsilane; $C_4H_{12}SiO$; M3SiOM

Table S60 *– Experimental electronic properties of M3SiOM, together with the average values.*

μ (D)	α (Å^3)	ε	n_D	Reference
			1.3678	[4]
			1.367	[12]
			1.3675	[11]

			1.3678	[15]
1.18				[38]
1.19				[39]
1.21		3.25		[40]
	12.3			[32]
**1.193**	**12.3**	**3.25**	**1.3675**	

Triethylmethoxylsilane; $C_7H_{18}SiO$; E3SiOM

The index of refraction was reported by Bažant et al. [4] as **1.4129**, while the dipole moment has been reported as **1.13 D** [40] and the polarizability as **17.8 Å³** [32]. However, no dielectric constant was found for this compound. In fact, no dielectric constants or dipole moments could be found for the other ethlyalkoxysilanes (i.e. derivatives with ethyl substituents instead of methyl). For this reason, it was assumed that the polarization corrections for the ethlyalkoxysilanes were identical to the corresponding methlyalkoxysilanes – i.e. the value for E3SiOM was assumed to be the same as that for M3SiOM.

Trimethylethoxylsilane; $C_5H_{14}SiO$; M3SiOE

Table S61 *– Experimental electronic properties of M3SiOE, together with the average values.*

μ (D)	α (Å³)	ε	n_D	Reference
			1.3712	[4]
			1.3741	[12]
			1.3742	[11]
			1.3743	[15]
1.17				[35]
1.17				[41]
1.18		3.01		[40]
		2.3		[42]
	14.1			[32]
**1.173**	**14.1**	**2.66**	**1.3735**	

Triethylethoxylsilane; $C_8H_{20}SiO$; E3SiOE

No data was found. Polarization corrections were assumed to be the same as for M3SiOE.

Dimethyldimethoxylsilane; $C_4H_{12}SiO_2$; M2Si(OM)2

Table S62 *– Experimental electronic properties of M2Si(OM)2, together with the average values.*

μ (D)	α (Å³)	ε	n_D	Reference
			1.3708	[4]
1.31			1.3708	[12]
			1.3706	[11]
1.29				[35]
1.33				[36]
1.37		3.66		[40]
	13.0			[32]

**1.325**	**13.0**	**3.66**	**1.3707**	

Methylethyldimethoxylsilane; $C_5H_{14}SiO_2$; MESi(OM)2

No data was found. Polarization corrections were assumed to be the same as for M2Si(OM)2.

Diethyldimethoxylsilane; $C_6H_{16}SiO_2$; E2Si(OM)2

No data was found. Polarization corrections were assumed to be the same as for M2Si(OM)2.

Dimethyldiethoxylsilane; $C_6H_{16}SiO_2$; M2Si(OE)2

Table S63 *– Experimental electronic properties of M2Si(OE)2, together with the average values.*

μ (D)	α (Å^3)	ε	n_D	Reference
			1.3708	[4]
			1.3839	[16]
			1.3811	[12]
			1.3814	[11]
1.39				[35]
1.36				[41]
1.36		3.22		[40]
		2.36		[42]
	16.7			[32]
**1.37**	**16.7**	**2.79**	**1.3814**	

Methylethyldiethoxylsilane; $C_7H_{18}SiO_2$; MESi(OE)2

No data was found. Polarization corrections were assumed to be the same as for M2Si(OE)2.

Diethyldiethoxylsilane; $C_8H_{20}SiO_2$; E2Si(OE)2

No data was found. Polarization corrections were assumed to be the same as for M2Si(OE)2.

Methyltrimethoxylsilane; $C_4H_{12}SiO_3$; MSi(OM)3

Table S64 *– Experimental electronic properties of MSi(OM)3, together with the average values.*

μ (D)	α (Å^3)	ε	n_D	Reference
			1.3687	[4]
			1.3696	[12]
			1.369	[11]
1.68				[35]
1.6				[36]
1.78		4.9		[40]
	13.7			[32]
**1.687**	**13.7**	**4.9**	**1.3691**	

Ethyltrimethoxylsilane; $C_5H_{14}SiO_3$; ESi(OM)3

No data was found. Polarization corrections were assumed to be the same as for MSi(OM)3.

Ethyltriethoxylsilane; $C_8H_{20}SiO_3$; ESi(OE)3

No data was found. Polarization corrections were assumed to be the same as for MSi(OE)3.

Methyltriethoxylsilane; $C_7H_{18}SiO_3$; MSi(OE)3

Table S65 *– Experimental electronic properties of MSi(OE)3, together with the average values.*

μ (D)	α (Å^3)	ε	n_D	Reference
			1.3832	[4]
			1.3832	[12]
			1.3838	[16]
			1.3807	[17]
1.72				[35]
1.7				[41]
1.71		3.85		[40]
		2.63		[42]
	19.2			[32]
**1.71**	**19.2**	**3.24**	**1.3827**	

Trimethylsilanol; $C_3H_{10}SiO$; Met3SiOH

Table S66 *– Experimental electronic properties of Met3SiOH, together with the average values.*

μ (D)	α (Å^3)	ε	n_D	Reference
			1.388	[4]
			1.388	[15]
			1.3888	[18]
			1.3896	[19]
2.01		7.17		[28]
1.53				[40]
	10.4			[32]
**1.77**	**10.4**	**7.17**	**1.3886**	

Triethylsilanol; $C_6H_{16}SiO$; Eth3SiOH

Table S67 *– Experimental electronic properties of Eth3SiOH, together with the average values.*

μ (D)	α (Å^3)	ε	n_D	Reference
			1.4329	[4]
			1.4341	[4]
			1.4329	[4]
			1.4329	[12]
			1.4329	[18]
			1.4341	[11]
0.62		2.66		[28]
0.62				[43]

1.52				[40]
	15.9			[32]
**0.917**	**15.9**	**2.66**	**1.4333**	

Dimethylethylsilanol; $C_4H_{12}SiO$; M2ESiOH

No data was found. Polarization corrections were interpolated from the values for Met3SiOH and Eth3SiOH.

Methyldiethylsilanol; $C_5H_{14}SiO$; ME2SiOH

No data was found. Polarization corrections were interpolated from the values for Met3SiOH and Eth3SiOH.

Hexamethyldisiloxane; $C_6H_{18}Si_2O$; Met6Si2O

Table S68 *– Experimental electronic properties of Met6Si2O, together with the average values.*

μ (D)	α (Å^3)	ε	n_D	Reference
			1.3774	[4]
			1.3741	[4]
0.66			1.3777	[12]
0.46		2.179		[31]
0.78				[35]
0.66		2.17		[40]
	19.4			[32]
**0.64**	**19.4**	**2.175**	**1.3764**	

Table S69 compiles all the electronic properties of the studied compounds, and reports the calculated values of the liquid phase dipole moment and the polarization corrections, as described in the main paper.

Table S69 *– Experimental electronic properties of organosilicates, and estimated polarization corrections for vapor/liquid phase change properties. All the calculations are for pure liquids (i.e. enthalpy of vaporization and self-solvation free energy) and assume a change from vapor to liquid – i.e. the distortion energy is positive. Blank entries indicate that experimental data was not found; in those cases, the polarization corrections were estimated by analogy or interpolation (see footnotes).*

Molecule	μ_{Gas} (D)	α (Å^3)	ε	n_D	μ_{Liq} (D)[1]	E_{Elec} (kJ/mol)	E_{Dist} (kJ/mol)	E_{Pol} (kJ/mol)[2]
Met4Si	0	11.6	1.92	1.3587	0.00	0.00	0.00	0.00
Eth4Si	0	19.0	2.09	1.4264	0.00	0.00	0.00	0.00
M3E1Si	0[3]	13.5		1.3820	0.00	0.00	0.00	0.00
M2E2Si	0[3]	15.3		1.4017	0.00	0.00	0.00	0.00
M1E3Si	0[3]	17.1		1.4160	0.00	0.00	0.00	0.00
Si(OMet)4	1.765	14.4	6.00	1.3686	2.64	1.60	-2.31	-0.71
Si(OEth)4	1.693	21.7	4.10	1.3858	2.43	0.75	-1.39	-0.65
M3SiOM	1.193	12.3	3.25	1.3675	1.61	0.42	-1.00	-0.58
E3SiOM	1.13	17.8		1.4129				-0.58[4]
M3SiOE	1.173	14.1	2.66	1.3735	1.52	0.26	-0.80	-0.54
E3SiOE		19.7		1.4398				-0.54[4]
M2Si(OM)2	1.325	13.0	3.66	1.3707	1.83	0.60	-1.24	-0.65
MESi(OM)2				1.3854				-0.65[4]
E2Si(OM)2		16.7		1.3988				-0.65[4]
M2Si(OE)2	1.37	16.7	2.69	1.3814	1.79	0.32	-0.97	-0.65
MESi(OE)2				1.3950				-0.65[4]
E2Si(OE)2		20.3		1.4022				-0.65[4]
MSi(OM)3	1.687	13.7	4.90	1.3691	2.45	1.28	-2.09	-0.82
ESi(OM)3		15.5		1.3838				-0.82[4]
MSi(OE)3	1.71	19.2	3.24	1.3827	2.33	0.61	-1.44	-0.83
ESi(OE)3		21.0		1.3930				-0.83[4]
Met3SiOH	1.77	10.4	7.17	1.3889	2.78	2.95	-3.86	-0.91
Eth3SiOH	0.917	15.9	2.66	1.4333	1.24	0.20	-0.60	-0.40
M2ESiOH		12.2		1.4048				-0.74[5]
ME2SiOH				1.4206				-0.57[5]
Met6Si2O	0.64	19.4	2.175	1.3764	0.79	0.04	-0.16	-0.12

[1]Estimated from a Kirkwood-Onsager polarizable continuum model; [2]For a liquid to vapor change (e.g. enthalpy of vaporization), the correction has the opposite sign; [3]Assumed zero by analogy with the other tetraalkylsilanes; [4]Assumed equal to the value for the corresponding methyl-substituted alkoxysilane; [5]Interpolated linearly between Met3SiOH and Eth3SiOH.

S1.5 Experimental data for model parameterization and validation

Table S70 compiles the experimental data used for model development and validation.

Table S70 *– Compilation of experimental properties of organosilicates used for model development and validation. With the exception of the molecular weight (M_W) and dielectric constant, each property value is followed by its uncertainty (±) in the same units. Individual literature values and references are listed in the above sections.*

Molecule	M_W (g/mol)	ρ (kg/m³)	±	ΔH_{vap} (kJ/mol)	±	p_{Vapor} (bar)	±	ΔG_{solv} (kJ/mol)	±	ε
Met4Si	88.2243	638.3	2.8	25.2	0.5	0.9558	0.0080	-12.97	0.023	1.92
M3E1Si	102.251	679.5	0.7	30.4	0.5	0.2659	0.0016	-15.93	0.015	
M2E2Si	116.2777	712.1	0.1	36.6	4.7	0.07484	0.0096	-18.87	0.32	
M1E3Si	130.3044	739.4		38.6	2.1	0.02178	0.0021	-21.73	0.24	
Eth4Si	144.3311	762.1	0.4	41.8	3.0	0.007270	0.00046	-24.27	0.16	2.09
Met3SiOH	90.197	807.4	2.0	45.3	2.6	0.02921		-22.14	0.006	7.17
M2ESiOH	104.224	825.0	3.5	41.7		0.01920	0.012	-22.87	1.55	
ME2SiOH	118.251	839.3								
Eth3SiOH	132.278	858.0	0.6	52.5	8.6	0.001864	0.0012	-28.16	1.62	2.66
M3SiOM	104.224	753.0	2.7	30.0		0.3274	0.0041	-15.62	0.032	3.25
E3SiOM	146.3	813.8								
M3SiOE	118.251	751.2	0.4	35.0	3.4	0.1507		-17.22	0.001	2.66
E3SiOE	160.33	821.7	14.1	43.5	10.9	0.009652	0.00030	-23.50	0.089	
M2Si(OM)2	120.22	857.8	0.6	33.7		0.1303	0.0018	-17.87	0.034	3.66
MESi(OM)2	134.25	866.6								
E2Si(OM)2	148.28	875.3								
M2Si(OE)2	148.28	853.8	24.8	41.7	1.6	0.02617	0.00033	-21.32	0.079	2.69
MESi(OE)2	162.3	837.5								
E2Si(OE)2	176.33	849.1	13.2							
MSi(OM)3	136.22	949.7	1.6	35.9	3.2	0.06270	0.0010	-19.62	0.040	4.90
MSi(OE)3	178.3	889.0	6.6	45.4	0.9	0.008199	0.0011	-23.83	0.33	
ESi(OM)3	150.25	942.4	0.1	41.9		0.02082	0.00061	-22.09	0.072	3.24
ESi(OE)3	192.3	889.1	0.9	53.6	3.4	0.002864	0.0010	-26.25	0.91	
Si(OMet)4	152.22	1024.2	4.6	42.3	1.4	0.01866	0.0023	-22.54	0.31	6.00
Si(OEth)4	208.33	926.6	2.0	52.5	1.6	0.001903	0.00044	-27.17	0.57	4.10
Met6Si2O	162.3	758.4	0.8	37.4	1.0	0.05343	0.0035	-19.03	0.16	2.175

S2. Point Charge Determination

Point charges for organosilicate molecules were obtained from DFT calculations on selected prototypical molecules using the DDEC method [44]. In order for the point charges to effectively describe the liquid-phase environment of each molecule, each molecule was optimized within an IEFPCM polarizable continuum model [45]. Due to the lack of organosilicate solvent parameters available in Gaussian09, alternative solvents were selected among those available based on the similarity of the static dielectric constant and/or molecular structure. Table S71 lists the solvents selected for each organosilicate molecule, together with their dielectric constants.

Table S71 *– List of organosilicate molecules used to obtain point charge values for the force field, together with the organic solvent used in the IEFPCM calculations and its corresponding static dielectric constant.*

**Organosilicate molecule**	**IEFPCM solvent**	ε
Met4Si	n-Nonane	1.9605
Eth4Si	n-Nonane	1.9605
Met3SiOH	2,6-Dimethyl pyridine	7.1735
Eth3SiOH	Pentanoic Acic	2.6924
Met6Si2O	Dibutyl Ether	3.0473
SiOMet4	Diethyl Ether	4.2400
SiOEth4	Diethyl Ether	4.2400
M3SiOE	Diisopropyl Ether	3.3800
M2ESiOE	Diisopropyl Ether	3.3800
ME2SiOE	Dibutyl Ether	3.0473
E3SiOE	Dibutyl Ether	3.0473

S3. Bond and Angle parameters

Having surveyed the literature, we were able to find several sources with quantum calculations for the bond-stretching and angle-bending parameters of silicon-containing molecules. The bulk of the parameters were found in two quite comprehensive papers by Grigoras and Lane [46] and Abraham and Grant [47], which contained QM calculations for approximately 25 and 15 organosilica molecules, respectively, covering all of the different chemical families studied in this work. However, those studies are both from 1988 and use a relatively low level of theory (i.e. 3-21G* basis set). Therefore, we endeavoured, whenever possible, to compare them to experimental values and more recent calculations to assess their validity and, if needed, to refine the parameters.

S3.1 Bond lengths

For the Si–C bond length, we decided to use the experimental value for tetramethylsilane ($Si(CH_3)_4$), reported as 0.1875 nm [48]. The value reported by Grigoras and Lane [46] is 0.1896 nm, which is slightly longer, but this is for methylsilane (SiH_3CH_3), which only has one alkyl substituent and therefore does not belong to the class of molecules that we are focusing on.

Next, the values for Si–O_H are compared. The value reported by Grigoras and Lane [46] is 0.1653 nm, which compares very well with a more recent study [49] at a much higher level of theory, i.e. CCSD(T), with the cc-pVQZ basis sets, which reports 0.16525 nm. The same study reports a slightly smaller value of 0.16481 nm with an even larger basis set (cc-pV5Z). However, these results are for silanol (SiH_3OH), and it is likely that the addition of further alkyl substituents will lead to a slight increase in the Si–O_H bond length [46]. All things considered, we opted to keep the original value of 0.1653 nm in our model.

For the length of the siloxane bond, i.e. Si–O_B Grigoras and Lane report a value of 0.1645 nm [46], while Abraham and Grant cite 0.164 nm [47], both for the disiloxane molecule (SiH_3OSiH_3). There are two experimental values for disiloxane in the literature: 0.1634 nm [50] and 0.1631 nm [51], but again this molecule has no substituents on the Si atoms, in contrast with the molecules we wish to model that contain tetra-substituted Si atoms. Assuming that each additional methyl substituent increases the Si–O_B bond by 0.0003 nm (the increase observed for silanols, as discussed above), we could extrapolate the experimental bond lengths to a fully substituted siloxane molecule (i.e. hexamethyldisiloxane), yielding a range between 0.1631 nm and 0.1634 nm. Since the original value of Abraham and Grant [47] falls within this range, we have used this value in the model.

Finally, we move onto the Si–O_C bond. The value from Grigoras and Lane [46] is 0.1656 nm for dimethylmethoxysilane (i.e. $(CH_3)_2SiHOCH_3$). They also provide a value of 0.1652 nm for methylmethoxysilane (i.e. $CH_3SiH_2OCH_3$), which again shows how adding methyl substituents slightly lengthens Si–O bonds. Since the former molecule is already almost fully substituted, we have opted to use the original value of Grigoras and Lane [46] in our model.

S3.2 Angles

Equilibrium angles were taken from the work of Grigoras and Lane [46]. Their value for the C–Si–C angle (112°) reported for dimethylsilane is in reasonable agreement with the electron diffraction value of 110.5° for hexamethyldisilane [48], while their value of 149.5° for the Si–O_B–Si angle in disiloxane is also close to the experimental value of 151.2° and more recent theoretical work pointing to a value of 150.2° [52].

The form of the angle bending potential in the study of Grigoras and Lane [46] includes an anharmonic term of the form:

$$U_{\text{Angle}} = \frac{k_{\text{Angle}}}{2}(\theta - \theta_0)^2\left[1 + k_6(\theta - \theta_0)^4\right]\#$$

where θ_0 is the equilibrium bond angle, θ is the actual angle between the three bonded atoms, k_A is the angle force constant, and k_6 is the force constant for the sixth-order anharmonic term. We present a comparison of the harmonic part and the full anharmonic potential in Figure S21 for the C–Si–C angle. It can be seen that the harmonic potential describes the full anharmonic term quite well near the energy minimum, up to a deviation of the equilibrium angle of about 10 degrees, where the energetic barrier is approximately 10 kJ/mol. Since this is a good deal larger than the scale of thermal fluctuations at room temperature (~2.5 kJ/mol), using only the harmonic term is a reasonable approximation for our purposes.

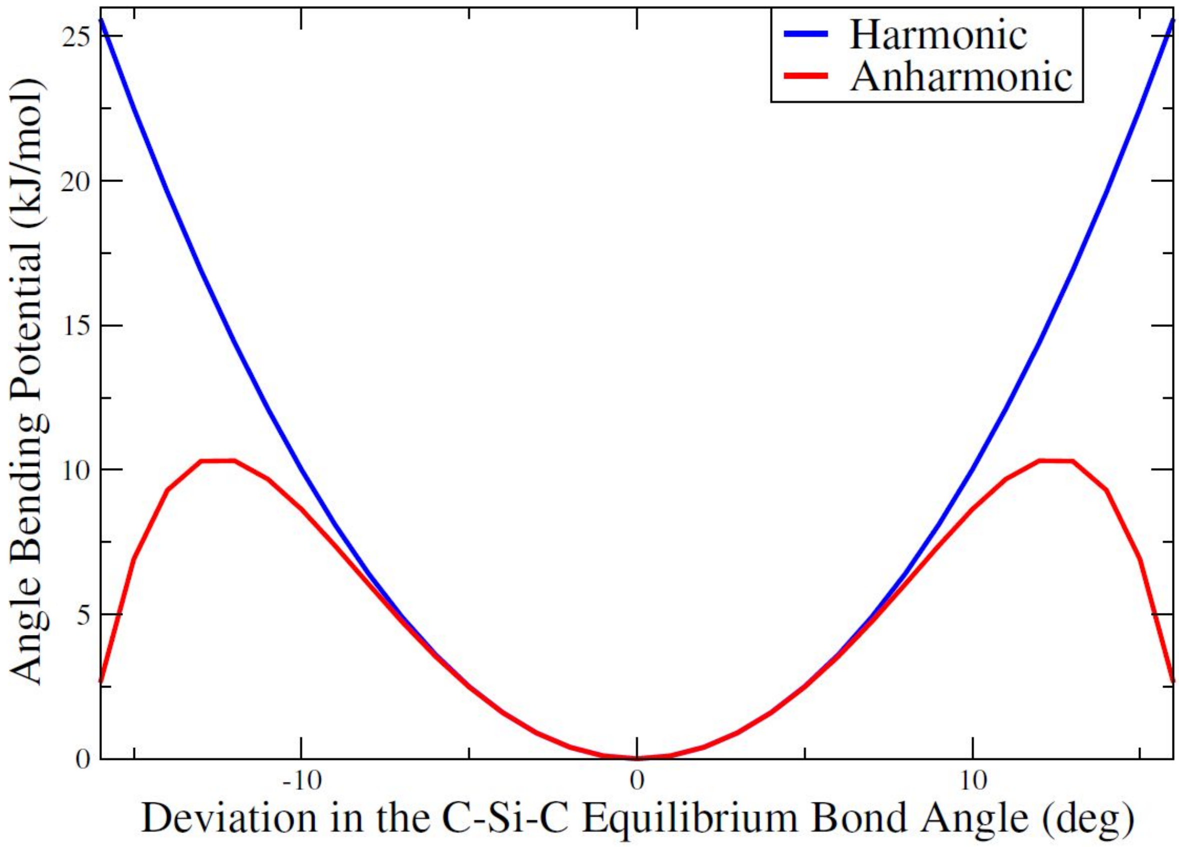

Figure S21 *– Comparison of the full anharmonic potential from Grigoras and Lane [46] (red line) with a reduced harmonic potential (blue line) for bending of the C–Si–C angle.*

S4. Dihedral Parameterization

Each set of torsion parameters was determined by fitting the Ryckaert-Bellemans expression, equation (9), to a dihedral scan computed from DFT calculations on selected molecules.

$$U_{\mathrm{Torsion}} = \sum_{i=0}^{5} C_i (\cos \phi)^i \#(9)$$

In this equation, U_{Torsion} is the torsional energy, ϕ is the dihedral angle, and C_{i} are the 6 fitting constants (in kJ/mol). We note that in the majority of cases, it was sufficient to fit the first 4 constants, with the last two (i.e. for i=4 and i=5) assumed to be equal to zero.

All DFT calculations made use of the B3LYP exchange-correlation functional [53, 54] and the aug-cc-pVTZ basis set [55], which includes diffuse functions. For each molecule, the atoms pertaining to the dihedral of interest were rotated incrementally in steps of 30º over the entire 360º range, leading to a total of 12 DFT calculations per dihedral. In each calculation, all the heavy atoms, as well as the hydrogen atoms belonging to hydroxyl groups (which are explicitly represented in our United-Atom force field) were kept fixed, while all the aliphatic hydrogen atom positions were optimized. This is in keeping with the philosophy of a UA model, where the aliphatic hydrogens are implicitly described through the parameter set for the adjacent carbon atom – i.e. each CH_x groups is considered as a single interaction site.

Once all the DFT energies were calculated, they were normalized by the energy at the minimum of the curve, so that the torsional energy profile was mostly positive (see examples below for details). It is then necessary to establish an equivalence between this normalized DFT energy and its counterpart in the classical model, which contains contributions from all potential energy terms according to equation (10):

$$U_{\mathrm{DFT}} = U_{\mathrm{Bond}} + U_{\mathrm{Angle}} + U_{\mathrm{Torsion}} + U_{\mathrm{LJ}} + U_{\mathrm{Coul}} \#(10)$$

where U_{Bond} is the bond stretching energy, U_{Angle} is the angle bending energy, U_{LJ} is the Lennard-Jones energy (including both dispersion and repulsion), and U_{Coul} is the Coulomb electrostatic energy due to point charges. It is important to note that because the DFT energy is normalized, i.e. it refers to a difference relative to the energy at the minimum, only those terms in equation (10) that vary over the dihedral scan need to be taken into account. In particular, the bond and angle terms were always kept constant over each scan, and so can be disregarded. We note also that because in our force field approach, no 1-4 LJ or Coulomb interactions were considered, only terms for atoms separated by 4 or more bonds need to be taken into account. To achieve this, the final DFT optimized geometries at each point of the scan were extracted and the distances between all relevant atoms were computed using a python script. Finally, the torsional energy contains contributions due to all dihedral angles that are changed during the scan (which were often more than one because of the tetrahedral coordination of Si atoms). The description below provides details about which terms were considered for each individual dihedral. The fitted parameters for each dihedral are provided in Table S72 at the end of this section.

CCSiC

This is the only dihedral term that needed parameterization to describe all tetraalkylsilane molecules considered here. For silanes with longer alkyl substituents (i.e. propyl and beyond), the TraPPE parameters for CCCC torsion can be adopted [56]. The CCSiC dihedral was fitted to a torsional scan on the M3E1Si molecule (i.e. trimethylethylsilane), which was the simplest molecule that contained this dihedral term (see Figure S22).

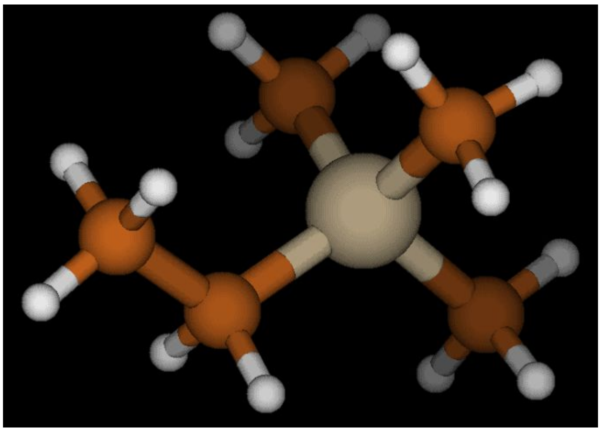

Figure S22 *– Ball-and-stick representation of the M3E1Si molecule, used to parameterize the CCSiC dihedral. Carbon atoms are shown in brown, Silicon in cream, and Hydrogens in white.*

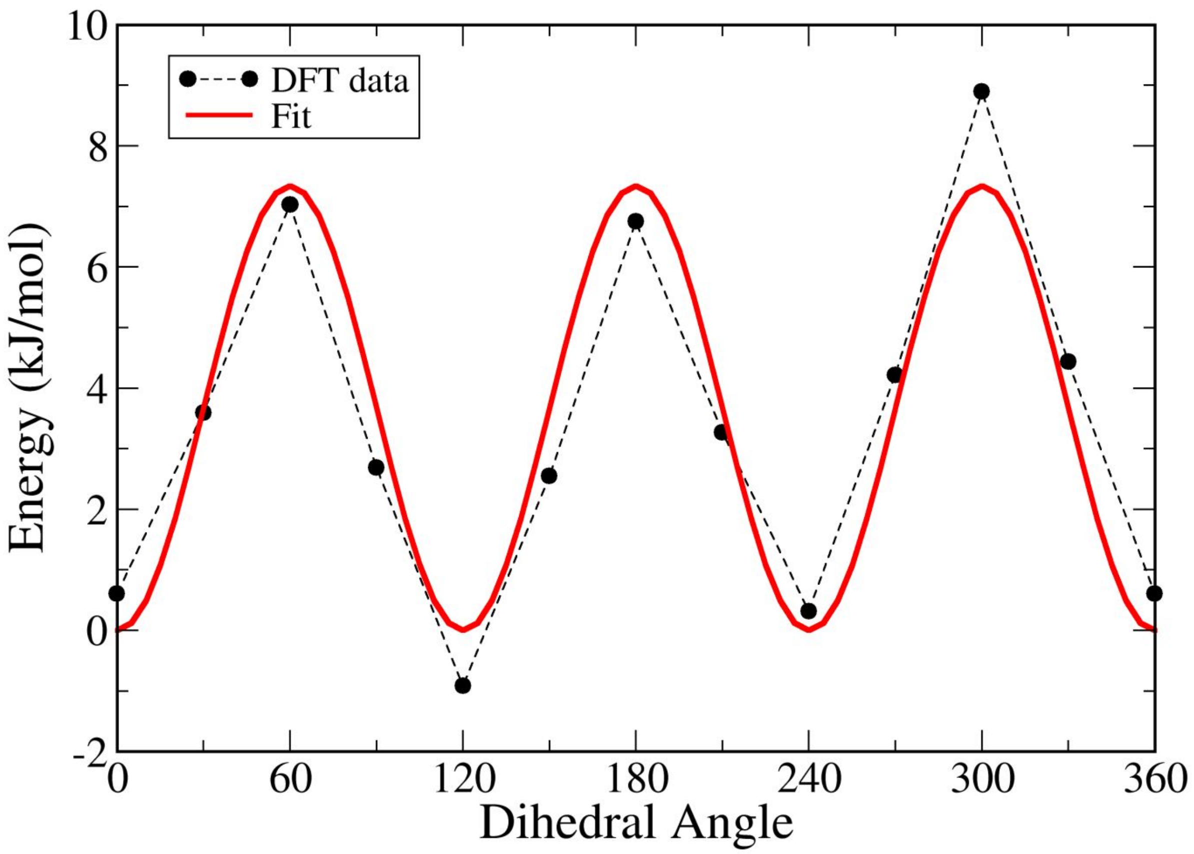

Figure S23 *– Comparison between the DFT energy profile (black circles) and the classical torsion potential (red line) for the CCSiC dihedral. The black dashed line is a guide to the eye.*

Because the in the UA approach, none of the aliphatic hydrogens are explicitly considered, there were no LJ or Coulomb interactions to consider. Therefore, the total DFT energy scan contains only contributions from the three CCSiC dihedrals in this molecule (each of them offset by ±120º from the others). In Figure S23, we show the normalized (total) DFT energy together with the sum of the three classical torsion contributions, which were fitted to match the DFT profile. We note that in the normalization of the DFT energy, the average energy over the three minima was taken as the reference point to ensure a balanced fit was obtained. As we can see, the classical potential provides a good description of the DFT energy profile.

$CSiO_CC$

The next set of dihedrals were necessary to describe alkoxysilane molecules. The $CSiO_CC$ dihedral was parameterized by fitting against DFT data obtained on the M3SiOM molecule (i.e. trimethylmethoxysilane), shown in Figure S24. In this molecule, there are no 1-5 interactions, so no LJ or Coulomb contributions had to be considered. Therefore, the total DFT energy scan contains only contributions from the three $CSiO_CC$ dihedrals in this molecule (each of them offset by ±120º from the others). In Figure S25, we show the normalized total DFT energy together with the sum of the three classical torsion contributions, which were fitted to match the DFT profile. Once again, in the normalization of the DFT energy, the average energy over the three minima was taken as the reference point to ensure a balanced fit was obtained.

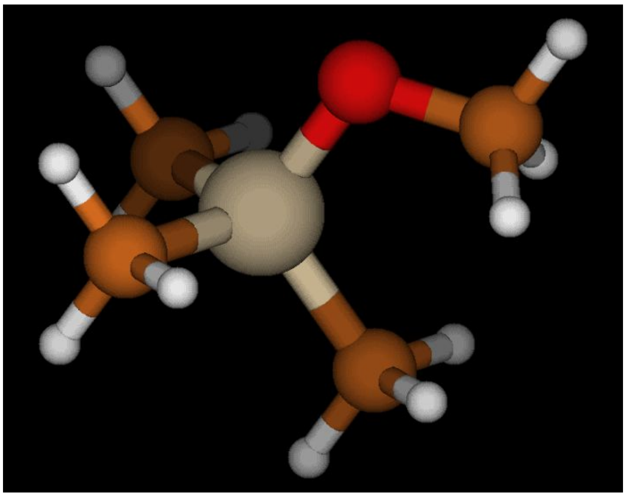

Figure S24 *– Ball-and-stick representation of the M3SiOM molecule, used to parameterize the* $CSiO_CC$ *dihedral. Carbon atoms are shown in brown, Silicon in cream, Oxygens in red, and Hydrogens in white.*

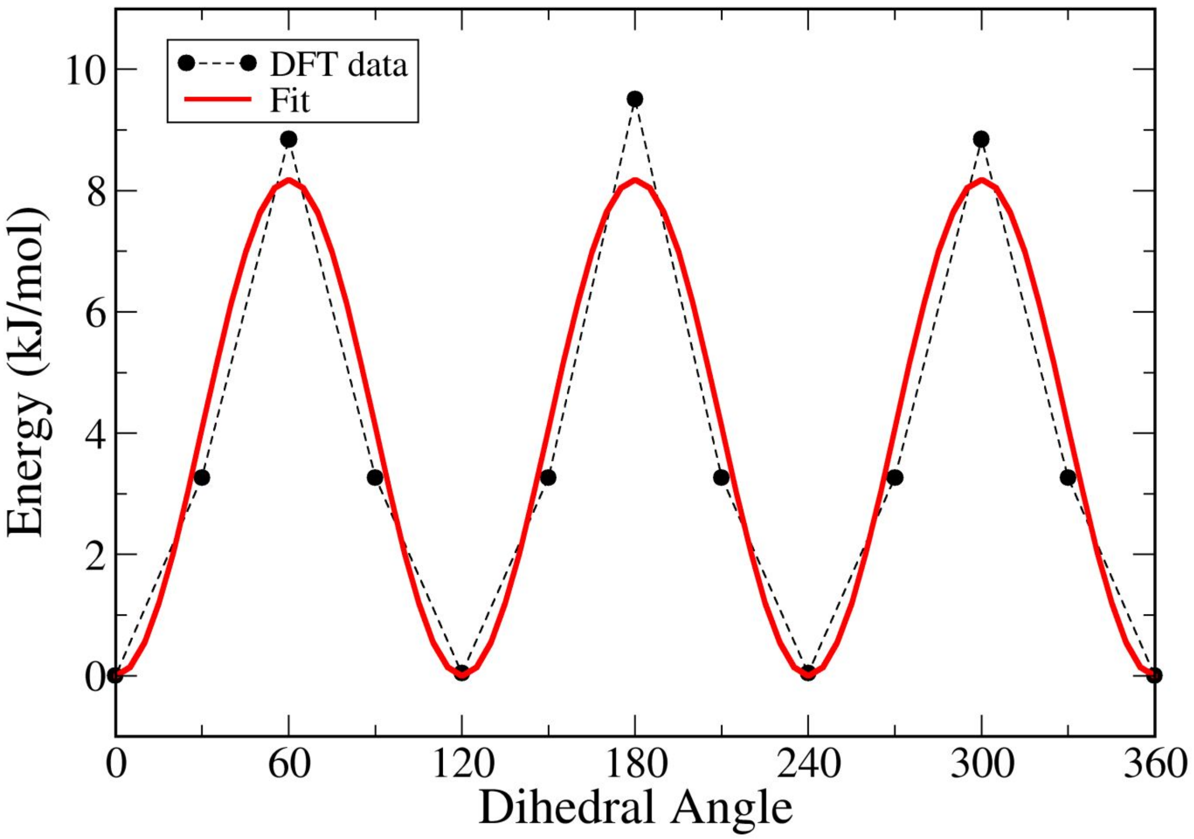

Figure S25 *– Comparison between the DFT energy profile (black circles) and the classical torsion potential (red line) for the CSiO$_C$C dihedral. The black dashed line is a guide to the eye.*

CCSiO$_C$

The CCSiO$_C$ dihedral was parameterized by fitting against DFT data obtained on the M2ESiOM molecule (i.e. dimethylethylmethoxysilane), shown in Figure S26, which was the simplest molecule containing the dihedral in question. In this molecule, there are 1-5 interactions between the terminal carbons in the ethyl and methoxy groups. However, in our UA approach, aliphatic CH_x groups that are not directly bonded to a polar functional group are assigned a charge of zero (see also section S2). Therefore, the Coulomb contributions were zero. Apart from the 1-5 LJ terms, the total DFT energy scan contains contributions from the target CCSiO$_C$ dihedral, but also from two CCSiC dihedrals (see Figure S26), which thus had to be subtracted from the energy profile. In Figure S27, we show the normalized DFT energy of the CCSiO$_C$ dihedral together with the corresponding classical torsion potential, which was fitted to match the DFT profile.

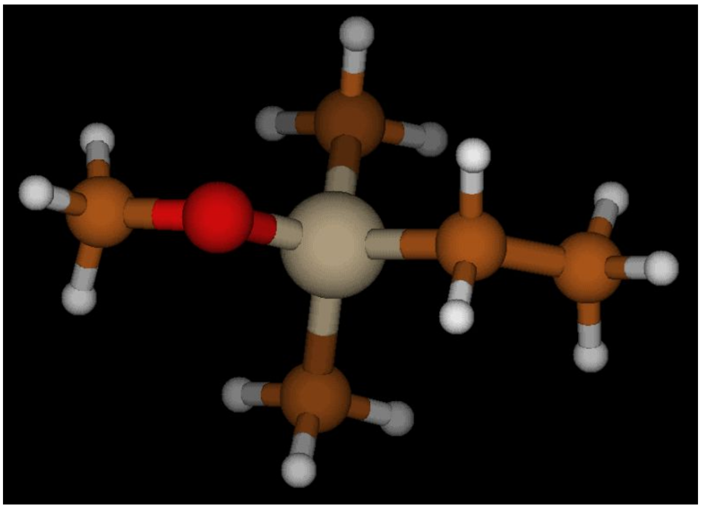

Figure S26 *– Ball-and-stick representation of the M2ESiOM molecule, used to parameterize the $CCSiO_C$ dihedral. Color code is the same as in Figure S24.*

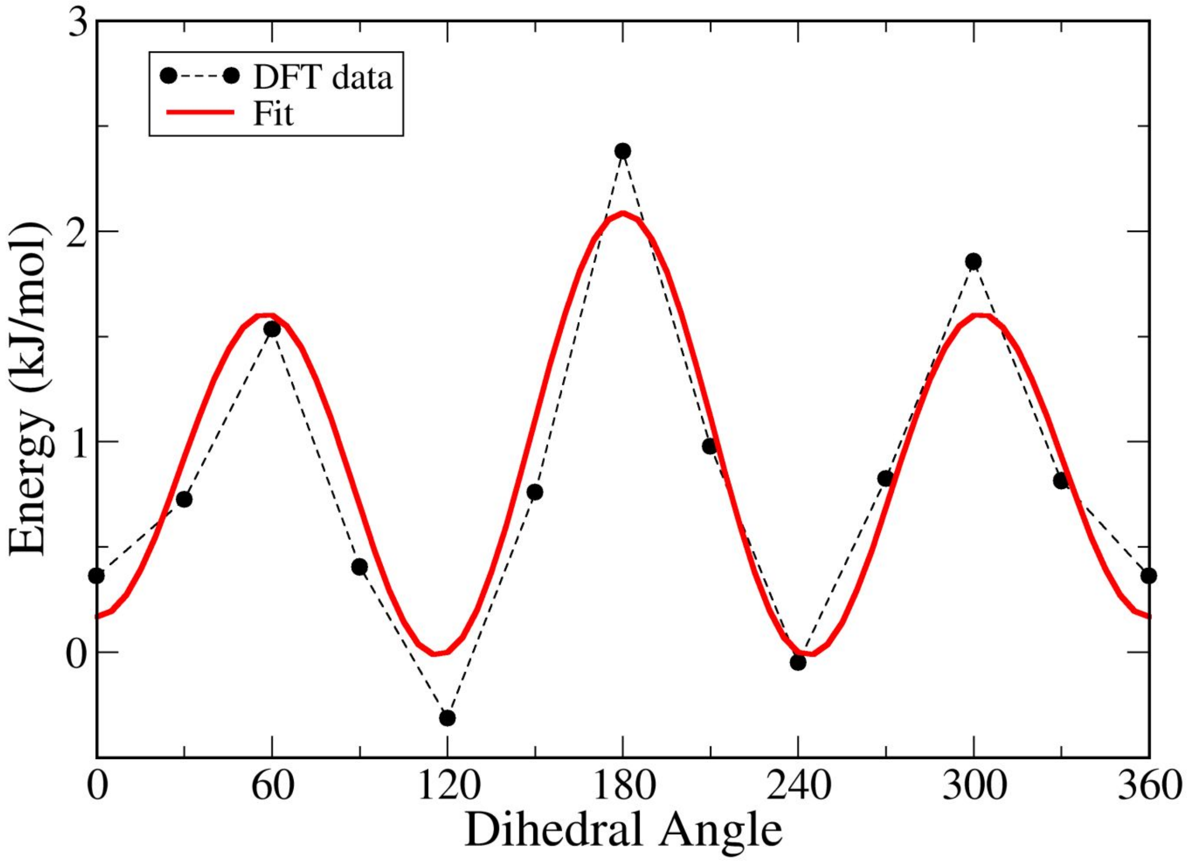

Figure S27 *– Comparison between the DFT energy profile (black circles) and the classical torsion potential (red line) for the $CCSiO_C$ dihedral. The black dashed line is a guide to the eye.*

<u>CCO_CSi</u>

Parameters for the CCO_CSi dihedral were taken from the TraPPE force field for ethers [57], assuming that the terminal Si atom had the same effect as a terminal C atom with the same tetrahedral bonding.

CO_CSiO_C

The CO_CSiO_C dihedral was parameterized by fitting against DFT data obtained on the M2SiOM2 molecule (i.e. dimethyldimethoxysilane), shown in Figure S28. In this molecule, there are 1-5 interactions between the terminal carbons in both methoxy groups. Since both of these atoms are bonded to an oxygen atom, they are charged (see Table S73), and therefore both LJ and Coulomb contributions were non-zero. Apart from the 1-5 LJ terms, the total DFT energy scan also contains contributions from two $CSiO_CC$ dihedrals (see Figure S28), which were subtracted from the energy profile, having been previously parameterized. In Figure S29, we show the normalized DFT energy of the CO_CSiO_C dihedral together with the corresponding classical torsion potential, which was fitted to match the DFT profile.

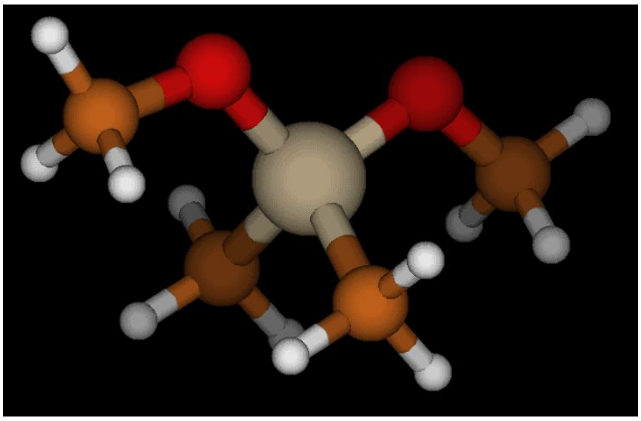

Figure S28 *– Ball-and-stick representation of the M2SiOM2 molecule, used to parameterize the CO_CSiO_C dihedral. Color code is the same as in Figure S24.*

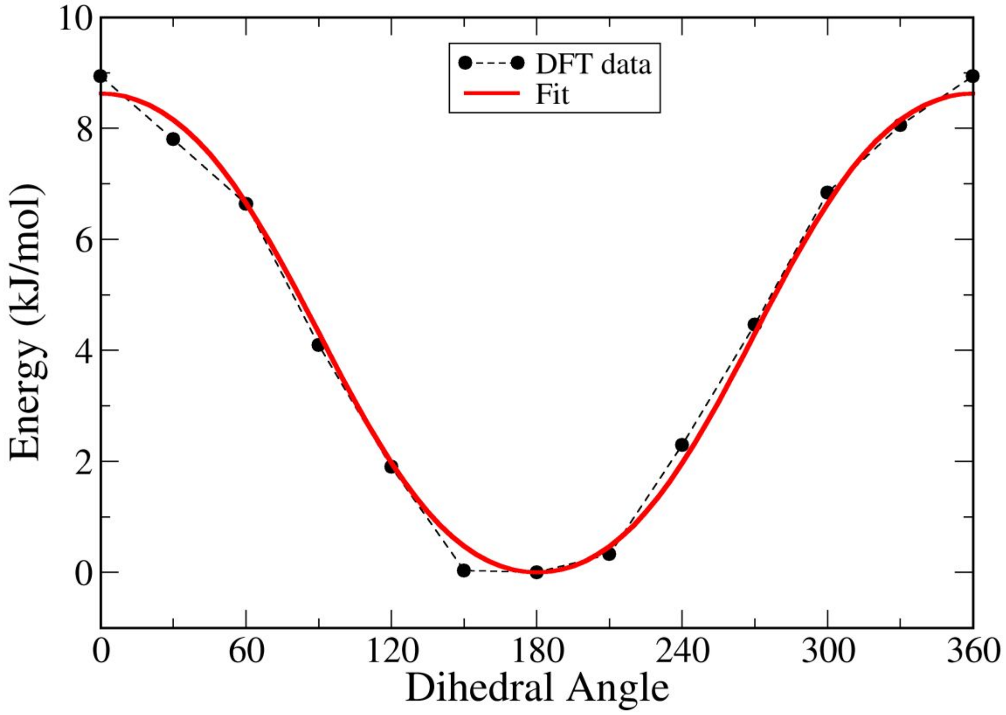

Figure S29 *– Comparison between the DFT energy profile (black circles) and the classical torsion potential (red line) for the CO_CSiO_C dihedral. The black dashed line is a guide to the eye.*

$CSiO_HH$

This dihedral and the next one were necessary to describe trialkylsilanol molecules. The $CSiO_HH$ dihedral was parameterized by fitting against DFT data obtained on the M3SiOH molecule (i.e. trimethylsilanol), shown in Figure S30. There are no 1-5 interactions in this molecule, and so the DFT profile contains only contributions from the three identical dihedrals (each offset by ±120º). Figure S31 shows the total normalized DFT energy together with the corresponding fit to the classical torsion potential. We note that for this dihedral, a more detailed DFT scan, with a spacing of 10º, was carried out.

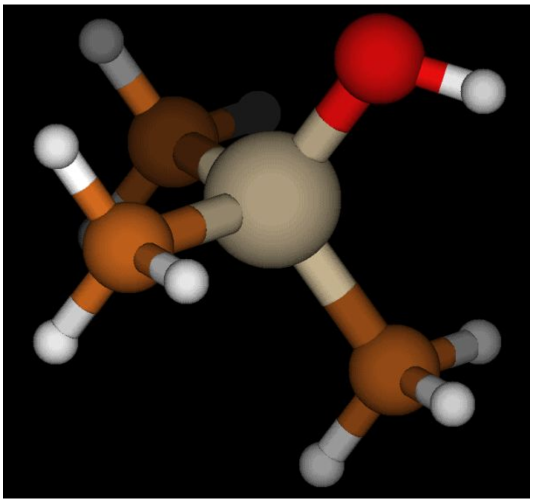

Figure S30 *– Ball-and-stick representation of the M3SiOH molecule, used to parameterize the* $CSiO_HH$ *dihedral. Color code is the same as in Figure S24.*

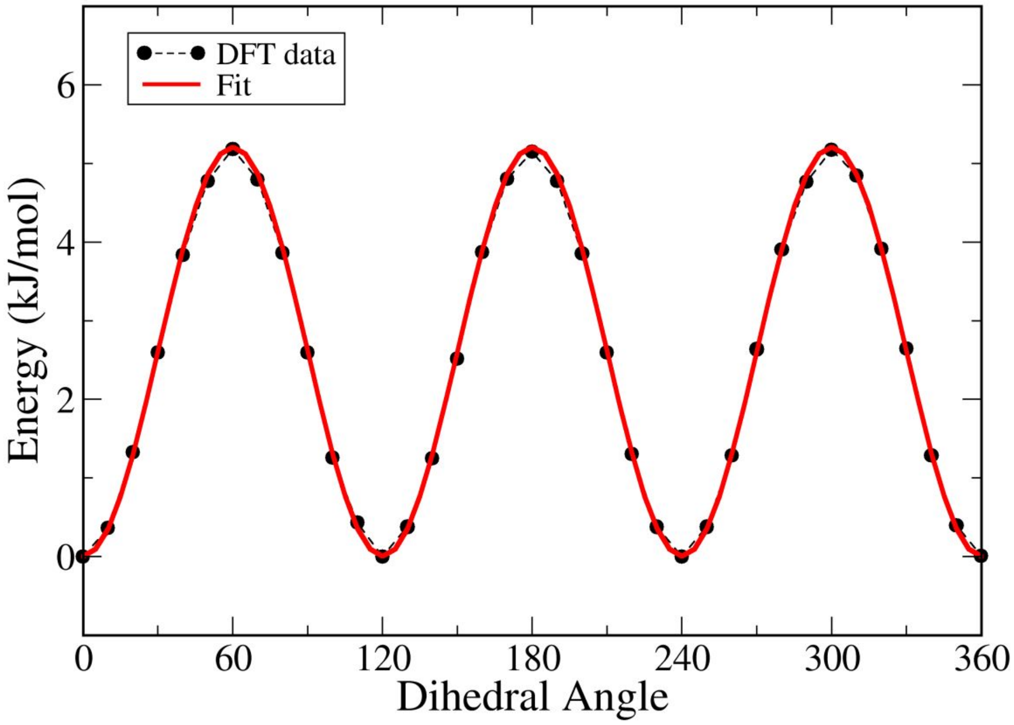

Figure S31 *– Comparison between the DFT energy profile (black circles) and the classical torsion potential (red line) for the* $CSiO_HH$ *dihedral. The black dashed line is a guide to the eye.*

$CCSiO_H$

The $CCSiO_H$ dihedral was parameterized by fitting against DFT data obtained on the M2ESiOH molecule (i.e. dimethylethylsilanol), shown in Figure S32. There are 1-5 interactions between the hydroxyl hydrogen atom and the terminal carbon atom of the ethyl group. However, the terminal carbon is uncharged, while the hydroxyl hydrogen has no LJ interactions associated with it; hence, both LJ and Coulomb contributions are effectively zero in this molecule. The DFT energy contains contributions from two CCSiC dihedrals (see Figure S32), which were subtracted from the profile. Figure S33 shows the normalized DFT energy for the target $CCSiO_H$ dihedral, together with the corresponding fit to the classical torsion potential.

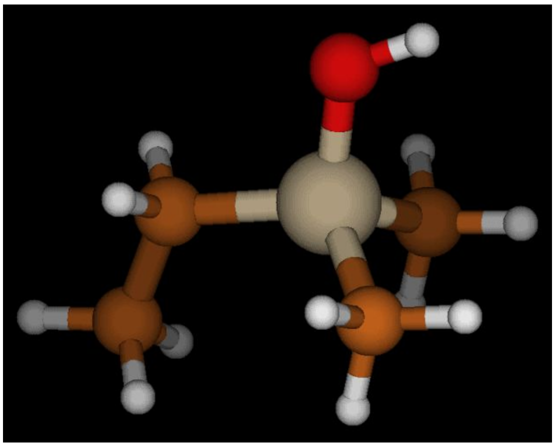

Figure S32 *– Ball-and-stick representation of the M3SiOH molecule, used to parameterize the $CCSiO_H$ dihedral. Color code is the same as in Figure S24.*

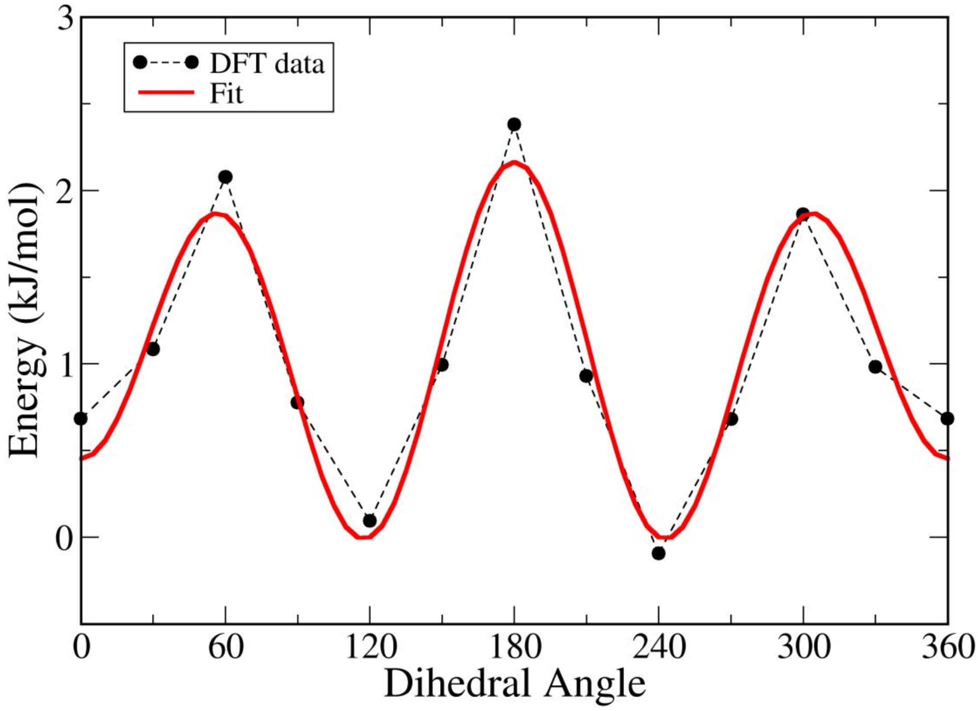

Figure S33 *– Comparison between the DFT energy profile (black circles) and the classical torsion potential (red line) for the $CCSiO_H$ dihedral. The black dashed line is a guide to the eye.*

O_CSiO_HH

This dihedral and the next one were necessary to describe molecules with both hydroxyl and alkoxy substituents. The O_CSiO_HH dihedral was parameterized by fitting against DFT data obtained on the M2SiOMOH molecule (i.e. dimethylmethoxysilanol), shown in Figure S34. There are 1-5 interactions between the hydroxyl hydrogen atom and the carbon atom of the methoxy group. However, the hydroxyl hydrogen has no LJ interactions associated with it, so only Coulomb contributions had to be calculated. The DFT energy also contains contributions from two $CSiO_HH$ dihedrals, which were subtracted from the profile, having been previously parameterized. Figure S35 shows the normalized DFT energy for the target O_CSiO_HH dihedral, together with the corresponding fit to the classical torsion potential.

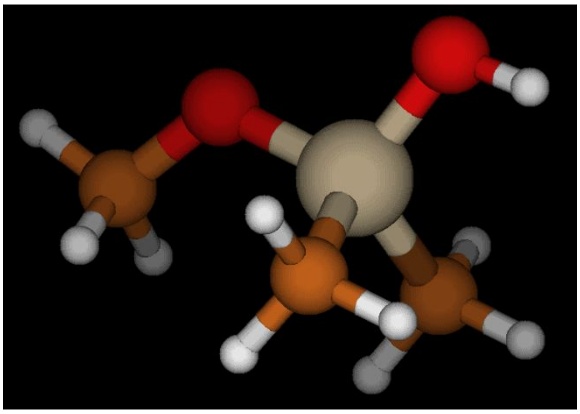

Figure S34 *– Ball-and-stick representation of the M2SiOMOH molecule, used to parameterize the O_CSiO_HH and CO_CSiO_H dihedrals. Color code is the same as in Figure S24.*

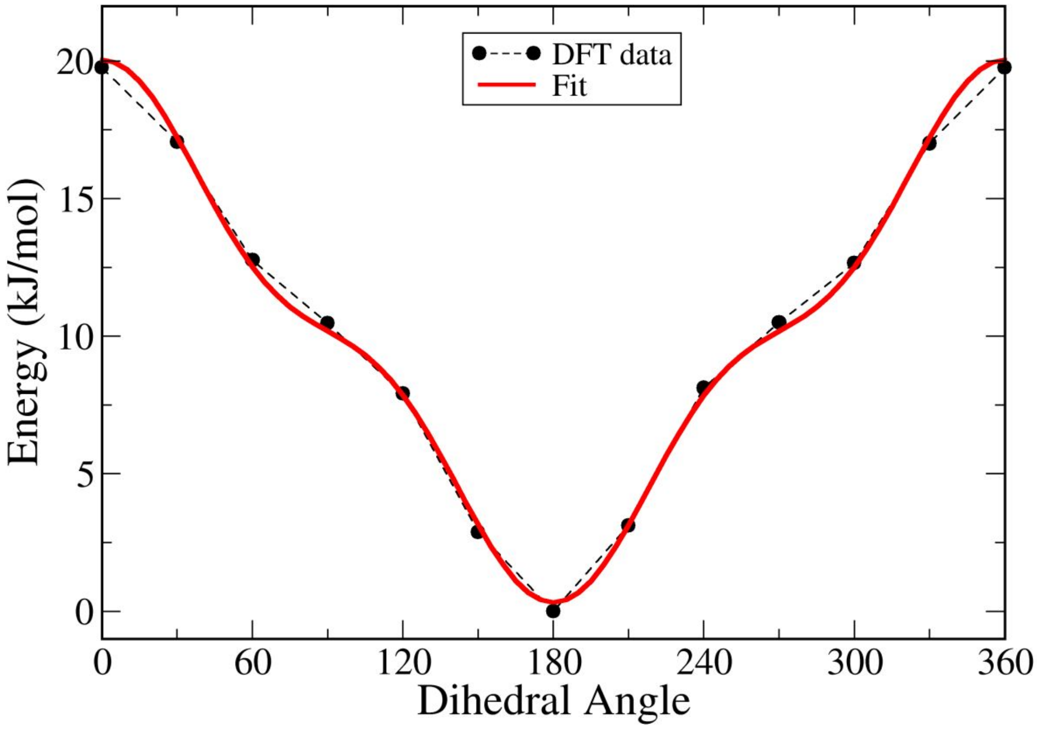

Figure S35 *– Comparison between the DFT energy profile (black circles) and the classical torsion potential (red line) for the O_CSiO_HH dihedral. The black dashed line is a guide to the eye.*

CO_CSiO_H

The M2SiOMOH molecule (Figure S34) was also used to parameterize the CO_CSiO_H dihedral. Once again, there are 1-5 Coulomb interactions between the hydroxyl hydrogen atom and the carbon atom of the methoxy group, since the LJ contribution is zero. The DFT energy for this scan also contains contributions from two $CSiO_CC$ dihedrals, which were subtracted from the profile. Figure S36 shows the normalized DFT energy for the target CO_CSiO_H dihedral, together with the corresponding fit to the classical torsion potential.

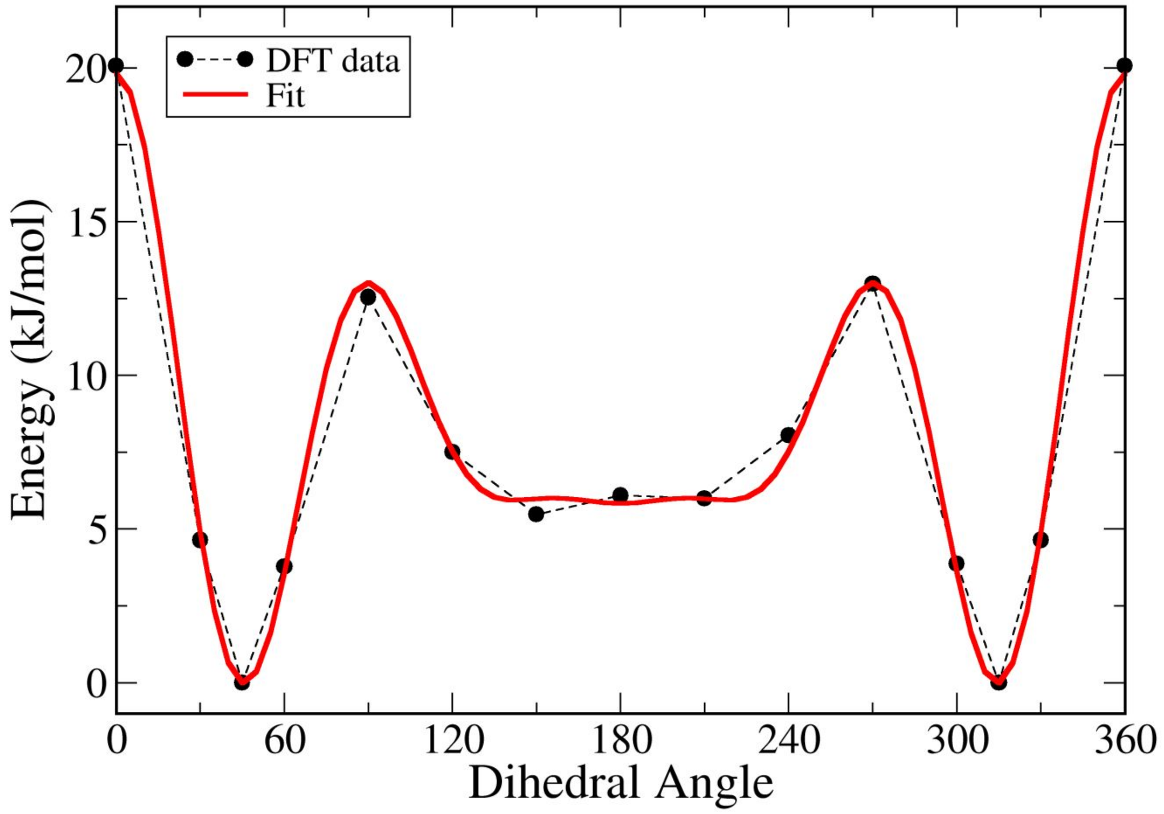

Figure S36 *– Comparison between the DFT energy profile (black circles) and the classical torsion potential (red line) for the CO_CSiO_H dihedral. The black dashed line is a guide to the eye.*

O_HSiO_HH

This dihedral is necessary to describe molecules with more than one hydroxyl substituent, including silicic acid. The O_HSiO_HH dihedral was parameterized by fitting against DFT data obtained on the M2SiOH2 molecule (i.e. dimethylsilanediol), shown in Figure S37. There are 1-5 Coulomb interactions to consider between both hydroxyl hydrogen atoms. The DFT energy also contains contributions from two $CSiO_HH$ dihedrals, which were subtracted from the profile. Figure S38 shows the normalized DFT energy for the target O_HSiO_HH dihedral, together with the corresponding fit to the classical torsion potential.

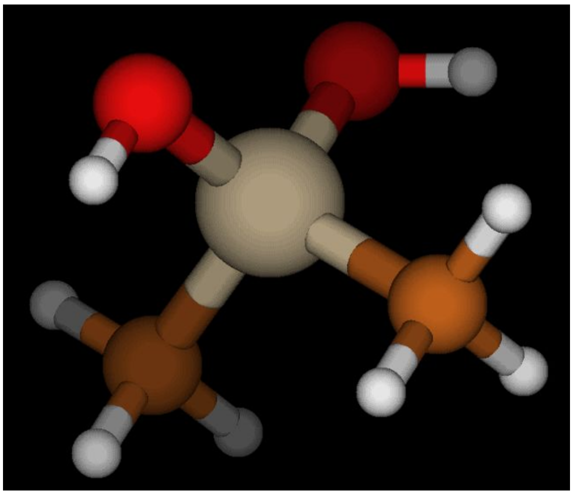

Figure S37 *– Ball-and-stick representation of the M2SiOH2 molecule, used to parameterize the* O_HSiO_HH *dihedral. Color code is the same as in Figure S24.*

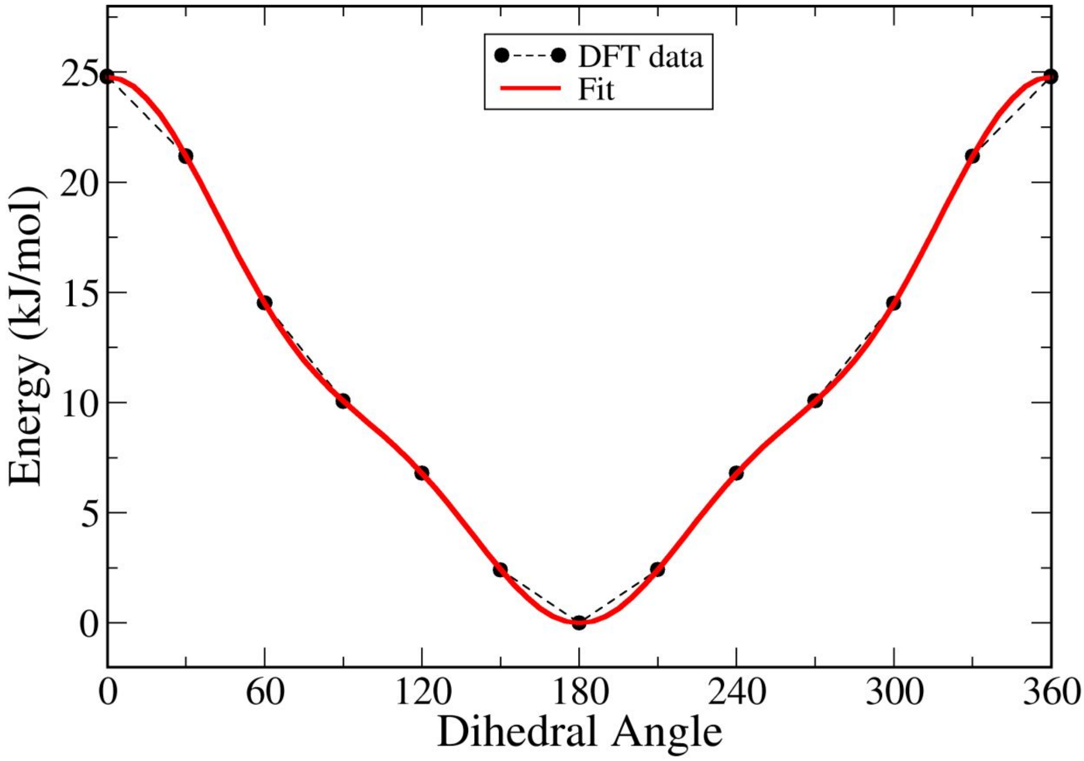

Figure S38 *– Comparison between the DFT energy profile (black circles) and the classical torsion potential (red line) for the* O_HSiO_HH *dihedral. The black dashed line is a guide to the eye.*

We also carried out a validation step for this dihedral, on the MSiOH3 molecule (i.e., methylsilanetriol), shown in Figure S39. Figure S40 shows the normalized DFT energy scan for this molecule, after subtracting the Coulomb contributions for the interactions between hydroxyl hydrogen atoms. In red, we show the predicted energy profile obtained by adding together the contributions of the two O_HSiO_HH and one $CSiO_HH$ dihedral, which were previously parameterized. As we can see, the shape of the profile is correctly predicted;

although the energy basin is slightly wider for the classical potential, the height of the energy barrier is predicted with great accuracy.

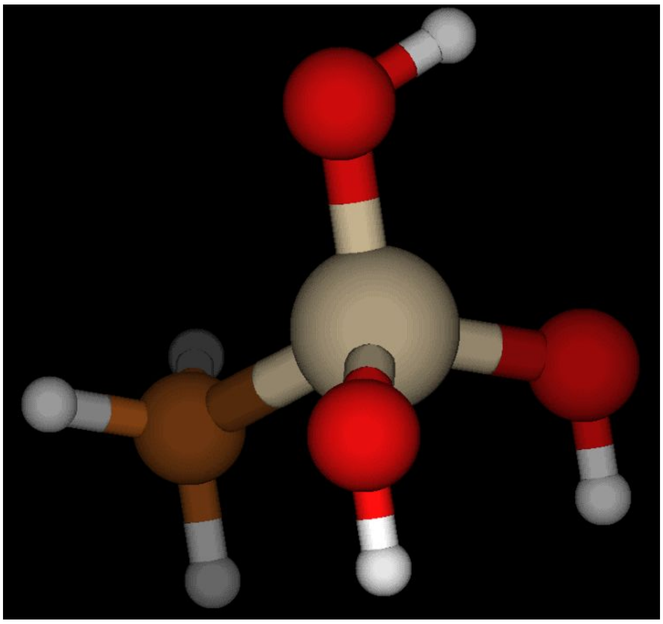

Figure S39 *– Ball-and-stick representation of the MSiOH3 molecule, used to validate the* O_HSiO_HH *dihedral. Color code is the same as in Figure S24.*

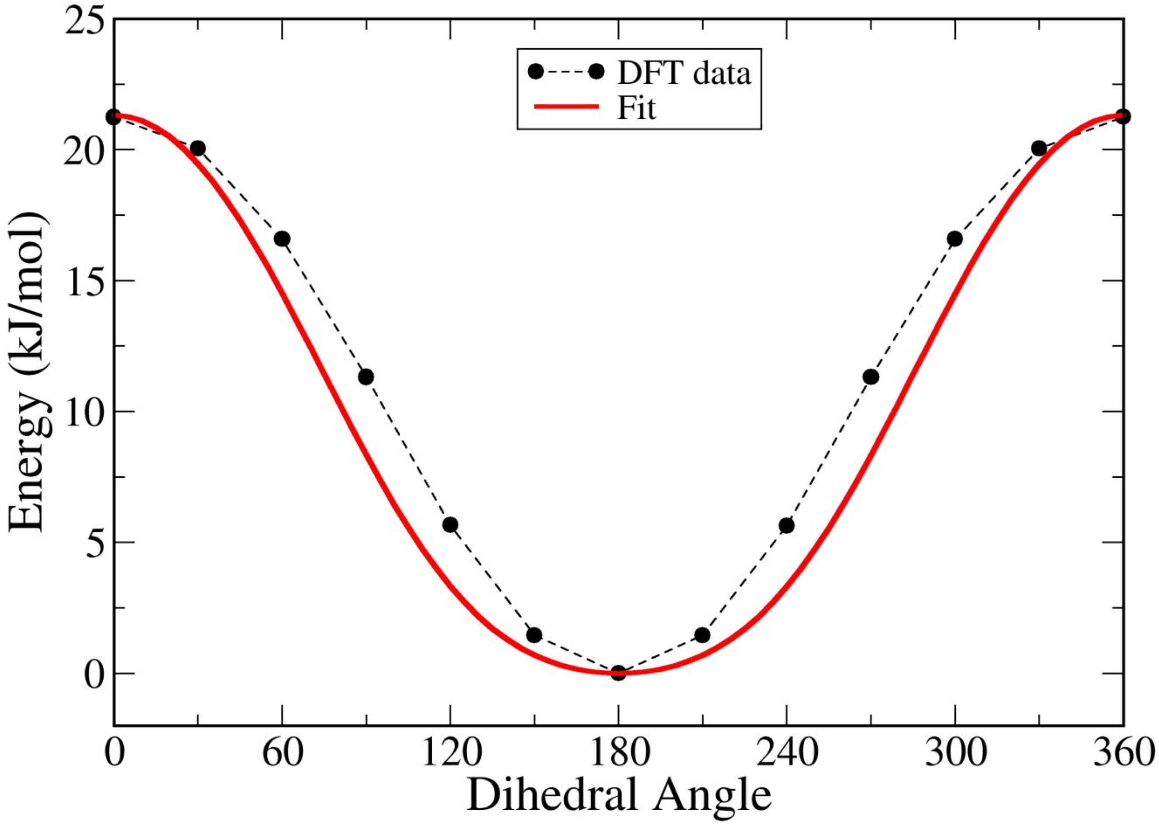

Figure S40 *– Comparison between the DFT energy profile (black circles) and the classical torsion potential (red line) for the three dihedrals of the MSiOH3 molecule. In this case, the red line is a prediction, the dihedrals having been previously fitted to DFT scan for different molecules. The black dashed line is a guide to the eye.*

$CCSiO_B$

This final set of dihedrals are those that include bridging oxygen atoms, and therefore apply to molecules with more than one Si atom. For simplicity, bridging oxygens at the edge of a dihedral were assumed to be equivalent to alkoxide oxygens. Therefore, the parameters for the $CCSiO_B$ dihedral were assumed to be equal to those of the previously parameterized $CCSiO_C$ dihedral. The assumption that the bonding environment of the terminal oxygen atom has very little effect on the dihedral potential is validated by the similarity between the energy profiles for the $CCSiO_C$ and $CCSiO_H$ dihedrals (compare Figures S27 and S33).

CO_CSiO_B

As explained above, the parameters for the CO_CSiO_B dihedral were assumed to be equal to those of the previously parameterized CO_CSiO_C dihedral.

$CSiO_BSi$

This dihedral was parameterized by fitting against DFT data obtained on the M3SiOSiH3 molecule (i.e. 1,1,1-trimethyldisiloxane), shown in Figure S41. There are no 1-5 interactions to consider for this molecule. Figure S42 shows the total normalized DFT energy for the three identical $CSiO_BSi$ dihedrals, together with the corresponding fit to the classical torsion potential. We note the very low magnitude of the energy barriers, indicating that this is nearly a freely rotating dihedral.

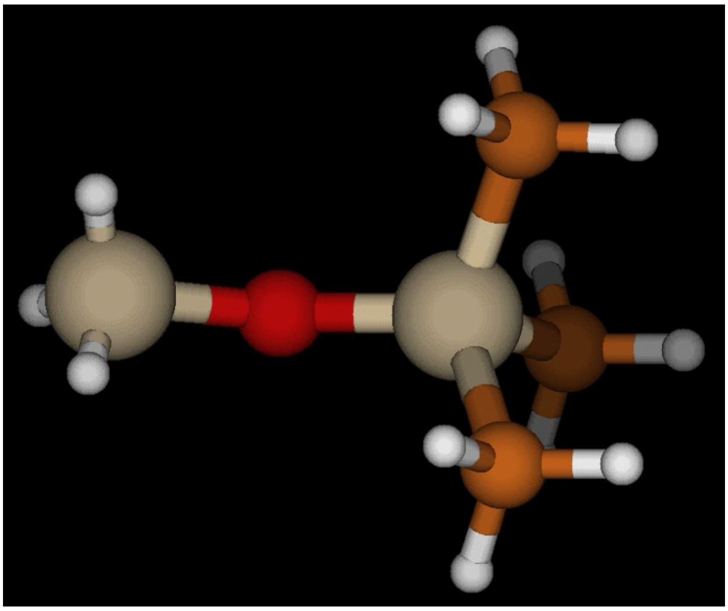

Figure S41 *– Ball-and-stick representation of the M3SiOSiH3 molecule, used to parameterize the* $CSiO_BSi$ *dihedral. Color code is the same as in Figure S24.*

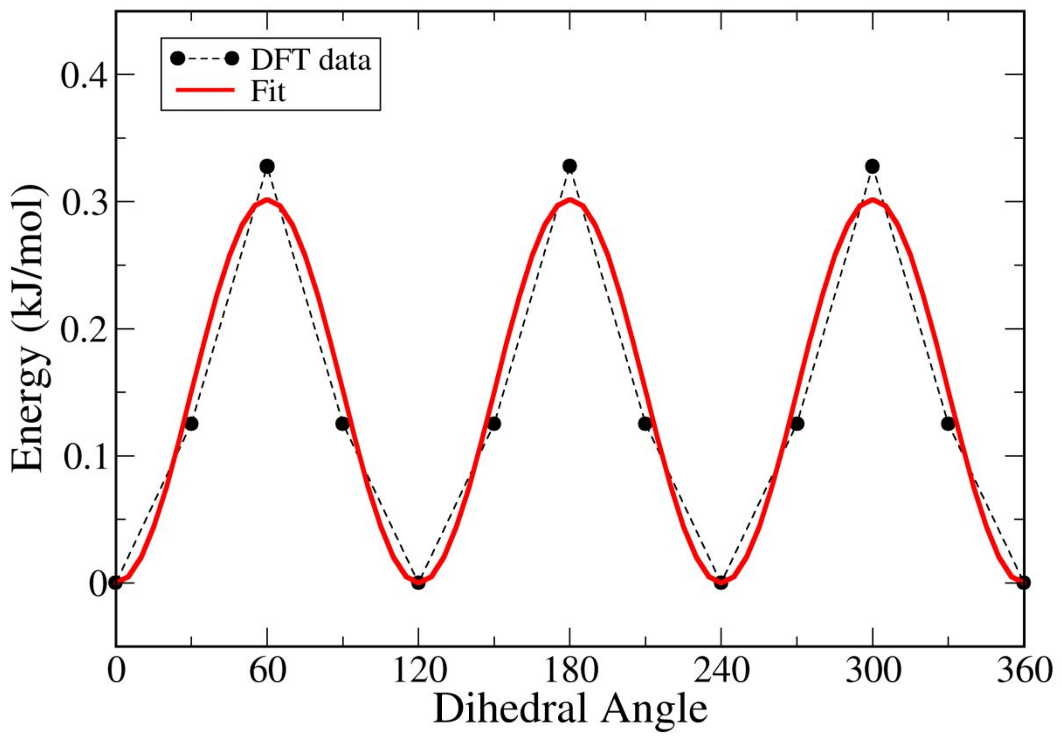

Figure S42 *– Comparison between the DFT energy profile (black circles) and the classical torsion potential (red line) for the $CSiO_BSi$ dihedral. The black dashed line is a guide to the eye.*

O_CSiO_BSi

This dihedral was parameterized by fitting against DFT data obtained on the M2OMSiOSiH3 molecule (i.e. 1,1-dimethyl-1-methoxydisiloxane), shown in Figure S43. There are LJ and Coulomb 1-5 interactions to consider for this molecule, between the carbon atom of the methoxy group and the terminal Si atom. For the latter, a charge of +1.34 was considered, obtained from a DFT calculation on this molecule. The DFT energy also contains contributions from two $CSiO_BSi$ dihedrals, which were subtracted from the profile. Figure S44 shows the normalized DFT energy for the target O_CSiO_BSi dihedral, together with the corresponding fit to the classical torsion potential.

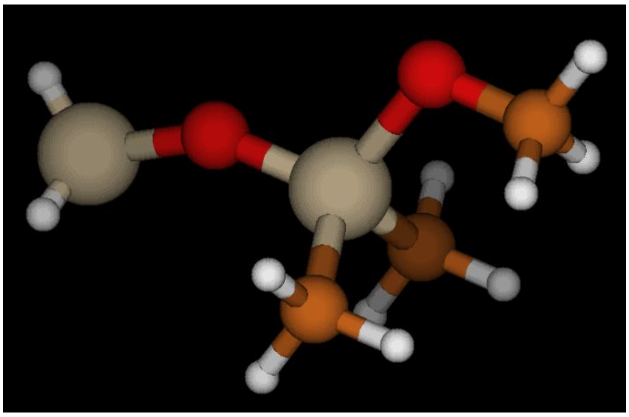

Figure S43 *– Ball-and-stick representation of the M2OMSiOSiH3 molecule, used to parameterize the O_CSiO_BSi dihedral. Color code is the same as in Figure S24.*

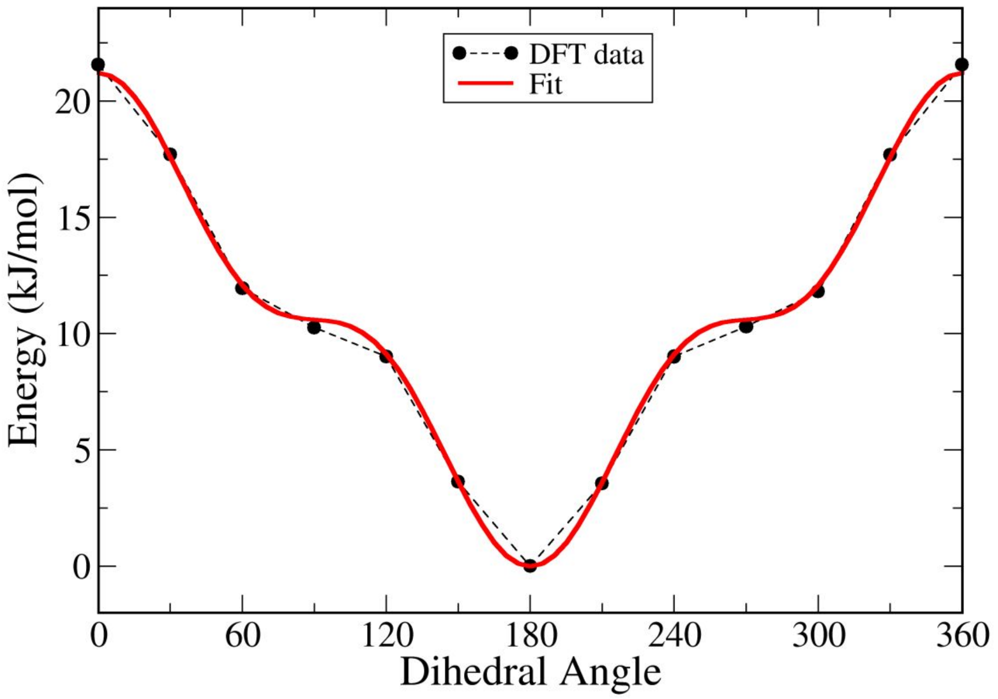

***Figure S44** – Comparison between the DFT energy profile (black circles) and the classical torsion potential (red line) for the O_CSiO$_B$Si dihedral. The black dashed line is a guide to the eye.*

O_HSiO$_B$Si

This dihedral was parameterized by fitting against DFT data obtained on the M2OMSiOSiH3 molecule (i.e. 1,1-dimethyl-1-methoxydisiloxane), shown in Figure S45. There are only Coulomb 1-5 interactions to consider between the hydroxyl hydrogen atom and the terminal Si atom – LJ interactions are zero because the former has no LJ site. A charge of +1.34 was considered for the Si atom. The DFT energy also contains contributions from two CSiO$_B$Si dihedrals, which were subtracted from the profile. Figure S46 shows the normalized DFT energy for the target O_HSiO$_B$Si dihedral, together with the corresponding fit to the classical torsion potential.

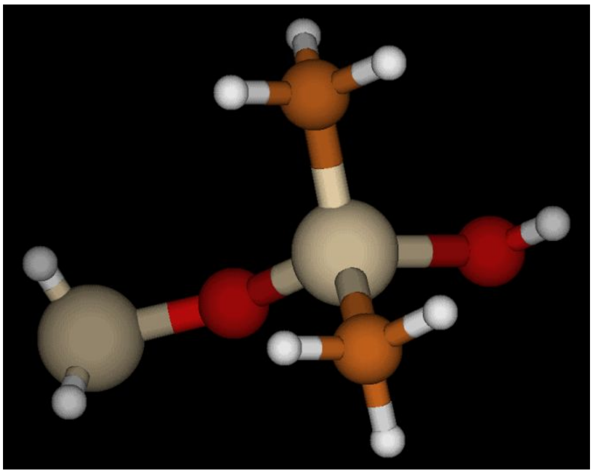

***Figure S45** – Ball-and-stick representation of the M2OHSiOSiH3 molecule, used to parameterize the O_HSiO$_B$Si dihedral. Color code is the same as in Figure S24.*

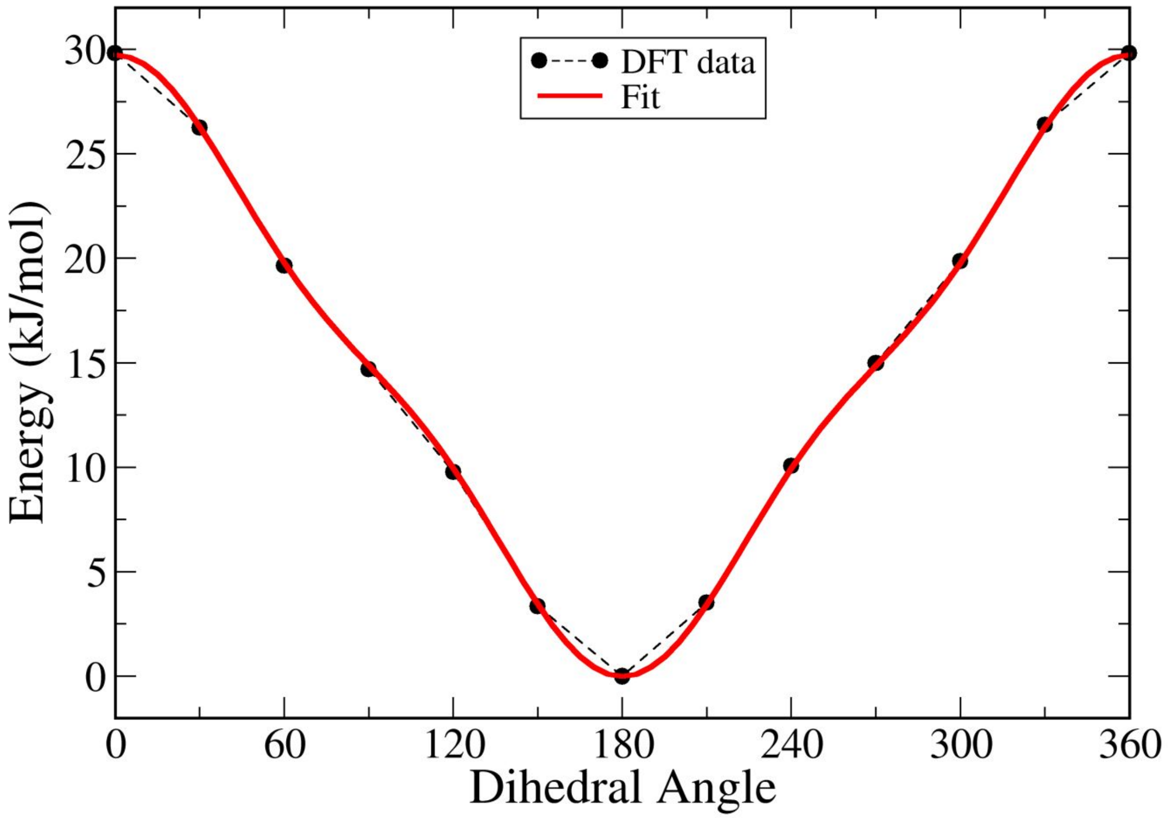

Figure S46 *– Comparison between the DFT energy profile (black circles) and the classical torsion potential (red line) for the O_HSiO_BSi dihedral. The black dashed line is a guide to the eye.*

Table S72 *– Final set of torsion parameters (in kJ/mol) for all dihedrals considered in this work.*

**Dihedral**	$k_{T,0}$	$k_{T,1}$	$k_{T,2}$	$k_{T,3}$	$k_{T,4}$	$k_{T,5}$	**Comment**
CCSiC	1.224	3.672	0.0	-4.895	0.0	0.0	
$CSiO_CC$	1.364	4.093	0.0	-5.457	0.0	0.0	
$CCSiO_C$	0.692	2.456	0.437	-3.416	0.0	0.0	
CCO_CSi	7.949	7.892	2.723	-18.563	0.0	0.0	Taken from TraPPE for CCO_CSi
CO_CSiO_C	4.314	4.803	0.0	-0.489	0.0	0.0	
$CSiO_HH$	0.870	2.600	0.0	-3.470	0.0	0.0	
$CCSiO_H$	0.801	2.760	0.508	-3.615	0.0	0.0	
O_CSiO_HH	10.189	2.939	0.0	6.918	0.0	0.0	
CO_CSiO_H	13.021	0.350	-39.801	-25.132	39.605	31.769	
O_HSiO_HH	10.071	6.167	2.322	6.236	0.0	0.0	
$CCSiO_B$	0.692	2.456	0.437	-3.416	0.0	0.0	Assumed equal to $CCSiO_C$
CO_CSiO_B	4.314	4.803	0.0	-0.489	0.0	0.0	Assumed equal to CO_CSiO_C
$CSiO_BSi$	0.0503	0.151	0.0	-0.201	0.0	0.0	
O_CSiO_BSi	10.607	0.420	0.0	10.187	0.0	0.0	
O_BSiO_BSi	10.607	0.420	0.0	10.187	0.0	0.0	Assumed equal to O_CSiO_BSi
O_HSiO_BSi	14.871	8.170	0.0	6.700	0.0	0.0	

S5. Additional Results

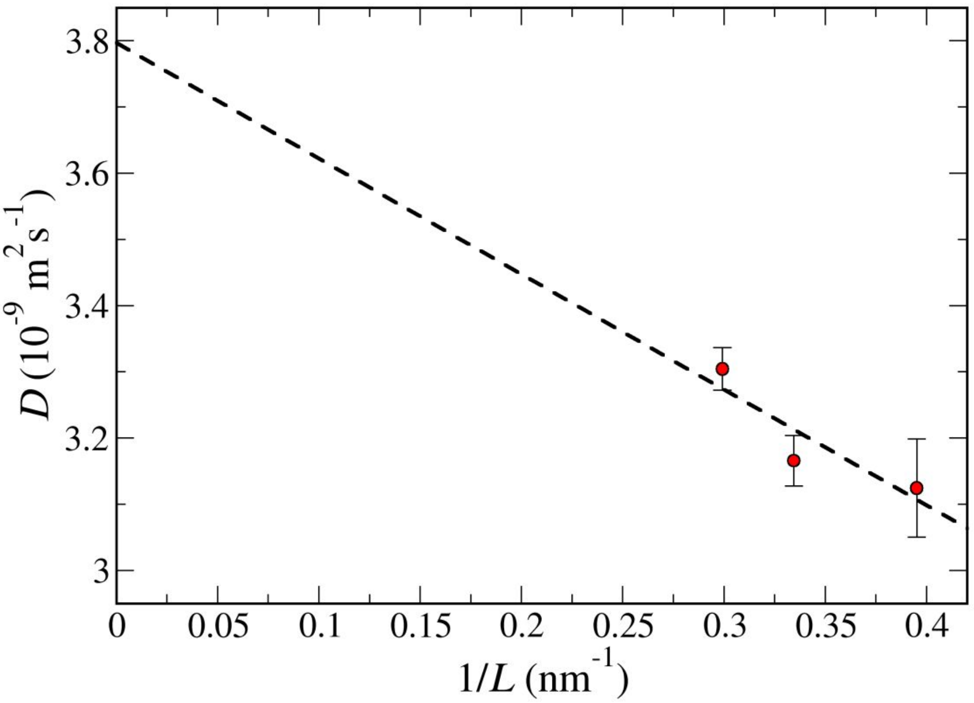

Figure S47 *– Plot of the self-diffusion coefficient of tetramethylsilane as a function of the inverse simulation box length. The data was fitted to a straight line and extrapolated to infinite box size to estimate a finite-size-corrected value.*

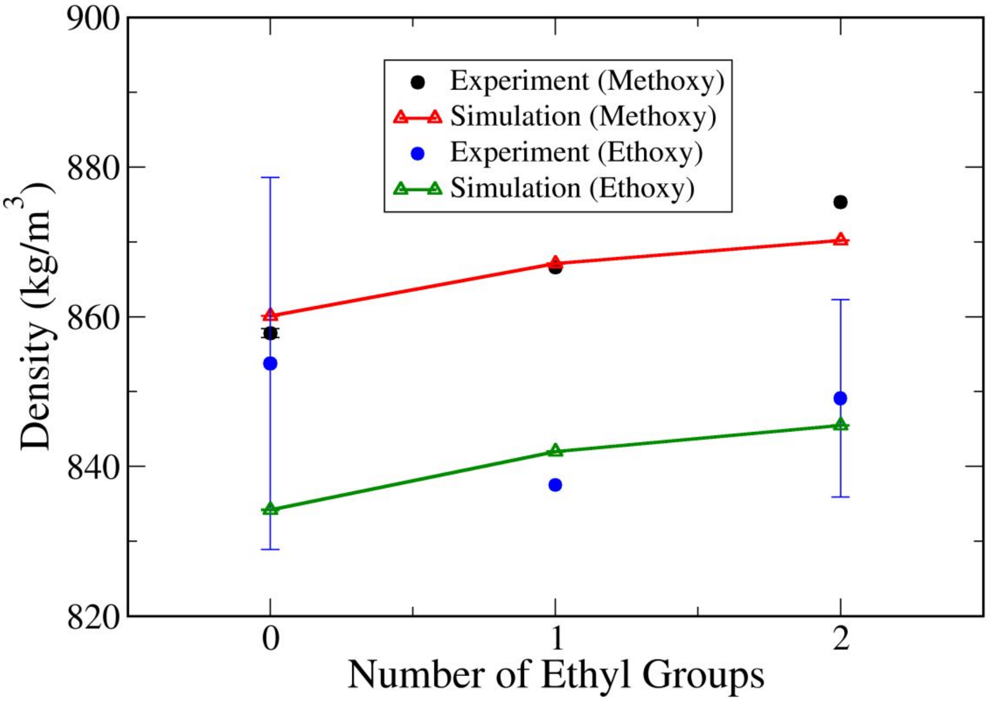

Figure S48 *– Comparison between model predictions (open triangles and lines) and experimental data (full circles) for the density of dialkyldialkoxysilanes with either methoxy (black/red) or ethoxy (blue/green) substituents, plotted as a function of the number of ethyl groups present in the molecule.*

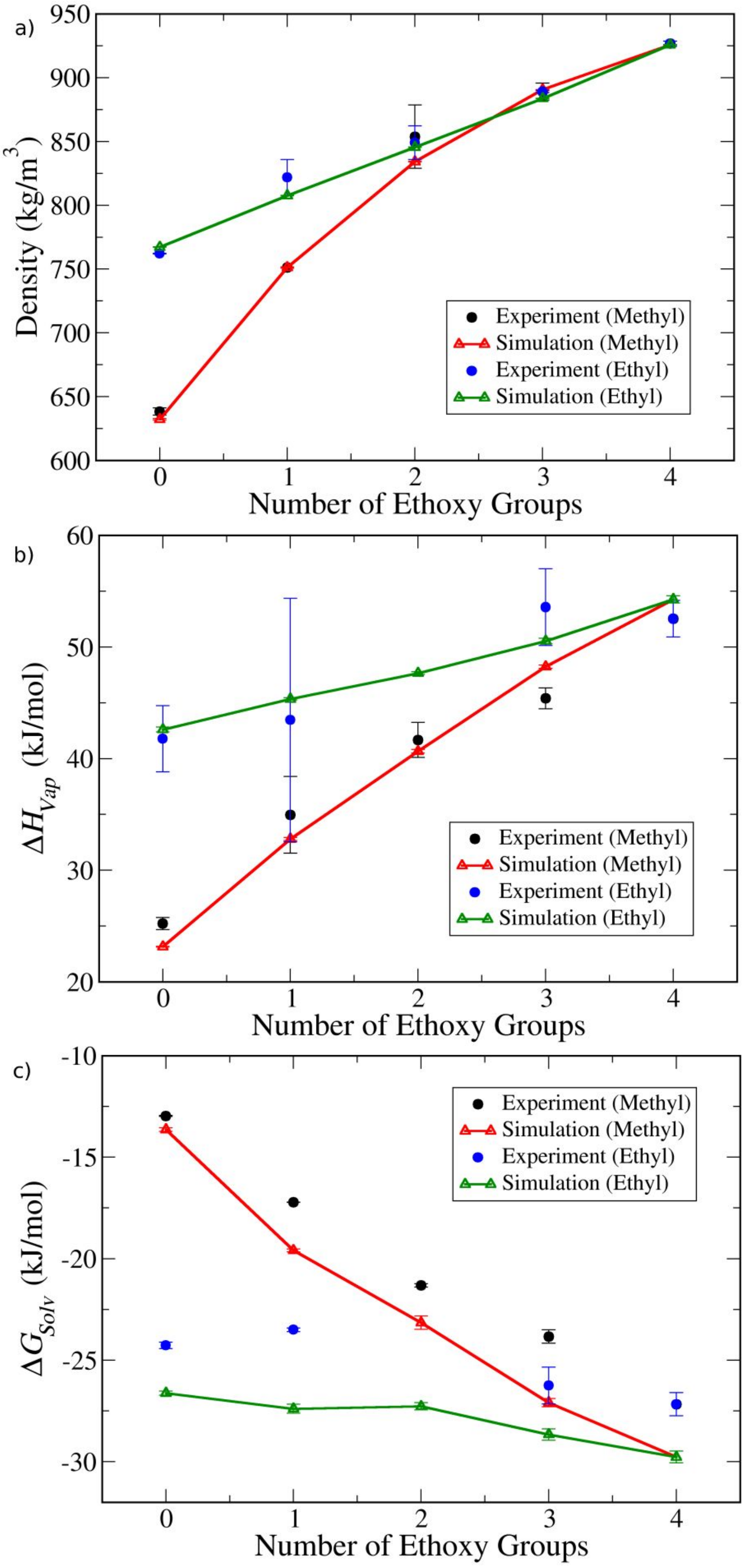

Figure S49 *– Comparison between model predictions (open triangles and lines) and experimental data (full circles) for a) density, b) enthalpy of vaporization, and c) self-solvation free energy. The data is for alkylethoxysilanes with either methyl (black/red) or ethyl (blue/green) substituents, plotted as a function of the number of ethoxy groups present in the molecule.*

***Table S73** – Comparison between simulated and experimental densities for all compounds studied here. Each property value is followed by its uncertainty (±), both in units of kg/m³.*

**Molecule**	**Experimental**	±	**Simulated**	±
Met4Si	638.3	2.8	632.3	0.28
M3E1Si	679.5	0.7	675.2	0.18
M2E2Si	712.1	0.1	712.3	0.11
M1E3Si	739.4		742.5	0.07
Eth4Si	762.1	0.4	767.2	0.08
Met3SiOH	807.4	2.0	807.3	0.06
M2ESiOH	825.0	3.5	826.1	0.09
ME2SiOH	839.3		842.2	0.15
Eth3SiOH	858.0	0.6	856.2	0.07
M3SiOM	753.0	2.7	751.5	0.15
E3SiOM	813.8		815.3	0.1
M3SiOE	751.2	0.4	751.27	0.19
E3SiOE	821.7	14.1	807.6	0.09
M2Si(OM)2	857.8	0.6	860.1	0.05
MESi(OM)2	866.6		867.1	0.05
E2Si(OM)2	875.3		870.2	0.06
M2Si(OE)2	853.8	24.8	834.2	0.09
MESi(OE)2	837.5		842	0.09
E2Si(OE)2	849.1	13.2	845.5	0.07
MSi(OM)3	949.7	1.6	952.74	0.1
MSi(OE)3	889.0	6.6	890.6	0.07
ESi(OM)3	942.4	0.1	938.9	0.08
ESi(OE)3	889.1	0.9	883.7	0.06
Si(OMet)4	1024.2	4.6	1019.9	0.06
Si(OEth)4	926.6	2.0	925.7	0.09
Met6Si2O	758.4	0.8	761.65	0.27

Table S74 *– Comparison between simulated and experimental enthalpies of vaporization for all compounds studied here. Each property value is followed by its uncertainty (±), both in units of kJ/mol. Simulated data are corrected for polarization effects (see main paper for details).*

**Molecule**	**Experimental**	**±**	**Simulated**	**±**
Met4Si	25.2	0.5	23.15	0.02
M3E1Si	30.4	0.5	28.16	0.08
M2E2Si	36.6	4.7	33.05	0.05
M1E3Si	38.6	2.1	38.01	0.06
Eth4Si	41.8	3.0	42.6	0.11
Met3SiOH	45.3	2.6	46.78	0.06
M2ESiOH	41.7		52.7	0.05
ME2SiOH			57.48	0.05
Eth3SiOH	52.5	8.6	62.26	0.09
M3SiOM	30.0		29.53	0.06
E3SiOM			42.48	0.09
M3SiOE	35.0	3.4	32.8	0.07
E3SiOE	43.5	10.9	45.34	0.05
M2Si(OM)2	33.7		34.65	0.04
MESi(OM)2			38.65	0.04
E2Si(OM)2			42.15	0.08
M2Si(OE)2	41.7	1.6	40.65	0.08
MESi(OE)2			44.35	0.08
E2Si(OE)2			47.65	0.06
MSi(OM)3	35.9	3.2	39.72	0.08
MSi(OE)3	45.4	0.9	48.23	0.07
ESi(OM)3	41.9		42.62	0.10
ESi(OE)3	53.6	3.4	50.53	0.12
Si(OMet)4	42.3	1.4	43.05	0.05
Si(OEth)4	52.5	1.6	54.26	0.16
Met6Si2O	37.4	1.0	35.93	0.04

Table S75 *– Comparison between simulated and experimental self-solvation free energies for all compounds studied here. Each property value is followed by its uncertainty (±), both in units of kJ/mol. Simulated data are corrected for polarization effects (see main paper for details). The last two columns report the Lennard-Jones and Electrostatic contributions to the simulated free energy.*

Molecule	Experimental	±	Simulated	±	LJ	Electrostatic
Met4Si	-12.97	0.023	-13.64	0.09	-13.33	-0.31
M3E1Si	-15.93	0.015	-16.75	0.13	-16.49	-0.26
M2E2Si	-18.87	0.32	-19.77	0.15	-19.57	-0.20
M1E3Si	-21.73	0.24	-22.67	0.17	-22.50	-0.17
Eth4Si	-24.27	0.16	-26.67	0.1	-26.55	-0.12
Met3SiOH	-22.14	0.006	-25.08	0.26	-13.56	-10.61
M2ESiOH	-22.87	1.55	-28.07	0.29	-17.38	-9.96
ME2SiOH			-29.04	0.81	-19.95	-8.52
Eth3SiOH	-28.16	1.62	-32.93	0.57	-22.02	-10.51
M3SiOM	-15.62	0.032	-17.88	0.24	-15.58	-1.72
E3SiOM			-25.46	0.16	-23.91	-0.97
M3SiOE	-17.22	0.001	-19.59	0.07	-17.90	-1.15
E3SiOE	-23.50	0.089	-27.39	0.22	-26.30	-0.55
M2Si(OM)2	-17.87	0.034	-20.04	0.13	-16.29	-3.20
MESi(OM)2			-22.29	0.11	-18.87	-2.77
E2Si(OM)2			-24.74	0.11	-21.65	-2.43
M2Si(OE)2	-21.32	0.079	-23.15	0.33	-20.79	-1.71
MESi(OE)2			-25.48	0.1	-23.36	-1.47
E2Si(OE)2			-27.27	0.18	-25.46	-1.16
MSi(OM)3	-19.62	0.040	-22.78	0.17	-16.79	-5.17
MSi(OE)3	-23.83	0.33	-27.09	0.2	-23.74	-2.52
ESi(OM)3	-22.09	0.072	-24.1	0.09	-19.00	-4.28
ESi(OE)3	-26.25	0.91	-28.66	0.28	-25.81	-2.02
Si(OMet)4	-22.54	0.31	-23.72	0.13	-16.80	-6.20
Si(OEth)4	-27.17	0.57	-29.77	0.29	-26.10	-3.02
Met6Si2O	-19.03	0.16	-22.55	0.29	-20.57	-1.86

Table S76 *– Simulated self-diffusion coefficients for all compounds studied here. Each property value is followed by its uncertainty (±), both in units of 10^{-9} m^2/s. Results have not been corrected for finite-size effects.*

**Molecule**	**Simulated**	±
Met4Si	3.30	0.020
M3E1Si	2.54	0.008
M2E2Si	2.00	0.009
M1E3Si	1.57	0.008
Eth4Si	1.18	0.005
Met3SiOH	0.59	0.026
M2ESiOH	0.38	0.022
ME2SiOH	0.29	0.028
Eth3SiOH	0.18	0.037
M3SiOM	2.81	0.029
E3SiOM	1.35	0.013
M3SiOE	2.45	0.003
E3SiOE	1.19	0.001
M2Si(OM)2	2.22	0.009
MESi(OM)2	1.78	0.077
E2Si(OM)2	1.39	0.004
M2Si(OE)2	1.61	0.011
MESi(OE)2	1.30	0.003
E2Si(OE)2	1.06	0.005
MSi(OM)3	1.66	0.012
MSi(OE)3	1.03	0.010
ESi(OM)3	1.41	0.007
ESi(OE)3	0.90	0.005
Si(OMet)4	1.30	0.012
Si(OEth)4	0.71	0.020
Met6Si2O	1.10	0.010

Table S77 *– Comparison between simulated and experimental dielectric constants for all compounds studied here. Simulated data are corrected for polarization effects (see main paper for details).*

**Molecule**	**Experimental**	**Simulated**
Met4Si	1.92	1.877
M3E1Si		1.937
M2E2Si		1.990
M1E3Si		2.029
Eth4Si	2.09	2.057
Met3SiOH	7.17	5.844
M2ESiOH		3.153
ME2SiOH		2.592
Eth3SiOH	2.66	2.445
M3SiOM	3.25	3.369
E3SiOM		3.155
M3SiOE	2.66	3.116
E3SiOE		3.024
M2Si(OM)2	3.66	4.216
MESi(OM)2		4.075
E2Si(OM)2		3.954
M2Si(OE)2	2.69	3.672
MESi(OE)2		3.580
E2Si(OE)2		3.512
MSi(OM)3	4.90	6.646
MSi(OE)3		4.929
ESi(OM)3	3.24	6.041
ESi(OE)3		4.572
Si(OMet)4	6.00	7.124
Si(OEth)4	4.10	4.585
Met6Si2O	2.175	2.073

References

[1] Sharko, P. T.; Besnard, M.; Jonas, J. "Density and Temperature Effects on Vibrational Relaxation in Liquid Tetramethylsilane" *J. Phys. Chem.* **1983**, *87*, 5197-5201.

[2] Yaws, C. L. *The Yaws handbook of physical properties for hydrocarbons and chemicals* **2015**, Second edition, Amsterdam: Elsevier.

[3] Yokoyama, C.; Takagi, T.; Takahashi, S. "Densities of Tetramethylsilane, Tetraethylsilane, and Tetraethoxysilane Under High Pressures" *Int. J. Thermophys.* **1990**, *11*, 477-486.

[4] Bažant, V.; Chvalovský, V; Rathouský, J. *Organosilicon Compounds*, Vol. 2, **1965**, London: Academic Press.

[5] Rochow, E. G. *An Introduction to the Chemistry of the Silicones*, **1946**, New York: John Wiley & Sons, Inc.

[6] Parkhurst, H. J. Jr.; Jonas, J. "Dense liquids. I. The effect of density and temperature on self-diffusion of tetramethylsilane and benzene-d_6" *J. Chem. Phys.*, **1975**, *63*, 2698-2704.

[7] Polyakov, P.; Zhang, M.; Müller-Plathe, F.; Wiegand, S. "Thermal diffusion measurements and simulations of binary mixtures of spherical molecules" *J. Chem. Phys.*, **2007**, *127*, 014502.

[8] Iseard, B. S.; Pedley, J. B.; Treverton, J. A. "Bonding Studies of Organometallic Compounds of Boron and the Group IV Elements. Part VII. Enthalpies of Formation of Hexamethyldisiloxane, Hexamethyldisilane, and Tetraethylsilane by Rotating Bomb Calorimetry" *J. Chem. Soc. A*, **1971**, 3095-3100.

[9] Sugden, S.; Wilkins, H. "XIX. – The parachor and chemical constitution. Part XVI. Silicon compounds" *J. Chem. Soc.*, **1931**, 126-128.

[10] Whitmore, F. C.; Sommer, L. H.; DiGiorgio, P. A.; Strong, W. A.; Van Strien, R. E.; Bailey, D. L.; Hall, H. K.; Pietrusza, E. W.; Kerr, G. T. "Organo-silicon Compounds. I. Synthesis and Properties of n-Alkyltrimethyl- and n-Alkyltriethyl-silanes" *J. Am. Chem. Soc.*, **1946**, *68*, 475–481.

[11] Eaborn, C. *Organosilicon Compounds*, **1960**, London: Butterworths.

[12] Yaws, C. L. *Thermophysical properties of chemicals and hydrocarbons*, **2014**, Second edition, Amsterdam: Elsevier Science.

[13] ChemBK CAS Database, Dimethyldiethylsilane; https://www.chembk.com/en/chem/Dimethyldiethylsilane (last accessed 22/12/2020).

[14] Kato, M.; Tanaka, M. "Ebulliometric Measurement of Vapor-Liquid Equilibria for Four Binary Systems: Methanol + Silicon Tetramethoxide, Methanol + Silicon Tetraethoxide, Ethanol + Silicon Tetramethoxide, and Ethanol + Silicon Tetraethoxide" *J. Chem. Eng. Data* **1989**, *34*, 206–209.

[15] Sauer, R. O. "Derivatives of the Methylchlorosilanes. I. Trimethylsilanol and its Simple Ethers" *J. Am. Chem. Soc.* **1944**, *66*, 1707–1710.

[16] Shorr, L. M. "A New Method of Preparation for Alkoxysilanes" *J. Am. Chem. Soc.,* **1954**, *76*, 1390–1391.

[17] Zhang, Y.; Dong, H.; Wu, C.; Yu, L. "Thermophysical properties of binary mixtures of triethoxysilane, methyltriethoxysilane, vinyltriethoxysilane and 3-mercaptopropyltriethoxysilane with ethylbenzene at various temperatures" *J. Chem. Thermodynamics*, **2014**, *76*, 45–55.

[18] Sommer, L. H.; Pietrusza, E. W.; Whitmore, F. C. "Properties of the Silicon-Hydroxyl Bond in Trialkylsilanols" *J. Am. Chem. Soc.,* **1946**, *68*, 2282–2284.

[19] Boksányi, L.; Liardon, O.; Kováts, E. "Note on the Preparation of Alkyl- and Oxaalkyl-dimethylsilanols" *Helvetica Chim. Acta* **1976**, *59*, 717-727.

[20] Voronkov, M. G.; Baryshok, V. P.; Klyuchnikov, V. A.; Danilova, T. F.; Pepekin, V. I.; Korchagina, A. N.; Khudobin, Yu. I. "Thermochemistry of organosilicon compounds. I. Triorganyl-, tetraorganyl-, organylorganoxy- and tetraorganoxy-silanes" *J. Organometallic Chem.*, **1988**, *345*, 27-38.

[21] Chickos, J. S.; Acree, W. E. "Enthalpies of Vaporization of Organic and Organometallic Compounds, 1880–2002" *J. Phys. Chem. Ref. Data*, **2003**, *32*, 519-878.

[22] Stull, D. R. "Vapor Pressure of Pure Substances: Organic Compounds" *Ind. Eng. Chem.*, **1947**, *39*, 517–540.

[23] The NIST Chemistry Webbook, https://webbook.nist.gov/chemistry/ (last accessed 27/12/2020).

[24] Aston, J. G.; Kennedy, R. M.; Messerly, G. H. "The Heat Capacity and Entropy, Heats of Fusion and Vaporization and the Vapor Pressure of Silicon Tetramethyl" *J. Am. Chem. Soc.*, **1941**, *63*, 2343-2348.

[25] Thomas, L. H.; Smith, H.; Davies, G. H. "Vapour Pressures, Molar Entropies of Vaporisation and Liquid-state Conformation of Alkoxides" *J. Chem. Tech. Biotechnol.* **1980**, *30*, 476-480.

[26] Van der Vis, M. G. M.; Cordfunke, E. H. P. "Tetraethoxysilane, Si(OC2H5)4: vapour pressure measurements at temperatures from 323 to 442 K by means of a Bourdon spoon gauge" *Thermochimica Acta* **1995**, *265*, 129–134.

[27] Jenkins, A. C.; Chambers, G. F. "Vapor Pressures of Silicon Compounds" *Ind. Eng. Chem.* **1954**, *46*, 2367–2369.

[28] Grubb, W. T.; Osthoff, R. C. "Physical Properties of Organosilicon Compounds. II. Trimethylsilanol and Triethylsilanol" *J. Am. Chem. Soc.*, **1953**, *75*, 2230–2232.

[29] Scott, W.; Messerly, J. F.; Todd, S. S.; Guthrie, G. B.; Hosenlopp, I. A.; Moore, R. T.; Osborn, A.; Berg, W. T; McCullough, J. P. "Hexamethyldisiloxane: Chemical

Thermodynamic Properties and Internal Rotation About the Siloxane Linkage" *J. Phys. Chem.*, **1961**, *65*, 1320–1326.

[30] Flaningam, O. L. "Vapor Pressures of Poly(dimethylsiloxane) Oligomers" *J. Chem. Eng. Data*, **1986**, *31*, 266–277.

[31] Holland, R. S.; Smyth, C. P. "The Dielectric Properties and Molecular Structure of Hexamethyldisiloxane" *J. Am. Chem. Soc.* **1955**, *77*, 268–271.

[32] ChemSpider, Search and share chemistry, http://www.chemspider.com/ (last accessed 13/01/2021).

[33] Altshuller, A. P.; Rosenblum, L. "Dielectric Properties of Some Alkylsilanes" *J. Am. Chem. Soc.* **1955**, *77*, 272–274.

[34] Pereira, J. C. G.; Catlow, C. R. A.; Price, G. D.; Almeida, R. M. "Atomistic Modeling of Silica Based Sol-Gel Processes" *J. Sol-Gel Sci. Technol.* **1977**, *8*, 55–58.

[35] Cumper, C. W. N.; Melnikoff, A.; Vogel, A. I. "Physical properties and chemical constitution. Part L. The electric dipole moments of alkoxy-, alkylthio-, and related compounds of carbon, silicon, and germanium" *J. Chem. Soc. A*, **1966**, 323-329.

[36] Matsumura, K. "Dipole Moments of Organosilicon Compounds", *Bull. Chem. Soc. Japan*, **1962**, *35*, 801-808.

[37] Maryott, A. A.; Smith, E. R. "Table of dielectric constants of pure liquids", *US National Bureau of Standards*, Washington : U.S. Government printing Office, **1951**.

[38] Svirbely, W. J.; Lander, J. J. "The Dipole Moments of Diethyl Sulfite, Triethyl Phosphate and Tetraethyl Silicate" *J. Am. Chem. Soc.* **1948**, *70*, 4121–4123.

[39] Cumper, C. W. N.; Melnikoff, A.; Vogel, A. I. "Physical Properties and Chemical Constitution. Part XLVIII. The Electric Dipole Moments of R_3XOMe, $(R_3X)_2O$, R_3XSMe, and $(R_3X)_2S$ where X is C, Si, Ge, or Sn" *J. Chem. Soc. A*, **1966**, 246-249.

[40] Tables for Organic Chemistry, http://www.stenutz.eu/chem/ (last accessed 13/01/2021).

[41] Chvalovský, V; Bažant, V. "Differences between Organic Chemistry of Silicon and some other Group IV Elements" *Helvetica Chim. Acta*, **1969**, *52*, 2398-2417.

[42] Nagy, J.; Ferenczi-Gresz, S.; Farkas, R.; Gábor, T. "Studies on Alcoxysilane-Alcohol and Phenoxysilane-Phenol Binary Systems" *Periodica Polytechnica Chem. Eng.*, **1971**, *15,* 155-167.

[43] Langford, V. S.; Gray, J. D. C.; McEwan, M. J. "Selected ion flow tube studies of several siloxanes" *Rapid Commun. Mass Spectrom.* **2013**, *27*, 700–706.

[44] Manz, T. A.; Limas, N. G. "Introducing DDEC6 atomic population analysis: part 1. Charge partitioning theory and methodology." *RSC Advances* **2016**, *6*, 47771-47801.

[45] Cancès, E.; Mennucci, B.; Tomasi, J. "A new integral equation formalism for the polarizable continuum model: Theoretical background and applications to isotropic and anisotropic dielectrics." *J. Chem. Phys.* **1997**, *107*, 3032–3041.

[46] Grigoras, S.; Lane, T. H. "Molecular mechanics parameters for organosilicon compounds calculated from ab initio computations." *J. Comput. Chem.* **1988**, *9*, 25-39.

[47] Abraham, R. J.; Grant, G. H. "A Molecular Mechanics Study of the Si-O bond and Alkyl-silanes." *J. Comput. Chem.* **1988**, 9, 709-718.

[48] Beagley, B.; Monaghan, J.; Hewitt, T. "Electron-diffraction studies of tetramethylsilane and hexamethyldisilane, and discussion of the lengths of Si-C bonds." *J. Mol. Struct.* **1971**, *8*, 401-411.

[49] Koput, J. "The equilibrium structure and torsional potential energy function of methanol and silanol." *J. Phys. Chem. A* **2000**, *104*, 10017-10022.

[50] Almenningen, A.; Bastiansen, O.; Ewing, V.; Hedberg, K.; Traetteberg, M. "The Molecular Structure of Disiloxane." *Acta Chem. Scand.* **1963**, *17*, 2455-2460.

[51] Koput, J.; Wierzbicki, A. "The large-amplitude motions in quasi-symmetric top molecules with internal C3v rotors: Interpretation of the low frequency Raman spectrum of disiloxane." *J. Mol. Spect.*, **1983**, *99*, 116-132.

[52] Weinhold, F.; West, R. "The Nature of the Silicon-Oxygen Bond." *Organometallics* **2011**, *30*, 5815-5824.

[53] Becke, A. D. "Density-functional thermochemistry. III. The role of exact exchange" *J. Chem. Phys.* **1993**, 98, 5648-5652.

[54] Lee, C.; Yang, W.; Parr, R. G. "Development of the Colle-Salvetti correlation-energy formula into a functional of the electron density" *Phys. Rev. B* **1988**, 37, 785-789.

[55] Dunning, T. H. "Gaussian basis sets for use in correlated molecular calculations. I. The atoms boron through neon and hydrogen" *J. Chem. Phys.* **1989**, *90*, 1007-1023.

[56] Martin, M. G.; Siepmann, J. I. "Transferable potentials for phase equilibria. 1. United-atom description of n-alkanes." *J. Phys. Chem. B* **1998**, *102*, 2569−2577.

[57] Stubbs, J. M.; Potoff, J. J.; Siepmann, J. I. "Transferable potentials for phase equilibria. 6. United-atom description for ethers, glycols, ketones and aldehydes." *J. Phys. Chem. B* **2004**, *108*, 17596-17605.

[58] Horn, H. W.; Swope, W. C.; Pitera, J. W. "Characterization of the TIP4P-Ew water model: Vapor pressure and boiling point" *J. Chem. Phys.* **2005**, *123*, 194504.
